# Sexual selection drives sex difference in adult life expectancy across mammals and birds

**DOI:** 10.1126/sciadv.ady8433

**Published:** 2025-10-01

**Authors:** Johanna Staerk, Dalia A. Conde, Morgane Tidière, Jean-François Lemaître, András Liker, Balázs Vági, Samuel Pavard, Mathieu Giraudeau, Simeon Q. Smeele, Orsolya Vincze, Victor Ronget, Rita da Silva, Zjef Pereboom, Mads F. Bertelsen, Jean-Michel Gaillard, Tamás Székely, Fernando Colchero

**Affiliations:** ^1^ Department of Primate Behavior and Evolution, Max Planck Institute for Evolutionary Anthropology, Leipzig 04103, Germany.; ^2^ Department of Biology, University of Southern Denmark, Odense 5230, Denmark.; ^3^ Population Biology Group, Department of Biology, University of Southern Denmark, Odense 5230, Denmark.; ^4^ Conservation Science Alliance, Species360, Bloomington, MN 55425, USA.; ^5^ Laboratoire de Biométrie et Biologie Evolutive, UMR 5558, CNRS, Université Claude Bernard Lyon 1, Villeurbanne 69310, France.; ^6^ HUN-REN-PE Evolutionary Ecology Research Group, University of Pannonia, Veszprém 8200, Hungary.; ^7^ Behavioral Ecology Research Group, Center for Natural Sciences, University of Pannonia, Veszprém 8200, Hungary.; ^8^ Biodiversity, Climate Change and Water Management Coordination Research Centre, University of Debrecen, Debrecen H-4032, Hungary.; ^9^ HUN-REN–UD Evolution of Reproductive Strategies Research Group, Department of Evolutionary Zoology and Human Biology, University of Debrecen, Debrecen H-4032, Hungary.; ^10^ Eco-anthropologie (EA), Muséum national d'Histoire naturelle, CNRS, Université Paris Cité, Musée de l’Homme 17 place du Trocadéro, Paris 75016, France.; ^11^ French Institute for Demographic Studies (INED), Aubervilliers 93300, France.; ^12^ Littoral, Environnement et Sociétés (LIENSs), UMR 7266 CNRS-La Rochelle Université, La Rochelle 17000, France.; ^13^ Department of Ecoscience, Aarhus University, Aarhus 8000, Denmark.; ^14^ ImmunoConcEpT, CNRS UMR 5164, University of Bordeaux, Bordeaux 33076, France.; ^15^ Wetland Ecology Research Group, HUN-REN Centre for Ecological Research, Debrecen 4026, Hungary.; ^16^ Evolutionary Ecology Group, Hungarian Department of Biology and Ecology, Babeş-Bolyai University, Cluj-Napoca 40006, Romania.; ^17^ Institute of Organismic and Molecular Evolution, Johannes Gutenberg University, Mainz, Germany.; ^18^ CIBIO, Centro de Investigação em Biodiversidade e Recursos Genéticos, InBIO Laboratório Associado, BIOPOLIS Program in Genomics, Biodiversity and Land Planning, Campus de Vairão, Universidade do Porto, Vairão 4485-661, Portugal.; ^19^ Antwerp ZOO Centre for Research and Conservation, Royal Zoological Society of Antwerp, Antwerp 2018, Belgium.; ^20^ Copenhagen Zoo, Frederiksberg 2000, Denmark.; ^21^ University of Copenhagen, Frederiksberg 1870, Denmark.; ^22^ Milner Centre for Evolution, Department of Life Sciences, University of Bath, Bath BA2 7AY, UK.; ^23^ Department of Mathematics and Computer Science, University of Southern Denmark, Odense 5230, Denmark.

## Abstract

Across human cultures and historical periods, women, on average, live longer than men, a pattern best understood from a comparative evolutionary perspective. Here, we analyzed adult life expectancy in 528 mammal and 648 bird species in zoos. Like humans, 72% of mammals exhibited a female life expectancy advantage, while 68% of birds showed a male advantage, as expected from the harmful effects of sex chromosomes described by the heterogametic sex hypothesis. Yet, sex differences varied widely. In zoos, we found strong evidence that this variation generally correlated with both the mating system and sexual size dimorphism. Although with weaker evidence, the patterns remained consistent in populations from the wild, with an even larger effect of the mating system. Thus, even in zoos, where environmental pressures are largely reduced, precopulatory sexual selection seems to play a fundamental role in shaping sex differences in life expectancy in mammals and birds.

## Teaser

Sexual size dimorphism and mating system contribute to male and female life-span differences across wild and zoo populations.

## INTRODUCTION

Both scientists and the general public have long been intrigued by the relatively large and consistent differences in survival between women and men. Worldwide, today, women live, on average, 5.4 years longer than men (*1*). Although the magnitude varies, the direction of this effect is nearly universal in human populations regardless of historical, cultural, or social context (*2*–*5*). While some researchers have argued that this puzzling consistency is attributable to the interplay between human sociality and biology (*6*–*8*), comparative approaches show that the female survival advantage is seemingly common in other mammals (*9*–*12*). However, the female advantage is not universal across animals [e.g., (*5*, *7*, *13*, *14*)]: Males outlive females in many birds, amphibians, and insects (*15*–*20*). Understanding why the direction and strength of sex differences in survival vary across the tree of life requires uncovering the evolutionary mechanisms that underlie them, both within and across major taxonomic groups.

Several nonmutually exclusive hypotheses have been proposed to explain sex differences in adult life expectancy (ALE; i.e., the average age at death in a population) and their evolution. One of the leading hypotheses is based on the deleterious effects of the sex chromosomes in the heterogametic sex (XY in male mammals or ZW in female birds) (*7*, *17*–*19*, *21*–*23*). Alternatively, life history theory suggests that sex biases in survival result from differences in the intensity of sexual selection, as individuals of one sex sacrifice long-term survival by allocating resources to mate competition or to the development and maintenance of sexually selected traits (*17*, *24*–*26*). Another set of explanations focuses on the cost of reproduction, where increased allocation to gestation, offspring production, or parental care can also come at the expense of survival (*27*–*30*).

Resolving the relative explanatory power of these theories and hypotheses requires comparable demographic data spanning a broad phylogenetic scope. The most extensive comparisons to date include 101 mammal species (*9*) and 194 bird species (*15*) from populations in the wild. While evolutionarily informative, observed sex differences may stem from environmental factors, such as sex-biased dispersal or risk-taking behaviors, rather than underlying physiological aging differences (*7*). Moreover, modest sample sizes often limit statistical power and precision in estimating the magnitude of differences. Data from zoo populations provide unique opportunities to study sex-specific survival and life expectancy by markedly expanding the number of species and their sample sizes (*31*, *32*) while reducing confounders like predation, starvation, increased risk of injuries, and pathogen exposure. This partially controlled environment can help isolate the effects of genetic and physiological factors influencing sex-specific ALE (*7*, *9*, *32*).

However, reduced environmental pressures in zoos may also attenuate the strength of sex differences in ALE and their association with other life history variables. Species often live considerably longer in zoos than in the wild (*33*), and abundant resources and managed reproduction in zoos may lower individual survival costs associated with growth and reproduction. For example, the influence of sexual size dimorphism (SSD) on male survival tends to be lower, and the individual cost of producing offspring appears to have no effect on female survival in zoo settings (*12*, *34*). Nevertheless, the goal of any comparative study on sex differences in life expectancy is to understand the evolutionary forces that shape them across species, not within them. If these forces are sufficiently strong, then they should still be evident even in controlled environments. Combining data from populations in both zoos and the wild can help differentiate between evolutionary and ecological factors influencing sex differences in ALE. Consistent results across both settings might suggest that genetic mechanisms rooted in evolutionary processes are involved.

Here, we conducted a large-scale comparative study of sex differences in ALE in mammal and bird populations in zoos and on a subset of species from populations in the wild, to explore the direction and magnitude of these differences between species and within clades (e.g., orders). Our goal was to elucidate whether, as predicted by the heterogametic sex hypothesis, the general pattern of female ALE advantage in mammals and the male advantage in birds is widespread within classes. Motivated by our finding that sex differences in ALE are greatly variable within birds and mammals and across environments, we then tested two influential hypotheses proposed to explain sex differences in survival based on life history characteristics that can vary even between closely related species, namely, the effect of sexual selection and the cost of reproduction.

## RESULTS

### Sex differences in ALE

The heterogametic sex hypothesis proposes shorter life spans for the heterogametic sex, either due to the “unguarded X/Z” chromosome or the “toxic Y/W” (*5*, *17*, *35*). While some evidence supports this hypothesis in its various forms (*18*, *19*, *21*, *22*, *35*), studies have argued that it is insufficient to explain the observed male or female advantage in life expectancy across many species (*36*, *37*). Moreover, substantial variation remains in both the magnitude and direction of sex differences in life expectancy, even among species with the same sex determination system (*9*, *15*). For example, in populations in the wild, female moose (*Alces alces*) have twice the adult median life span of males, while adult male sifakas (*Propithecus verreauxi*) live, on average, 20% longer than females (*9*).

Here, we analyzed sex differences in ALE, hereafter ALE differences, in mammals and birds from zoo populations and, for a subset of species, corresponding populations from the wild, to determine whether the predictions of the heterogametic sex hypothesis hold across both controlled and natural environments. We used Bayesian survival trajectory analysis (*38*–*40*) to fit age- and sex-specific survival models from the age at first birth for each species, using data from the Species360 Zoological Information Management System (ZIMS; Data Use Agreement no. 101333) (*41*). Our analysis encompassed 528 mammal and 648 bird species from zoo populations, spanning the majority of taxonomic orders (20 of 29 mammalian orders and 31 of 42 avian orders), covering taxa generally underrepresented in survival studies, such as shorebirds, insectivores, and rodents. To evaluate the consistency of our findings in natural settings, we also analyzed published data from populations in the wild of 110 species (69 mammals and 41 birds) that were also monitored in zoos.

From the Bayesian survival models, we estimated posterior means and SDs of ALE differences, where ALE is represented by the mean (expected) age at death calculated from the distribution of ages at death (data S1). We calculated ALE differences as δ_*e*_ = (*e*_*f*_ − *e*_*m*_)/max(*e*_*f*_, *e*_*m*_), where *e*_*f*_ and *e*_*m*_ refer to female and male ALE, respectively. This measure ranges from −1 to 1, with 0 indicating no difference, positive values indicating a female advantage, and negative values indicating a male advantage in ALE. Therefore, δ_*e*_ quantifies the proportional differences in ALE relative to the sex with the longer ALE and can be directly expressed as percent differences, given by $P_{\delta }=100\;\times |\delta_{e}|$ . Thus, if $\delta_{e}>0$ , then $P_{\delta }$ is the percent female advantage, while, if $\delta_{e}<0$ , then $P_{\delta }$ is the percent male advantage. For instance, a difference of δ_*e*_ = 0.5 indicates a $P_{\delta }=$ 50% female advantage, while δ_*e*_ *= −*0.5 indicates a $P_{\delta }=$ 50% male advantage. Although highly correlated with other metrics of sex differences in life expectancy [e.g., log(*e*_*m*_/*e*_*f*_); fig. S1], this measure provides a more intuitive understanding of ALE differences. To estimate the strength of evidence in ALE differences (i.e., how different the posterior mean δ_*e*_ is from 0), we calculated a two-sided test statistic (zero overlap) of the area under the posterior density of values smaller or larger than 0, highlighting differences with zero overlap below 0.05 to indicate strong evidence and between 0.05 and 0.125 as moderate evidence. We calculated class and order-level means and SEs as weighted averages, where the weights were determined using the reciprocal of the posterior SD for each species.

#### ALE differences across mammals and birds

**Fig. 1.**
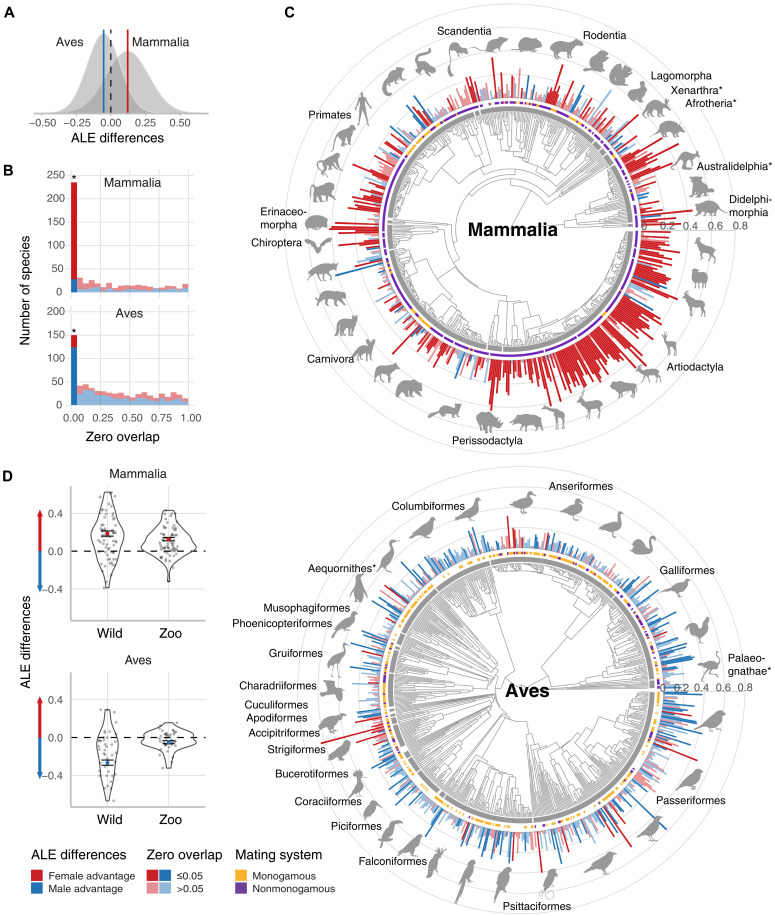
Sex differences in ALE (ALE differences) for mammals and birds. (**A**) Density plot of the distribution and weighted mean (red and blue lines) of ALE differences for birds and mammals. (**B**) Histogram of the number of species according to their zero overlap. Zero overlap provides a two-sided test that indicates the area under the posterior density of ALE differences below or above 0. Differences with zero overlap of ≤0.05 were considered nonnegligible (marked with *). (**C**) Phylogenetic trees of 528 mammal and 648 bird species. Red and blue bars on the outer ring of the phylogenetic tree show the posterior means of ALE differences (δ_*e*_), calculated as female life expectancy minus male life expectancy divided by the maximum life expectancy of either sex. The length of the bars shows the magnitude of δ_*e*_, while the color represents whether the advantage favors females (red) or males (blue). Opacity indicates the strength of evidence for those differences (zero overlap of ≤0.05). The inner tiles indicate whether the species is monogamous (yellow) or not (purple). The inner gray circle and the animal shapes (obtained from phylopic.org) indicate orders (those marked with * represent superorders). (**D**) Species in wild versus zoo environments. Colored points show the class means and SEs weighted by the SEs of the ALE differences.

Our results showed a 12% average female ALE advantage in mammals (class mean δ_*e*_ = 0.122; 95% confidence interval, [0.108, 0.136]) and a 5% average male advantage in birds (class mean δ*_e_* = −0.052, [−0.062, −0.042]) in zoos (Fig. 1A). Notably, this average proportional female advantage in mammals was more than twice as large as the male advantage in birds. Overall, ALE was female biased in 72% of mammal species (381 of 528 species) and male biased in 68% of bird species (438 of 648 species). Note that these numbers include species with weak or no evidence for male-female differences in ALE. We identified 208 mammal species with strong evidence for a female bias (i.e., zero overlap of ≤0.05), representing 39% of all species, and 28 species (5%) with a male bias. Among birds, 122 species (19%) showed robust evidence of a male bias, while 26 species (4%) exhibited a clear female bias (Fig. 1, B and C). We also examined species for which we had a higher level of confidence in the ALE difference estimates as measured by the posterior SD (i.e., lower 20th percentile of the distribution of posterior SDs). We found that mammals had an even larger average female advantage of 16%, whereas birds had a 6% male advantage (δ_*e*_ = 0.156, [0.134, 0.178] for mammals; and δ_*e*_ = −0.058, [−0.076, −0.040] for birds).

The average ALE differences were more pronounced in the wild than in zoos, although with much greater variability (Fig. 1D and figs. S2 and S3). The average female advantage in mammals was 1.5 times greater in wild populations than in zoos (class mean δ_*e*_: 0.186, [0.133, 0.239] versus 0.127, [0.092, 0.162]), while, in birds, the average male ALE advantage was five times greater in the wild than in zoos (class mean δ_*e*_: −0.266, [−0.321, −0.211] versus −0.050, [−0.081, −0.019]; Fig. 1D). Despite the apparent larger ALE differences in wild populations, we found that the direction of sex bias was consistent between both environments for 47 (68%) mammal and 23 (56%) bird species. We recorded strong to moderate evidence (i.e., zero overlap of ≤0.125) of female bias in mammals in both environments in 20 species (29%), while, in birds, only four species (10%) showed strong to moderate evidence of a male bias in ALE differences across both settings.

Contrary to the predictions of the heterogametic sex hypothesis, we found strong evidence (zero overlap of ≤0.05) of zoo-held species with male-biased ALE in 28 mammals (5.3% of 528 species) and with female-biased ALE in 26 birds (4% of 648 species). In populations in the wild, we found strong evidence for a male bias in five mammal species (7.3% of 69 species) and a female bias in one bird species, namely, the canvasback (*Aythya valisineria*, 2.4% of 41 species).

These results suggest that ALE differences cannot be predicted by the heterogametic sex hypothesis alone but are likely influenced by a combination of environmental and genetic factors. Environmentally driven mortality likely amplifies sex differences in the wild, particularly in birds, while many of the physiological and genetic costs shaping these differences seemingly still persist under controlled conditions.

#### ALE differences within orders

**Fig. 2.**
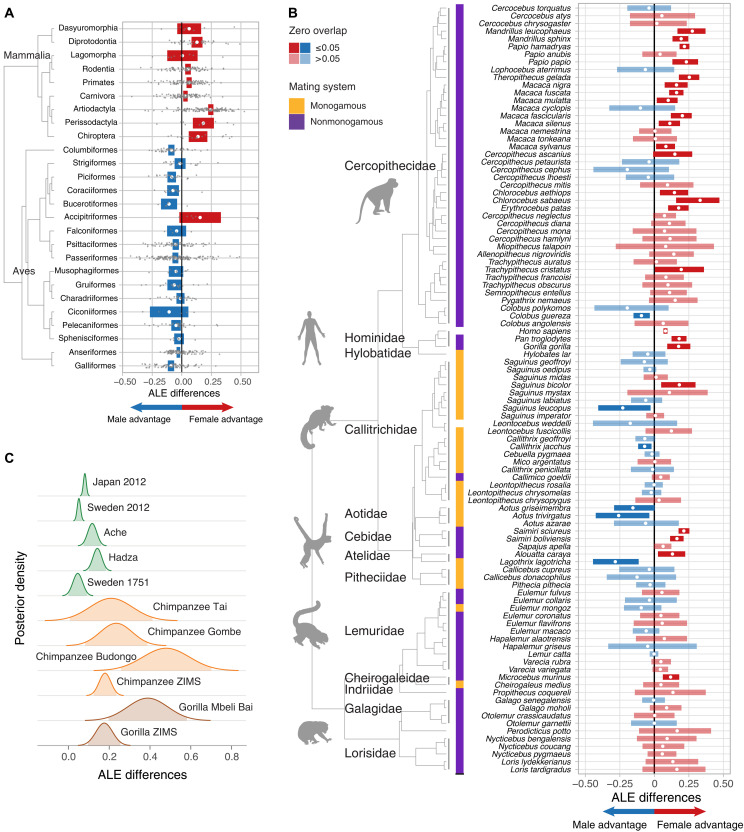
Sex differences in ALE (ALE differences) for taxonomic orders and families. (**A**) Mean (white dots) order-level ALE differences, where horizontal bars show the 95% confidence intervals calculated from the weighted SEs across nine mammalian and 15 bird orders. Only orders with more than five species (gray points) were included. (**B**) ALE differences for 100 primate species. Red and blue bars show the 95% credible intervals, while the white dots show the posterior mean ALE difference. Color represents whether the mean ALE difference favored females (red) or males (blue). Opacity indicates the strength of evidence for those differences (zero overlap of ≤0.05). The tiles indicate whether the species is monogamous (yellow) or not (purple). Animal shapes were obtained from phylopic.org. (**C**) Posterior distribution of ALE differences for different populations of humans, chimpanzees, and gorillas, including the chimpanzee populations from the Taï National Park (Côte d’Ivoire), the Gombe National Park (Tanzania), and the Budongo Forest (Uganda) and the gorilla population from Mbeli Bai (Republic of Congo) (*38*). The shaded areas under the curves show the 95% credible intervals.

We identified considerable between-clade variation in both the magnitude and direction of ALE differences within both vertebrate classes and across environments (Fig. 2A; table S1; and fig. S4, A to P). In zoos, most mammalian orders (17 of 20) exhibited a female ALE advantage, particularly large in ungulates (25% in Artiodactyla and 18% in Perissodactyla), bats (Chiroptera, 14%), and marsupials (superorder Australidelphia and order Didelphimorphia, 12%). The female advantages in ALE differences at the order level were less pronounced in primates (6%), rodents (Rodentia, 5%), and carnivores (Carnivora, 3%), and negligible in rabbits, hares, and pikas (Lagomorpha, 0.6%). We found weak or moderate evidence of male-biased ALE differences in tenrecs and African shrews (Afrosoricida, 3% male advantage), elephant shrews (Macroscelidea, 9%), and anteaters and sloths (Pilosa, 9%).

Among the mammalian orders with the largest number of species, artiodactyls showed the highest proportion of species with a female advantage in ALE differences, while those with the highest proportion of male advantages were carnivores (8 of 102 species), primates (6 of 99 species), and rodents (4 of 86 species). Although species with a male advantage were members of several families within each order, several notable patterns emerged.

In artiodactyls, represented by 136 species in zoos and 25 in the wild, we found strong evidence of a male advantage across both environments in species belonging to the family Bovidae. In the wild, American bison (*Bison bison*) and Dall sheep (*Ovis dalli*) showed strong evidence of a male advantage. Notably, Dall sheep had strong evidence of a female advantage in zoos. In zoos, the sassaby (*Damaliscus lunatus*) and the Arabian gazelle (*Gazella arabica*) showed strong evidence of a male advantage, although these species were not available in our wild dataset.

Among carnivores in zoos, 2 of the 18 species in the family Canidae, wolves (*Canis lupus*) and wild dogs (*Lycaon pictus*), had strong evidence of a male advantage in ALE, while 11 species showed no evidence of ALE differences. These findings were consistent with data from populations in the wild, where wolves again showed strong evidence of a male advantage, wild dogs showed moderate evidence of a male advantage, and red foxes (*Vulpes vulpes*) showed no ALE differences. This shows that a large proportion of species in the family Canidae (more than 70% in zoos) did not conform to the expected mammalian female advantage in ALE.

In primates, δ_*e*_ ranged from a 30% male advantage to a 33% female advantage (Fig. 2B), with strong taxonomic differences across major lineages. For example, we observed a predominantly female ALE advantage in Asian and African monkeys (Cercopithecidae), both in zoo and wild populations, but negligible differences in lemurs (Lemuridae). Among hominids, we estimated a female advantage across all sampled species, although the advantage in humans was less pronounced than in chimpanzee and gorilla populations in zoos and the wild (Fig. 2C). This pattern in humans persisted across historical times and environments, including 18th and 21st century Swedes, 21st century Japanese, and hunter-gatherer populations. In contrast, we found strong evidence of a male advantage in two of the three species of night monkeys (family Aotidae) in zoos, namely, the gray-handed night monkey (*Aotus griseimembra*) and the three-striped night monkey (*Aotus trivirgatus*). The third species, the Azara’s night monkey (*Aotus azarae*), which was also represented in the wild data, showed no ALE differences in either environment, suggesting that night monkeys may not conform to the expected mammalian female advantage.

Analyzing 86 rodent species across 20 families, we found a reduced overall female ALE advantage (order mean δ_*e*_: 0.053, [0.030, 0.077]), albeit with large between-species variation. Although rodents are typically described as fast-paced, with low survival and high annual fecundity, their life histories are diverse. For instance, capybaras and cavies (Caviidae), which are among the largest and slowest-paced rodents, displayed an 11% average female ALE advantage, with strong evidence of a female bias for all five species, which represented this family in our dataset. In contrast, faster-paced species such as mice and rats (Muridae) showed negligible differences (i.e., zero overlap of >0.05 for most species) (fig. S4E). Notably, both species of mole rats (family Bathyergidae), the Damaraland mole rat (*Fukomys damarensis*) and the naked mole rat (*Heterocephalus glaber*), showed strong evidence of a male advantage. In the wild, we only found strong evidence of a male advantage in American red squirrels (*Tamiasciurus hudsonicus*). Despite their importance as model organisms in aging research, rodents show considerable variation in sex differences. This variability also appears within species, as demonstrated by a review of 118 laboratory mouse studies and 19 laboratory rat studies, which found no consistent sex differences, likely due to differences in strain and laboratory environments (*2*).

In birds, 25 of the 31 orders showed an overall male ALE advantage, including all well-represented orders such as galliforms (Galliformes, 9%), pigeons and doves (Columbiformes, 9%), songbirds (Passeriformes, 5%), parrots (Psittaciformes, 5%), and waterfowl (Anseriformes, 3%). Large flightless ratites (ostriches, tinamous, and emus) showed no ALE differences (Rheiformes mean δ_*e*_ = −0.002, [−0.006, 0.002]; *Struthio camelus*, δ_*e*_ = 0.059). Remarkably, the sex chromosomes in ratites are homomorphic (i.e., not highly differentiated) (*42*). However, hawks, eagles, and vultures (Accipitriformes) were a notable exception, with an overall—and, sometimes, large—female ALE advantage (Fig. 2A and table S1).

Inspecting exceptions to the expected male advantage among the bird orders that included a large number of species, we found that 8 of the 151 species of songbirds had strong evidence of a female advantage, followed by waterfowl with 5 of the 106 species. On the other hand, we found no evidence of a female advantage among 48 species of pigeons and doves (Columbiformes) and 23 species of woodpeckers and allies (Piciformes).

In songbirds, the species with strong evidence of a female advantage were spread across different families. In particular, the weavers (family Ploceidae) had two species with strong evidence and one with moderate evidence of a female advantage.

Within waterfowl, we found that, in ducks and geese (family Anatidae), 5 of the 105 species in zoos had strong evidence of a female advantage, while 66 had no evidence of ALE differences. Three of the five species with a female advantage were sea duck species belonging to the *Mergus* and *Bucephala* genera, naturally inhabiting highly northern latitudes under extreme conditions. Overall, 67% of species in this family did not conform to the expected male advantage in birds. Our wild data only included two species in this family, both of which had no evidence of ALE differences.

Although not among the most numerous, birds of prey present notable exceptions to the typical male advantage in birds (Fig. 2A). In zoo populations of falcons and hawks (Accipitridae), two of the five species, the common buzzard (*Buteo buteo*) and Harris’s hawk (*Parabuteo unicinctus*), showed strong evidence of a female advantage, while the remaining species showed no ALE differences. Similarly, in owls (Strigidae), 3 of the 19 species showed a female advantage and nine showed no difference, i.e., 57% did not conform to the expected male advantage. In the wild dataset, four owl species were represented, of which two species deviated from the male-biased pattern: the tawny owl (*Strix aluco*) showed strong evidence of a female advantage, and the little owl (*Athene noctua*) showed no difference. Notably, the female advantage in the tawny owl was only evident in the wild, with a male advantage in zoos.

The above findings demonstrate a widespread female advantage among mammals in both environments, including many taxa previously unexplored. However, in zoos, the number of species that show no or weak evidence of sex differences (292 sp.) or, conversely, a male advantage in ALE (28 sp.) is substantial. In birds, we found a predominant male advantage in ALE, but, again, with notable exceptions. These exceptions in both classes appear to be more prevalent in certain families, which suggest that some phylogenetic groups may have evolved different strategies. To better understand this variability, we therefore investigated how evolutionary mechanisms, such as sexual selection and the cost of reproduction, relate to sex-biased ALE across mammals and birds and across environments.

### Evolutionary drivers of sex differences in ALE

Two hypotheses have been proposed to explain the evolutionary mechanisms, leading to sex differences in ALE, both of which may act simultaneously and revolve around the survival costs associated with reproductive and sexual traits (*17*, *25*, *26*). On the one hand, the sexual selection hypothesis posits that individuals (often males) may prioritize competition for mating or fertilization opportunities over survival, either through investing in sexually selected traits like ornaments or larger body size (i.e., precopulatory competition) (*10*, *15*, *43*) or through the production of high quantity and quality ejaculates (i.e., postcopulatory competition) (*44*). The intensity of sexual selection is expected to be lower in socially monogamous than in polygynous species, where the costs of monopolizing access to mating opportunities can greatly reduce survival [e.g., (*10*, *11*, *43*)]. However, evidence of the direct effect of sexual selection on sex differences in life expectancy from studies in the wild remains inconclusive. For example, in bird and mammal populations in the wild, pre- and postcopulatory traits, such as a polygamous mating system (*10*, *11*, *15*), SSD (*20*), plumage coloration (*20*), and relative testis mass (*15*), have been associated with male-biased mortality. However, other studies have found no such associations or only weak effects (*9*, *12*, *29*, *45*).

On the other hand, the cost of reproduction hypothesis states that the allocation to gestation and parental care can come at a survival cost (*27*, *28*, *30*). For example, in birds, posthatching care in males (*15*, *16*, *29*) and annual egg productivity in females (*16*, *28*) have been associated with sex differences in ALE, albeit other studies failed to find an effect of parental care (*28*). However, some authors argue that, due to prolonged care for altricial offspring, selection may favor higher survival of the caregiving sex, particularly in long-lived species with highly dependent offspring (*46*–*51*). Thus, the respective roles of reproductive costs and parental care in shaping sex differences in ALE remain unresolved.

To test the influence of pre- and postcopulatory sexual selection and the cost of reproduction on ALE differences, we used weighted Bayesian phylogenetic generalized least squares [BPGLS; (*52*)]. As proxies for precopulatory sexual selection, we used as predictors: (i) SSD, included as the additive effect of log-transformed male and female body masses (*45*); (ii) social mating system (monogamy versus nonmonogamy); and (iii) in birds, sexual plumage dichromatism as a measure of allocation to mate attraction (*46*). As proxies for postcopulatory sexual selection, we included log-transformed testis mass, controlled for the potential confounding effects of body mass and mating system [e.g., (*53*, *54*)]. As proxies for the cost of reproduction, we used (i) annual female offspring productivity, following (*55*), and (ii) parental care tactic, categorized as female-biased versus either biparental or male-biased (this last only in birds) (*56*) (see Materials and Methods). Because we used the same predictor variables for birds and mammals (except for plumage dichromatism, which was modeled separately in birds), we ran the BPGLS on both classes combined, using the deviance information criterion (DIC) for model choice (*57*, *58*). To determine whether these effects varied across clades, we conducted separate analyses for each taxonomic order using data from at least 15 species. Due to low sample sizes per order in the wild dataset, this was only possible for the zoo dataset. Furthermore, in the wild dataset, we tested for potential biases in our results due to the effect of hunting.

#### Evolutionary drivers of ALE differences in mammals and birds

The BPGLS results on the zoo data showed that precopulatory sexual selection, specifically mating system and SSD, had a strong effect on ALE differences across both classes, with different class intercepts and a large phylogenetic signal (Pagel’s λ≈0.8 ). Models allowing these effects to differ between classes performed worst, suggesting a phylogenetically widespread influence of precopulatory sexual selection on ALE differences (Table 1 and table S2). Albeit with weaker evidence, these results are consistent with results from populations in the wild, whereby the direction of the effects of mating system and SSD remained, with a stronger influence of mating system in the wild (tables S3 and S4). These results imply that the female ALE advantage is, on average, greatest in nonmonogamous species with male-biased SSD and smallest in monogamous species with low SSD. In zoos, female mammals of polygynous, promiscuous, and polygynandrous species had a 15% female ALE advantage (mean δ_*e*_: 0.149, [0.135, 0.163]), while, in monogamous species, the difference was negligible (mean δ_*e*_: −0.012, [−0.032, 0.008]). In birds, the differences in nonmonogamous species were negligible (mean δ_*e*_: −0.023, [−0.056, 0.010]), and, contrary to mammals, there was a male advantage in monogamous species (mean δ_*e*_: −0.049, [−0.061, −0.037]). These changes from the trend in mammals may result from the background costs of heterogamy on female birds. We found no evidence of an effect of hunting on ALE differences on populations in the wild, although we detected moderate evidence of a further increase in ALE differences with the interaction between hunting and monogamy (table S5). This potential increased effect might be exacerbated by populations exposed to trophy hunting, particularly in certain mammalian orders, where larger and older males are disproportionately removed from the population.

**Table 1.** Parameter estimates of the models fitted using Bayesian PGLS to account for variation observed in ALE differences for mammals and birds. Results show the full models with the lowest DICs for the three tested hypotheses relating to pre- and postcopulatory sexual selection and the cost of reproduction. All other models are provided in table S2. Columns show sample sizes (*N*), posterior mean and SD, and lower and upper 95% credible intervals (CI) from the posterior densities of the regression parameters. Positive coefficients denote an increase in the female advantage and a male advantage otherwise. Zero overlap provides a two-sided test that indicates the area under the posterior density below or above 0. Values in bold indicate zero overlap below 0.05.

Variable	*N*	Mean	SD	Lower CI	Upper CI	Zero overlap
**Precopulatory sexual selection**						
Intercept	848	−0.329	0.075	−0.476	−0.184	**<0.001**
Class Mammalia		0.587	0.091	0.411	0.765	**<0.001**
Log(male body mass)		0.106	0.024	0.059	0.155	**<0.001**
Log(female body mass)		−0.095	0.025	−0.145	−0.046	**<0.001**
Monogamy		−0.063	0.015	−0.092	−0.034	**<0.001**
Residual variance σ^2^		0.029	0.004	0.022	0.039	–
Pagel’s λ		0.770	0.043	0.675	0.843	–
**Postcopulatory sexual selection**						
Intercept	416	−0.375	0.117	−0.605	−0.150	**0.001**
Class Mammalia		0.538	0.128	0.287	0.792	**<0.001**
Log(male body mass)		0.008	0.006	−0.003	0.020	0.167
Log(testis mass)		0.012	0.007	−0.001	0.025	0.088
Monogamy		−0.050	0.020	−0.090	−0.009	**0.015**
Residual variance σ^2^		0.038	0.008	0.024	0.057	–
Pagel’s λ		0.826	0.052	0.708	0.910	–
**Cost of reproduction**						
Intercept	400	−0.325	0.094	−0.507	−0.143	**0.001**
Class Mammalia		0.540	0.115	0.312	0.770	**<0.001**
Log(female body mass)		0.001	0.009	−0.017	0.018	0.934
Log(female age at first birth)		0.024	0.015	−0.005	0.053	0.108
Log(annual productivity)		0.006	0.011	−0.015	0.028	0.560
Female parental care		0.056	0.016	0.023	0.088	**0.001**
Residual variance σ^2^		0.033	0.006	0.022	0.046	–
Pagel’s λ		0.837	0.044	0.737	0.907	–

We still found exceptions whereby none of these mechanisms explained our results. Lemurs, despite being predominantly nonmonogamous, showed negligible ALE differences, confirming observations in wild populations. Lemurs appear to be exceptional among primates, exhibiting balanced sex ratios, a lack of SSD, female social dominance, and genital masculinization (*59*). The female advantage in raptors occurs despite reversed SSD (females are larger than males) and common female territorial defense (*60*, *61*). This suggests that these traits may not be appropriate markers of the strength of sexual selection in these species, or that other processes may be at play (e.g., environmental factors, genetics, or unmeasured survival costs on males).

Although we found no effect of plumage coloration on ALE differences in zoos, there was strong evidence of a negative effect in the wild data. In relation to postcopulatory sexual selection, we found no evidence that sperm competition is associated with sex differences, except in artiodactyls.

Last, contrary to the cost of reproduction hypothesis, we found that female allocation to parental care was positively associated with a female ALE advantage in zoos (Table 1 and table S2). Albeit with moderate evidence (zero overlap of 0.128), we found that the magnitude of the positive effect of female care was larger in the wild (δ_*e*_ = 0.139) than in the zoo populations (tables S3 and S4). We found no evidence of an effect of annual productivity on ALE differences in both environments.

#### Evolutionary drivers of ALE differences within orders

**Fig. 3.**
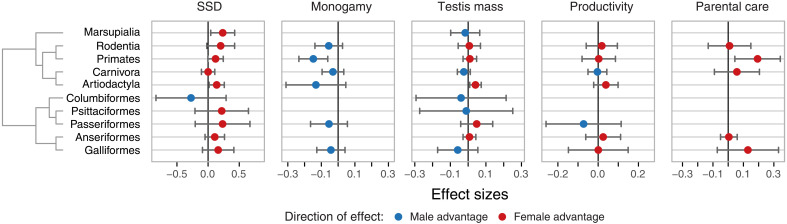
Order-level weighted means of effect sizes for the different evolutionary predictors of sex differences in ALE. The points show the posterior means, and the horizontal bars show the 95% credible intervals calculated from the weighted SDs. Only orders with more than 15 species were analyzed. Colors indicate whether the direction of the mean value of the effect size was positive, favoring a female ALE advantage, or negative, favoring a male ALE advantage. SSD refers to sexual size dimorphism, and productivity refers to the product of mean neonate or egg mass, litter or clutch size, and the mean number of litters or clutches per year per adult female. Marsupialia include the orders Dasyuromorphia, Didelphimorphia, Diprotodontia, and Peramelemorphia.

At the order level, the direction (i.e., sign) of the effect sizes for mating system and SSD was consistent across most mammalian and bird orders. However, in pigeons and doves among birds, male-biased SSD had a negligible negative effect on ALE differences (Fig. 3 and table S6). Among mammals, we found strong evidence of strong positive effects of male-biased SSD in artiodactyls and marsupials, and moderate evidence in rodents and primates. We also found a positive effect of SSD on the remaining bird orders, albeit the evidence was moderate to weak. In primates, the mating system strongly predicted ALE differences, with an average 10% female ALE advantage in nonmonogamous species. In contrast, we found evidence for the effect of postcopulatory sexual selection on sex differences in ALE only in artiodactyls, where relative testis mass was positively associated with a female ALE advantage (Fig. 3 and table S6). As for the cost of reproduction, we found strong evidence of a positive effect of parental care only in primates. This indicates a greater female ALE advantage in primates with greater female allocation to parental care.

We recognize that the confidence intervals for the order-level effects of SSD and monogamy on ALE differences include zero for several orders (Fig. 3). This is expected, as the reduction in sample sizes at the order level and modest effect sizes naturally lead to a reduced strength of evidence of these effects (*62*), as measured by the percentile of 0 on the parameter posterior density. However, we believe that these effects should not be dismissed, as the direction (i.e., a positive effect of SSD and a negative effect of monogamy) and, to a large extent, their magnitudes remained broadly consistent across taxonomic levels (orders and classes) and under varying environmental conditions (zoo and wild).

## DISCUSSION

Our analyses provide the most phylogenetically comprehensive estimates of sex differences in ALE in mammals and birds to date, pointing to evolutionary drivers of sex-specific survival. While our results partially support the heterogametic sex hypothesis, heterogamy alone cannot explain the breadth of variation in ALE differences found here. For instance, in howler monkeys, marmosets, and tamarins, despite male heterogamy, we found negligible female ALE advantages or even male-biased ALE, most likely explained by the widespread monogamy in this group. Our findings suggest an interplay between heterogamy and precopulatory sexual selection, which may also explain the less pronounced male ALE advantage in birds compared to the stronger female ALE advantage in mammals (*18*, *21*, *22*).

Of the two hypotheses we tested, we found that the social mating system, typified as social monogamy versus nonmonogamy (i.e., polygynandrous, polyandrous, promiscuous, or polygynous mating systems), was the most consistent correlate of ALE differences across both mammals and birds. These findings are consistent with early work in mammals (*10*, *11*) and ruminants in zoos (*43*) but differ from results in ungulates from wild populations and broader mammalian datasets, which found no such association (*9*, *12*, *45*). Notably, some of these apparent discrepancies can be at least partially resolved by exploring the taxonomic representation in the previous studies. For instance, the study by Toïgo and Gaillard (*12*), which reports no effect of the mating system, focuses only on wild populations of ungulates (Artiodactyla and Perissodactyla). Here, consistently with their results, we found moderate evidence of a reduction in ALE differences as a function of monogamy in ungulates. It is important to note, however, that there are only a handful of monogamous species in these taxonomic groups, which undoubtedly hinders the detection of any effect. Similarly, in (*9*), despite the larger taxonomic scope, nearly half of the species are ruminants, while only 6 of the 98 species were classified as monogamous, making it extremely unlikely to detect an effect of monogamy. In birds, our results are consistent with previous analyses, which have shown that male polygamy is associated with an increased male bias in mortality (*15*, *28*). Thus, despite the coarse classification of complex patterns of mating behavior and social structure used here to describe the mating system, the larger taxonomic coverage and the shared direction and magnitude of the effect in both classes and across orders and environments (zoo versus wild) suggests that monogamy and a reduction in the female ALE advantage in mammals or an increase in the male advantage in birds may have jointly evolved.

Similarly, our findings on the positive association of SSD and ALE differences are consistent with early evidence in mammals and birds (*10*, *20*), although subsequent studies report a moderate effect in mammals (*9*), no effect in both classes (*12*, *28*, *29*, *45*), or even an opposite effect in birds, whereby a larger male bias in size increased male survival (*15*). Note, however, as we stress above, some of these studies on mammals include a limited number of species and are heavily taxonomically clustered. Nonetheless, the discrepancies between our results and the literature in birds are likely due to a weaker effect of SSD on ALE differences in this class, as we find when using an interaction between the class and this predictor on both the zoo and wild datasets (tables S2 and S3). Alternatively, plumage coloration has been suggested as an appropriate measure of the male investment in sexual characteristics and as a measure of intrasexual selection through mate choice in birds (*5*, *10*, *20*). However, consistent with (*29*), we found no evidence of an effect of plumage coloration on ALE differences in zoos, while, in the wild, the relationship appeared negative, as male bias in plumage dichromatism increased, the male advantage in ALE also increased (table S3). This counterintuitive result contrasts with previous studies where different measures of sex differences in mortality have been positively associated with plumage coloration (*20*, *63*) or found no association between adult sex ratios and plumage dichromatism (*64*). Notably, our result was largely driven by data on geese and ducks (Anseriformes) and was not observed in other groups (table S7). While intriguing and worth exploring further, these results should be interpreted with caution. Most of the wild data for geese and ducks comes from heavily hunted populations, where potential hunting biases in sex-specific mortality remain unclear.

Previous studies have confirmed a mortality cost of reproduction in birds, through posthatching care in males (*15*, *16*, *29*) and annual egg productivity in females (*16*, *28*), while others failed to find an effect of parental care (*28*). Although our result on the positive effect of female care on ALE differences thus appears counterintuitive, it may stem from the interplay between parental care and the mating system. Species with female-only care tend to be polygynous (fig. S6); thus, males are experiencing increased costs of sexual selection, potentially leading to an increased female advantage. Additionally, our combined measure of parental care may mask the specific effect of posthatching care in males, while reproductive management in zoos may further attenuate the costs of reproduction. Alternatively, our results may support the hypothesis that selection favors increased adult survival for the caregiving sex, particularly in species with substantial allocation of time and energy to offspring care (*46*, *47*, *49*, *51*). Our study, however, cannot resolve the two competing hypotheses. For example, primates, for which the selection for longer life span in the caregiving sex hypothesis has been proposed (*47*), are the only mammalian order where female-only care had a positive effect on ALE differences.

In zoos, advances in husbandry and veterinary care, combined with protection from environmental stressors (e.g., harsh climate, starvation, and predation), allow zoo animals to live significantly longer than their wild counterparts (fig. S5). While both sexes benefit from these improvements, we know little about how zoo environments differentially affect males and females. Although zoos attempt to replicate natural settings, they are constrained, for instance, by limited space and access to natural diets. This may be particularly important for species with complex or highly specialized diets, complex social structures, or those less adapted to the higher population densities in zoos, especially for solitary or pair-living species (*65*). For example, Müller *et al*. (*65*) found that diet in zoos influenced female, but not male relative life expectancy in ruminants. Note that, although we found reduced ALE differences in zoo ruminants, the female advantage found in wild populations still remained. On the other hand, reproductive management in zoos, often targeting one sex (e.g., hormonal contraception in female primates), may further influence our results.

Thus, our results from both wild and zoo populations suggest that the effect of sexual selection on the evolution of ALE differences may still be at play and evident even in controlled environments. From a comparative perspective, finding evidence in support of hypotheses on the evolution of ALE differences from data where effects are expected to be attenuated, such as those from zoo populations, warrants further investigation on the genetic basis of the evolution of sex differences in life span.

Last, our findings may help explain why differences in ALE between men and women are so consistent across time and cultures. Specifically, female-biased ALE appears to be common to chimpanzees and gorillas, suggesting that longer life expectancies for females are a characteristic long embedded in our evolutionary history. Intriguingly, though, the female ALE advantage in humans is smaller than that observed in African apes, including previously studied populations of chimpanzees and gorillas in the wild (Fig. 2C). This observation is consistent with the idea of weaker sex-biased sexual selection in humans compared to gorillas and chimpanzees, which exhibit marked or even extreme male-biased size dimorphism, are highly nonmonogamous compared to humans, and, in the case of chimpanzees, have larger relative testes size, suggesting sperm competition as a stronger selective force (*66*). However, the case in humans does suggest the importance of environmental modifiers of sex differences in ALE. For instance, in Sweden, the female ALE advantage was lower in the mid-1700s compared to the 2000s, perhaps because reductions in the risk of maternal death during childbirth have removed otherwise leveling effects on life span between men and women (*67*, *68*). Irrespective of this variation, humans conform well to the general mammalian trend of a female advantage in ALE, demonstrating that, at least in terms of sex differences in survival, our species is not unique.

## MATERIALS AND METHODS

### Survival analysis

We obtained individual birth and death records for birds and mammals via research request no. 101333 from Species360’s ZIMS (*41*). ZIMS is recognized as the most comprehensive global demographic database for animals held under human care, curated by Species360, a global nonprofit organization encompassing more than 1300 zoo and aquarium members across more than 100 countries. Given that Species360 member institutions regularly engage in exchanges and interbreeding among their animal populations, individuals of a collectively managed taxa can be considered part of a single meta-population (*69*). Although our data encompass a broad range of taxonomic orders, species held in zoos may represent an unbalanced sample. Zoos often prioritize species that are easier to breed, more attractive to visitors, or of particular conservation concern. For example, while our sample includes 20% of all extant primate species, it only includes 1% of bat species.

To estimate ALE for each species, we first used the ZIMS data to determine sex-specific ages at first reproduction, measured as the lower 10% quantile of individual ages at first offspring birth or hatching. This quantile level best corresponded to reported ages at birth from literature data, where the intercept was closest to 0 and the slope was closest to 1. If ages at first birth could not be estimated from the ZIMS data, then we used ages at first reproduction from the literature, either sex-specific or non–sex-specific values, in that order of preference. Due to the lack of data on ages at first reproduction in some birds, we used imputed data from (*70*) for 134 species (data S1). Imputed data were only used as a proxy for the minimum age for the survival analyses from which we estimated ALE, but not as life history covariates in the regression analysis to test our hypotheses.

To infer age-specific survival and mortality using ZIMS census data, we used Bayesian survival trajectory analysis using the R package BaSTA (BaSTA version 2.0.0) (*38*–*40*). This method allowed us to include individuals even in cases where ages were missing or when birth dates were uncertain, thus expanding our dataset and enhancing analysis robustness. To reduce uncertainty in the mortality estimates, we only included species with at least 35 individuals per sex (i.e., males and females), with a median sample size of 280 individuals per sex. Records included animals held between 1 January 1980 and 22 January 2024, the date of data extraction. The 1980s threshold was selected due to substantial improvements in record-keeping and husbandry practices in most zoological institutions from this time onward. Outliers, including animals with life spans exceeding the 99th percentile, were excluded.

Although the Gompertz model is usually considered the most parsimonious for modeling age-specific survival in adulthood, it has been shown to underestimate rates of actuarial senescence because of a delayed onset of senescence in slow-living species [e.g., (*9*, *71*)]. To account for this, we fitted Siler models (*72*) from the age at first reproduction, which accounts for the delayed onset of actuarial senescence, for each species and sex. For comparability of the results between sexes, we used the maximum age at first reproduction between males and females (or unknown sex if not available) for both sexes. The Siler model describes mortality as a convex function of age, *x*, given by

μ(x)=exp(a0−a1x)+c+exp(b0+b1x) (1)

where *a*_0_, *b*_0_ ∈ (−∞, ∞) and *a*_1_ *c*, *b*_1_ > 0 are the mortality parameters to be estimated. The first exponential term in Eq. 1 allows for an initial decline in mortality (controlled by parameters *a*_0_ and *a*_1_), followed by *c*, which describes constant background mortality, while the second exponential term captures the increase in mortality due to senescence at older age (controlled by *b*_0_ and *b*_1_).

The cumulative survival function calculated from birth is given by

S(x)=exp[−∫0xμ(t)dt] (2)

BaSTA uses Markov chain Monte Carlo (MCMC) with Metropolis-Hastings sampling (*73*, *74*) to estimate the unknown mortality parameters and unknown birth dates. We ran eight parallel chains for 60,000 iterations, with a burn-in of 10,001 and thinning at intervals of 100 iterations. Model convergence was assessed using the Gelman-Rubin statistic (*75*). We then constructed the posterior densities of the estimated parameters and life expectancies, while traces and goodness-of-fit were visually assessed by at least two independent researchers (figs. S7 and S8). After discarding models with poor goodness-of-fit or for which the life table survival did not reach a minimum threshold of 0.1, we obtained a final dataset and survival and mortality estimates for 648 bird species and 528 mammal species for subsequent analysis.

For human populations, we obtained period life tables for Japan (2012) and Sweden (1750 and 2012) from the Human Mortality Database (*1*), while the Hadza (*76*) and Ache data (*77*) were obtained from published sources. We selected Japan because of its status as the world’s life expectancy leader and Sweden for its high-quality demographic data dating back to the mid-18th century. To obtain the same type of survival results as for the ZIMS data, we reconstructed the human populations based on the life table survival, *l_x_*, by obtaining the number of individuals alive at the beginning of each age interval as *N_x_* = ⌊*N l_x_*⌋ (i.e., nearest lower integer), where *N* is the total number of individuals (assuming a cohort structure). We then estimated the number of individuals dying within the age interval as *D_x_* = *N_x_* − *N*_*x*+1_. We created a simulated dataset with *D_x_* ages at death for each age *x*, which we analyzed using BaSTA. All life tables are available in the GitHub repository (data S1).

Throughout this manuscript, we refer to sex as a binary biological variable (female and male), including in reference to human data. However, we acknowledge that phenotypic traits within sex can be multifaceted and that gender identity in humans may not align with biological sex (*78*).

We obtained published demographic data of populations from the wild (data S1). For mammals, age-specific mortalities were calculated either from life tables obtained from the malddaba database (*79*) or derived from the Siler mortality parameters reported in (*9*). Due to the limited availability of age-specific demographic data for birds, we used published mean annual adult sex-specific or mortality probability, $q_{a}$ , or its complement, the adult annual survival probability, $p_{a}=1-q_{a}$ . When data from multiple populations were available, we selected the population with the highest data quality (i.e., largest sample sizes and longitudinal rather than cross-sectional studies). For comparison with human populations (Fig. 2C), data for multiple chimpanzee populations and the Mbeli Bai gorillas in the wild were obtained from (*38*).

For the published data on populations in the wild, which consisted of reported age-specific mortality parameters from Lemaître *et al.* (*9*), we simulated ages at death by randomly sampling from the corresponding distribution of ages at death derived from the reported parameters and the reported sample sizes. We then analyzed the simulated data using BaSTA, which allowed us to estimate SEs of the life-span metrics relevant for this study.

### Life span metrics

From each iteration of the converged MCMC mortality parameter chains, we calculated adult remaining life expectancies as a metric of life span, that is, the theoretical average age at death, from age at first birth, α, given by

ei=∫α∞S(t)dtS(α) (3)

where *e_i_* is the ALE for sex *i* at a given iteration of the converged MCMC chain. From these, we calculated sex differences in ALE, as

$$\begin{array}{c} \delta_{e}=\frac{\left(e_{f}-e_{m}\right)}{\text{max}\left(e_{f},e_{m}\right)} \end{array}$$ (4)

where *e*_*f*_ refers to female ALE and *e*_*m*_ refers to male ALE. From the resulting posterior vector of ALE differences, we calculated the posterior mean, SD, and 95% credibility intervals of ALE differences for each species (data S1).

Note that positive δ_*e*_ values indicate a female ALE advantage, while negative values indicate a male ALE advantage, with values theoretically ranging from −1 to 1. Therefore, δ_*e*_ quantifies the proportional differences in ALE as a function of the sex with the longest ALE, which can directly be expressed as percent differences (i.e., 100 δ_*e*_). Note that, although highly correlated with previously used metrics of sex differences in longevity [e.g., log(*e*_*m*_/*e*_*f*_); fig. S1], this measure provides a more intuitive understanding of differences in ALE.

As a measure of certainty in the species-level ALE differences (i.e., how different δ_*e*_ was from 0), we calculated a two-sided test statistic (i.e., the zero overlap) of the area under the posterior density of values smaller or larger than 0. For ease of interpretation, we highlighted when the differences in ALE had a zero overlap below 0.05.

For the published data on populations in the wild that consisted of reported sex-specific adult annual mortality probabilities, *q*_*a*_, or annual survival probabilities, *p*_*a*_, we approximated the life table survival, *l_x_*, as

$$\begin{array}{c} l_{x}=\prod_{t=0}^{x-1}\left(1-q_{a}\right)=\prod_{t=0}^{x-1}\;p_{a} \end{array}$$ (5)

which assumes a constant hazard rate given by

$$\begin{array}{c} \mu \left(x\right)=b=-\frac{1}{\text{log}\;\left(1-q_{a}\right)\;} \end{array}$$ (6)

Note that we assumed that age 0 was the age at first reproduction as reported in the corresponding literature source. We then used an approximation for ALE given by

ei≈∑x=0ωlx (7)

where ω is the maximum age for the species. From these estimates, we approximated ALE differences as in Eq. 4. To estimate the life expectancy SEs, we used the variance approximation proposed by (*80*), given by

$$\begin{array}{c} \sigma_{e}^{2}\approx \sum_{x=0}^{\omega -1}l_{x}^{2}{\left[\left(1-a_{x}\right)+e_{x+1}\;\right]}^{2}\left[\frac{q_{a}^{2}\left(1-q_{a}\right)}{D_{x}}\right] \end{array}$$ (8)

where $a_{x}=0.5$ is the fraction of the age interval lived, $e_{x+1}$ is the remaining life expectancy at age *x* + 1, and *D_x_* is the estimated number of individuals dying in the interval, calculated as for the human life tables. To ensure comparability between wild and zoo data, we recalculated the ALEs in zoos from the same age at first reproduction as in the wild populations.

### Phylogenetic generalized least squares

We implemented weighted BPGLS with the R package BayesPGLS (*52*) between the ALE differences, δ_*e*_, and life-history predictors associated with our three evolutionary hypotheses (data S1). We obtained the phylogenetic tree from (*81*, *82*) for mammals and (*83*) for birds. We computed the maximum clade credibility using the phangorn R package (*84*) from a sample of 100 trees based on the birth-death node-dated trees for mammals and the Ericson All Species Tree using the VertLife Phylogeny subsets tool (vertlife.org). For BPGLS with both classes combined, we grafted the two phylogenies using the R package geiger (*85*), assuming a median divergence time of 319 MY, as provided by (https://timetree.org/).

The package BayesPGLS uses MCMC with direct sampling for the regression parameters with Metropolis-Hastings to estimate Pagel’s λ (0 ≤ λ ≤ 1), which provides an estimate of the intensity of the phylogenetic signal (*86*). Given that we used estimated ALE differences from Bayesian survival trajectory analyses on species with different sample sizes and other sources of uncertainty, we used the posterior SEs of δ_*e*_, σ_δ_, to calculate weights for the regression. Given the spread of the SEs, we first transformed them as

υδ=log[1σδ+1] (9)

and calculated the weights as

ωδ=υδmax(υδ) (10)

where ω_δ_ ∈ (0, 1]. The priors for the regression parameters were all normally distributed with mean of 0 and variance of 100.

Note that, for some of the models tested, the predictors for mammals and birds had the same structure (i.e., SSD, mating system, testis mass, body mass, annual productivity, and parental care). In those cases, we ran BPGLS on a combined phylogeny, testing different levels of interactions between the predictors and taxonomic class (i.e., Mammalia and Aves) and compared model performance by means of the DIC (*57*, *58*). In case of models that included plumage dichromatism, which is only available in birds, we ran separate BPGLS in birds. Predictors plotted against ALE differences are provided in figs. S6, S9, and S10.

All analyses were performed in the free open-source software R (*87*). In addition to the R packages BaSTA and BayesPGLS, we used the packages snowfall (*88*), snow (*89*), caper (*90*), phytools (*91*), and geiger (*85*). For the plots we used ggplot2 (*92*), and, for the phylogenetic tree plot, we used ggtree (*93*–*97*), ggtreeExtra (*98*), ggnewscale (*99*), and treeio (*100*).

### Life history covariates

For each species, we calculated a single representative value per life-history variable by computing the mean of all available records for that species. To ensure consistency in species nomenclature across various sources, we standardized species names according to the taxonomic backbone maintained by the Global Biodiversity Information Facility (*101*). This standardization process was facilitated using the R package “taxize” (*102*). Life-history data are available in data S1.

Life-history covariates to test the influence of precopulatory sexual selection included mating system, SSD, and plumage dichromatism (only in birds). In mammals, the social mating system was scored as 0 for nonmonogamous species (including polygamous, promiscuous, and polygynandrous species) and 1 for monogamous species, whereas most of data was obtained from (*103*) and then complemented with additional data from literature searches. In birds, we transformed male and female monogamy scores ranging from 0 to 4 (*56*) into comparable categories (fig. S11). For each sex in birds, we coded species as monogamous (*1*) or nonmonogamous (0), where monogamy was defined as more than 80% of individuals in a species being socially monogamous. Conversely, nonmonogamy was defined as less than 80% of individuals being monogamous. We then classified the species as monogamous if both sexes were monogamous, and, thus, nonmonogamous included polyandrous, polygynous, promiscuous, and polygynandrous species. To test whether our results were robust, we also conducted analyses on the ungrouped monogamy scores for both sexes, as well as alternative thresholds of 95 and 99% for the monogamy category (fig. S11). We chose the 80% threshold for monogamy based on model selection using the DIC. However, results were similar across different threshold values (table S8).

We estimated mean male and female body mass from individual body mass measurements recorded in ZIMS (*41*) and complemented these with information from relevant literature sources. Several measures of SSD have been suggested [see (*43*) for a comparison]. Rather than analyzing residuals, we used log-transformed male and female body masses, used as additive covariates in the BPGLS [e.g., (*45*)] as advocated in (*104*). The allometric relation between the male and the female body mass is assumed to follow the power function

$$\begin{array}{c} M_{m}=\alpha \;M_{f}^{\gamma } \end{array}$$ (11)

where *M*_*m*_ and *M*_*f*_ are the male and female body masses, and α and γ>0 are the scale and power parameters, respectively. If γ=1 , then the body masses are proportional; however, it has been shown that in most mammals the power parameter is γ>1 (*105*). To estimate both parameters, Eq. 11 can be linearized as

$$\begin{array}{c} \text{log}\;\left(M_{m}\right)=\;\text{log}\;\alpha +\gamma \;\text{log}\;\left(M_{f}\right) \end{array}$$ (12)

which is commonly used as the basis to estimate the parameters by means of a linear regression or phylogenetic least squares. From Eq. 12, SSD can then be calculated as

$$\text{SSD}=\text{log}\;\alpha =\text{log}\;\left(M_{m}\right)-\gamma \;\text{log}\;\left(M_{f}\right)$$

which has been used as a predictor in studies of sex differences in survival and other life-history variables as

$$\begin{array}{c} \hat{Y}=\eta +\beta \;\text{SSD}=\eta +\beta \left[\text{log}\;\left(M_{m}\right)-\gamma \;\text{log}\;\left(M_{f}\right)\right] \end{array}$$ (13)

where $\hat{Y}$ is the expected value for the response, η is the intercept, and β is a slope parameter. However, as proposed in (*106*), in our BPGLS, we did not previously estimate the two power function parameters but used the logged body masses as predictors. Thus, we have that we are incorporating SSD as

$$\begin{array}{c} \hat{Y}=\eta +\beta_{1}\;\text{log}\;\left(M_{m}\right)+\beta_{2}\;\text{log}\;\left(M_{f}\right) \end{array}$$ (14)

which, as we show from our results (Table 1 and tables S2 to S4 and S6), the regression parameters are $\beta_{1}>0$ and $\beta_{2}<0$ . By equating the right-hand sides of Eqs. 13 and 14, it is simple to show that $\beta_{1}=\beta$ and $\beta_{2}=-\beta \gamma$ ; thus, the estimated power parameter can be obtained from the BPGLS results as $\gamma =-\beta_{2}/\beta_{1}$ . In short, the effect of SSD on ALE differences is measured by the regression parameter associated with the male body mass ( $\beta_{1}$).

We extracted plumage dichromatism in birds from (*56*), which was measured as the mean relative brightness and patterning across five separately scored body regions, in the interval [−2, 2], where −2 refers to brighter/more patterned females, 0 denotes no difference, and +2 refers to brighter/more patterned males [see methods in (*64*)].

To test the influence of postcopulatory sexual selection, we included relative combined testis mass, that is, testis mass controlled for body mass, both included as log-transformed additive covariates. Additionally, we controlled for mating system, given that the mating system could act as a confounder between ALE differences and relative testis mass. Similarly to SSD, for the relative testis mass, we have that the allometric relation between testis mass and body mass can be expressed as

$$\begin{array}{c} T=\alpha \;M_{m}^{\gamma } \end{array}$$ (15)

where *T* is testis mass. For simplicity, we use the same parameters as for SSD. The relative testis mass can then be expressed as

RT=log α=log (T)−γ log (Mm) (16)

By following the procedure for SSD, we have that

$$\begin{array}{c} \begin{array}{c} \hat{Y}=\beta \;\mathrm{RT}=\eta +\beta_{1}\;\text{log}\;\left(T\right)+\beta_{2}\;\text{log}\;\left(M_{m}\right)=\eta +\beta \left[\text{log}\;\left(T\right)-\gamma \;\text{log}\;\left(M_{m}\right)\;\right] \end{array} \end{array}$$ (17)

where, as we show in Table 1 and tables S2 and S6, in general, $\beta_{1}>0$ and $\beta_{2}<0$ , where the exemptions in some orders (e.g., artiodactyls) imply that α<1 (i.e., logα<0 ) and can be confirmed by running a regression between log-testis mass and log-body mass in these orders. Thus, we have that, as with SSD, $\beta_{1}=\beta$ and $\gamma =-\beta_{2}/\beta_{1}$ , while the effect of relative testis mass is measured by the corresponding $\beta_{1}$ . In the reproducibility code, we provide R code to demonstrate how these relationships hold on simulated data.

Life-history covariates to test the influence of the cost of reproduction included female annual productivity, calculated as the product of mean neonate or egg mass, litter or clutch size, and the mean number of litters or clutches per year per adult female (*55*). Annual productivity excluded marsupials due to their very low birth weight in the pouch. In addition, we obtained information on parental care. In mammals, parental care was categorized as either biparental (0) or female-only care (*1*), given that no species with male-only care is known. In birds, parental care was measured as the female contribution to care compared to the males, based on mean values across eight original parental care behaviors (*56*), resulting in a continuous scale in the interval [−2, 2], where −2 is male-only care and 2 is female-only care. For comparability with mammals, we transformed these scores into a categorical variable, including male care, biparental care, and female care. To evaluate robustness or our results, we tested different threshold values for defining these categories (with cutoff values at −1 and 1, at −0.5 and 0.5, or at <0 and >0), but the effect size and direction remained consistent in the BPGLS model that included age at first birth and parental care in birds (table S9, models 2 and 3). Because we found no statistical differences between the categories male care and biparental care, we ultimately combined the data into two groups (biparental/male care and female care), where values ≤0 refer to biparental/male care and values >0 refer to female care (fig. S12 and table S9, model 4). Last, to determine whether our results from populations in the wild could have been confounded by hunting, we obtained the hunting status of most populations from their corresponding references. We then carried out BPGLS on the combined effect of precopulatory sexual selection and hunting. We confirmed hunting status in 106 populations, of which 34 were hunted (15 mammals and 19 birds). As we show in table S5, the effect of hunting was negligible, while it might have potentially exacerbated the effect of the mating system.
