# Supplementary material for: Sexual selection drives sex difference in adult life expectancy across mammals and birds
Source: Sci Adv. 2025 Oct 1;11(40):eady8433. doi: 10.1126/sciadv.ady8433 (PMC12487899; doi:10.1126/sciadv.ady8433)
Supplement: Supplementary file 1 — Figs. S1 to S12 Tables S1 to S9 Legend for data S1 [file sciadv.ady8433_sm.pdf]

Science Advances
AAAS

Supplementary Materials for

Sexual selection drives sex difference in adult life expectancy across mammals and birds

Johanna Staerk *et al.*

Corresponding author: Johanna Staerk, johanna_staerk@eva.mpg.de;
Fernando Colchero, fernando_colchero@eva.mpg.de

Sci. Adv. **11**, eady8433 (2025)
DOI: 10.1126/sciadv.ady8433

This PDF file includes:

Figs. S1 to S12
Tables S1 to S9
Legend for data S1

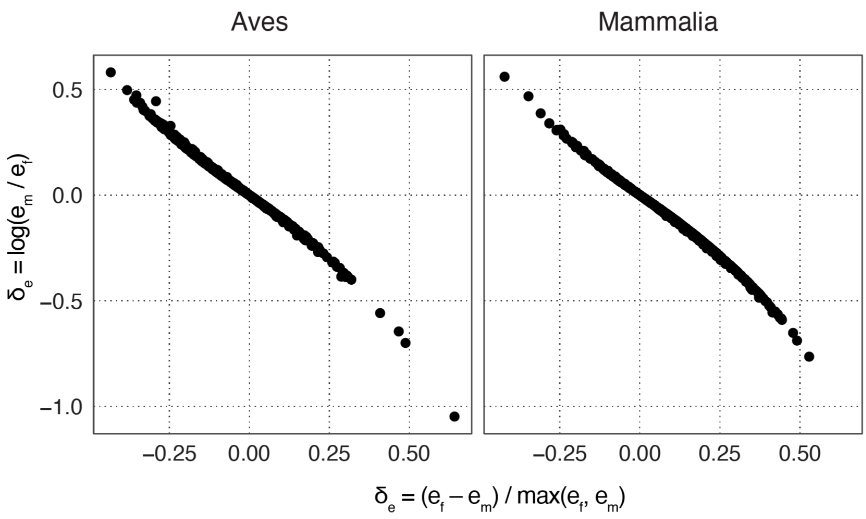

Fig. S1. Comparison of Adult Life Expectancy differences (δ_e) measures. The measure used in this study (on x-axis) is compared with a commonly used metric (on y-axis). e_m denotes male adult life expectancy and e_f denotes female adult life expectancy. Although this measure should produce a one-to-one mapping, the small amount of heaping is caused by the fact that the measures are calculated as posterior means.

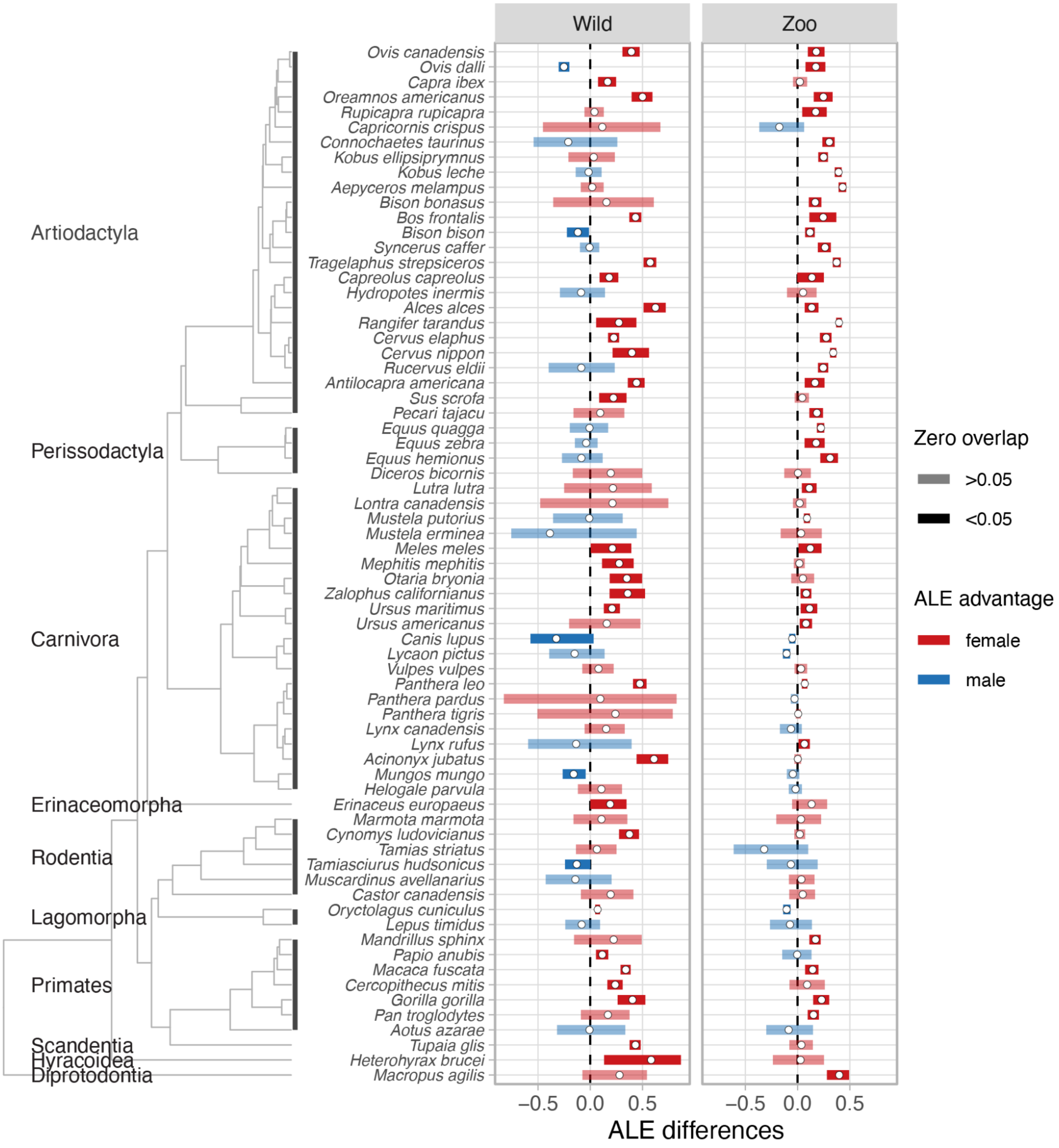

Fig. S2. ALE differences in wild and zoo populations for mammals. Red and blue bars show the 95% credible intervals, while the white dots show the posterior mean ALE difference.

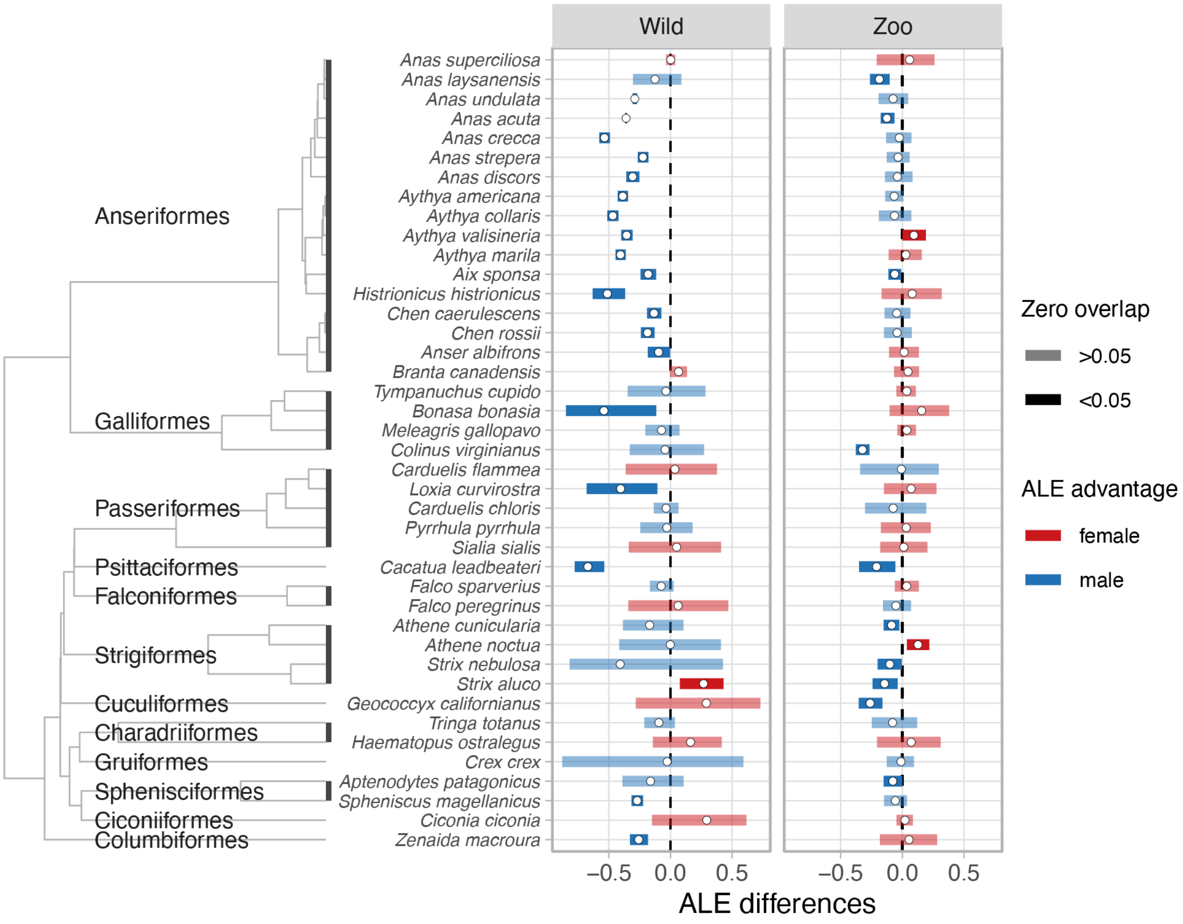

Fig. S3. ALE differences in wild and zoo populations for birds. Red and blue bars show the 95% credible intervals, while the white dots show the posterior mean ALE difference.

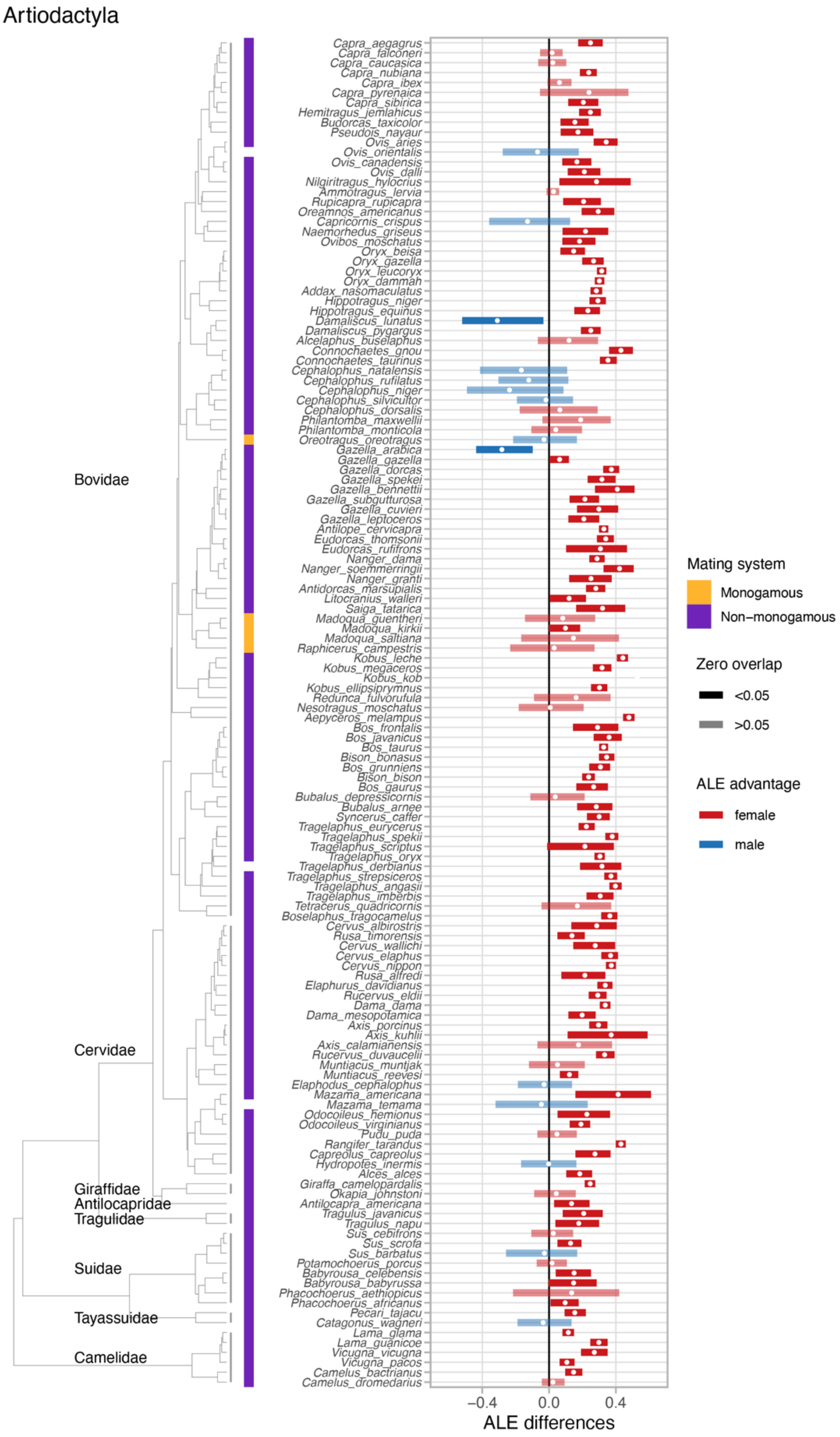

Fig. S4A ALE differences for the order Artiodactyla. Red and blue bars show the 95% credible intervals, while the white dots show the posterior mean ALE difference.

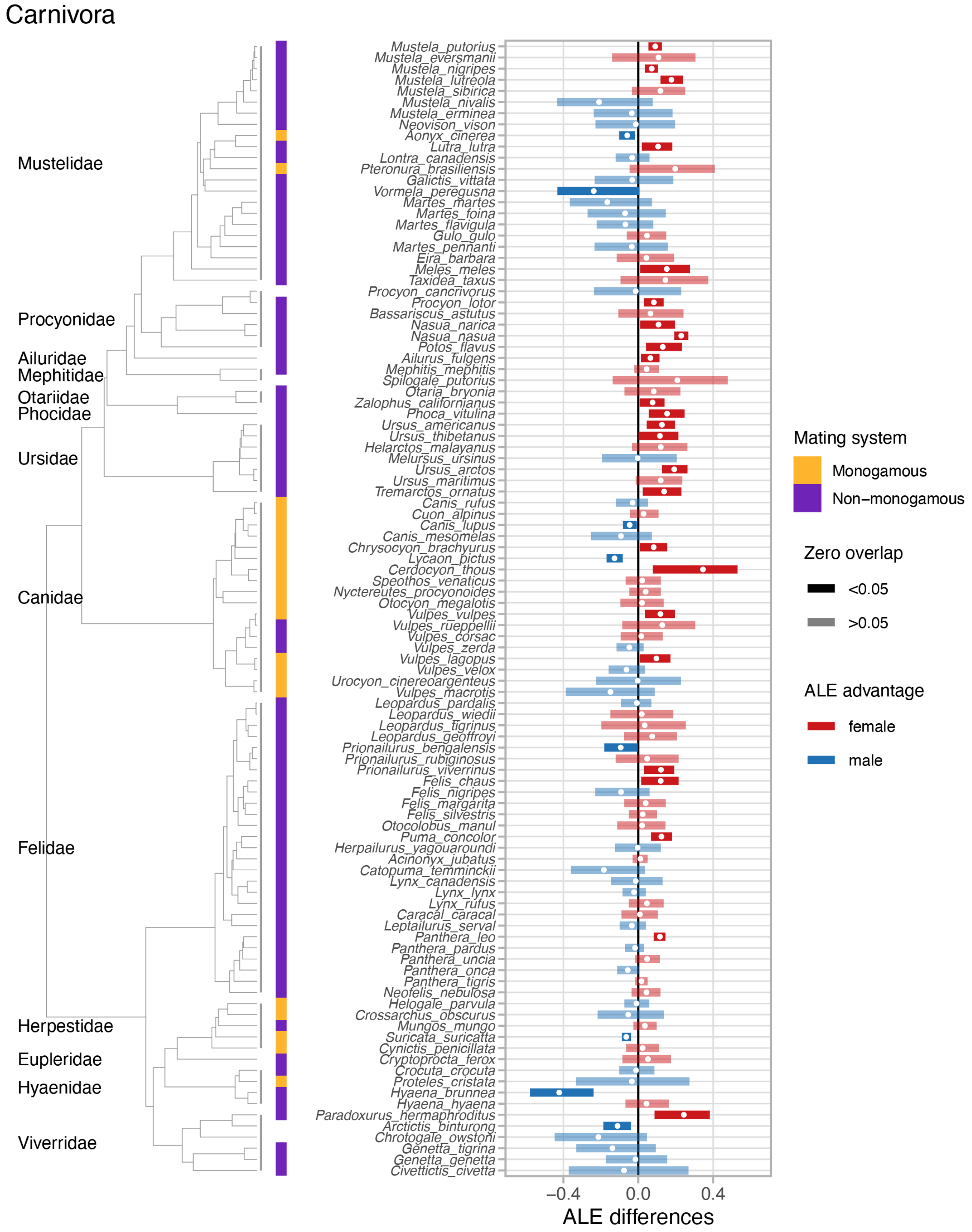

Fig. S4B ALE differences for the order Carnivora. Red and blue bars show the 95% credible intervals, while the white dots show the posterior mean ALE difference.

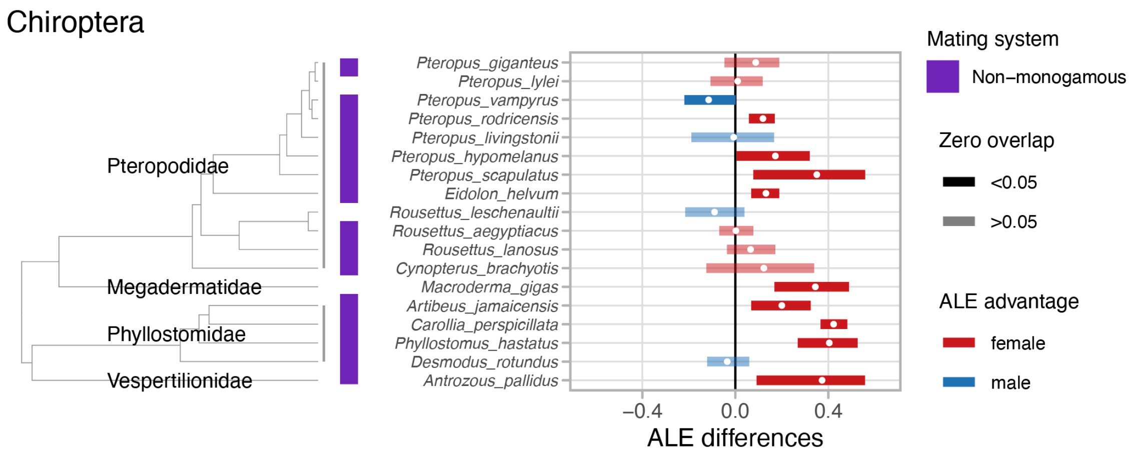

Fig. S4C ALE differences for the order Chiroptera. Red and blue bars show the 95% credible intervals, while the white dots show the posterior mean ALE difference.

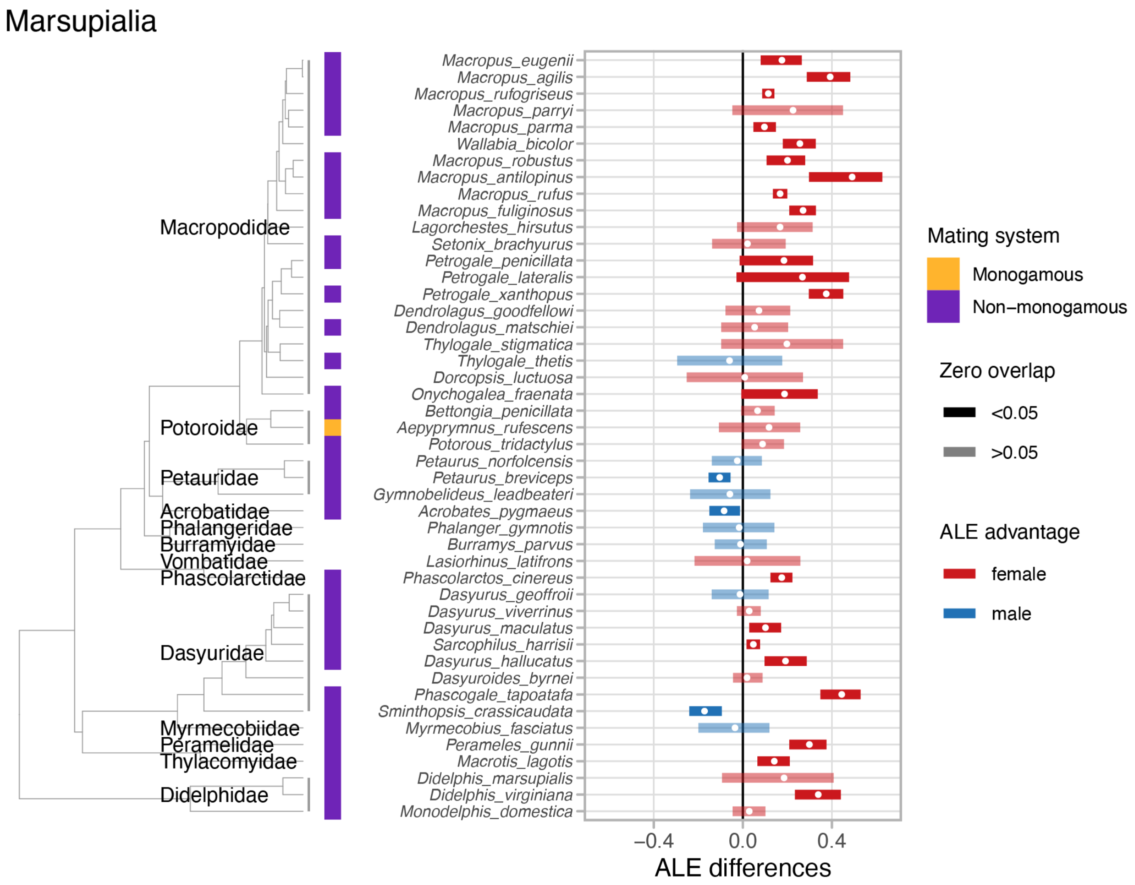

Fig. S4D ALE differences for Marsupialia. Red and blue bars show the 95% credible intervals, while the white dots show the posterior mean ALE difference.

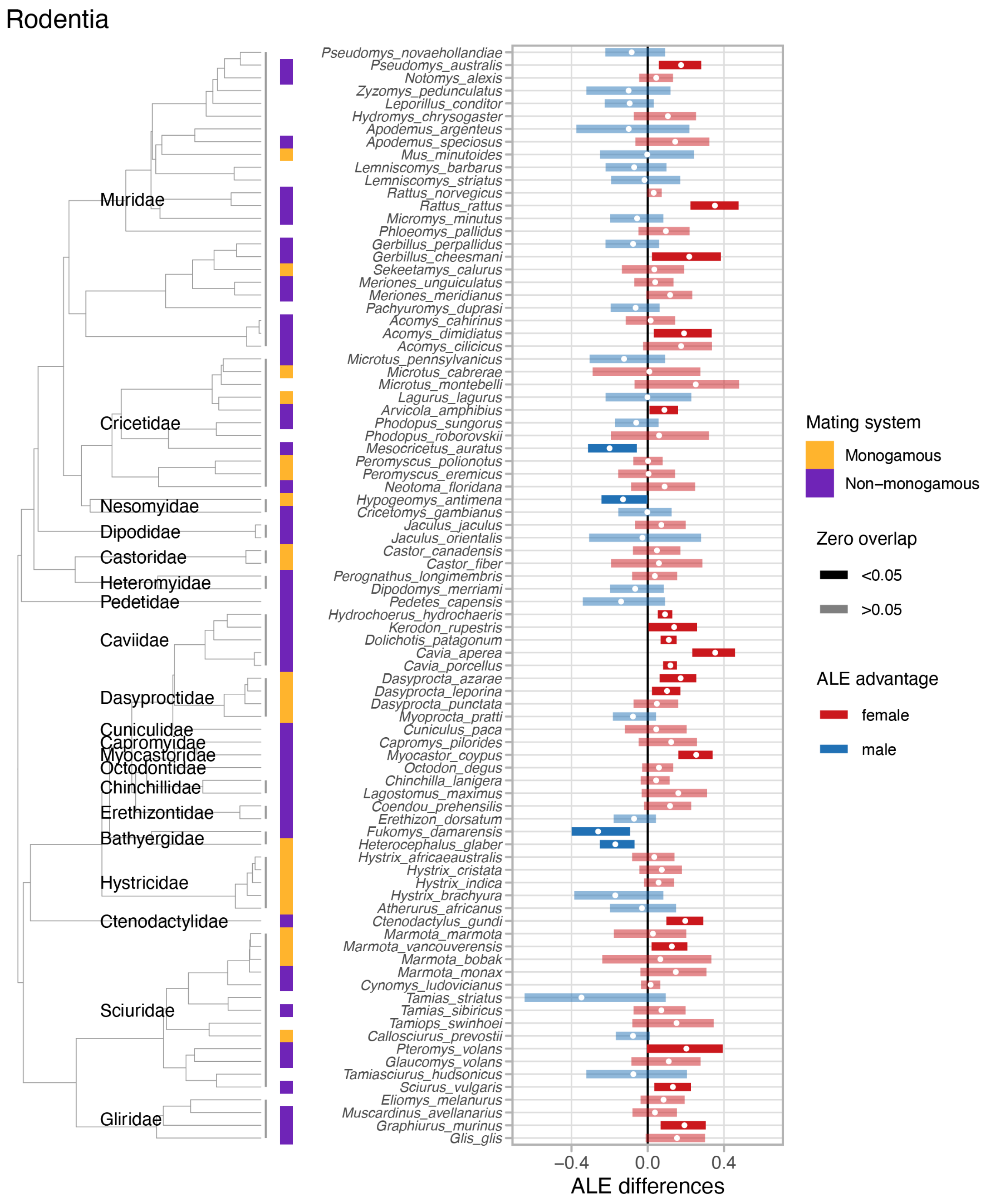

Fig. S4E ALE differences for the order Rodentia. Red and blue bars show the 95% credible intervals, while the white dots show the posterior mean ALE difference.

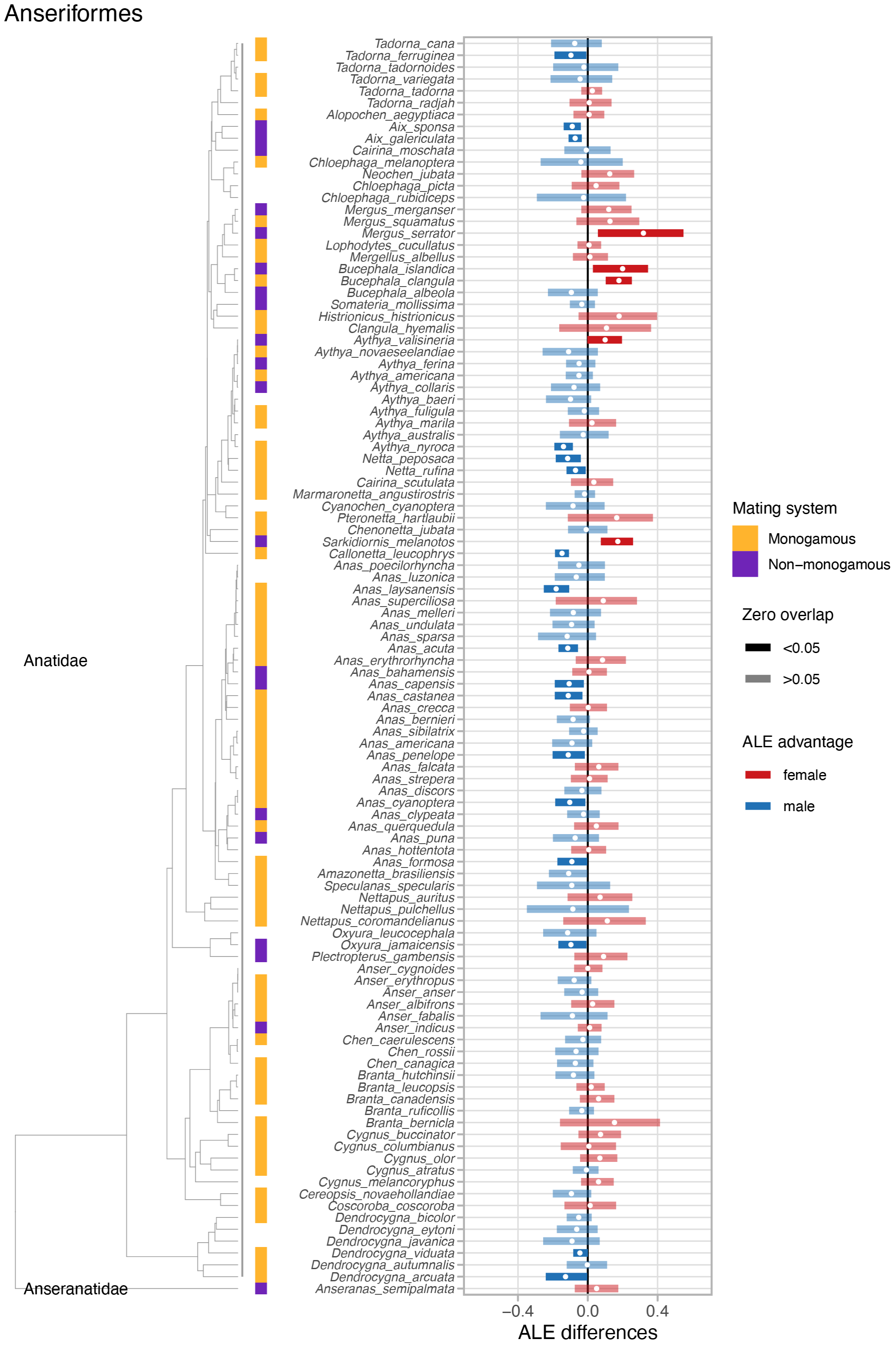

Fig. S4F ALE differences for the order Anseriformes. Red and blue bars show the 95% credible intervals, while the white dots show the posterior mean ALE difference.

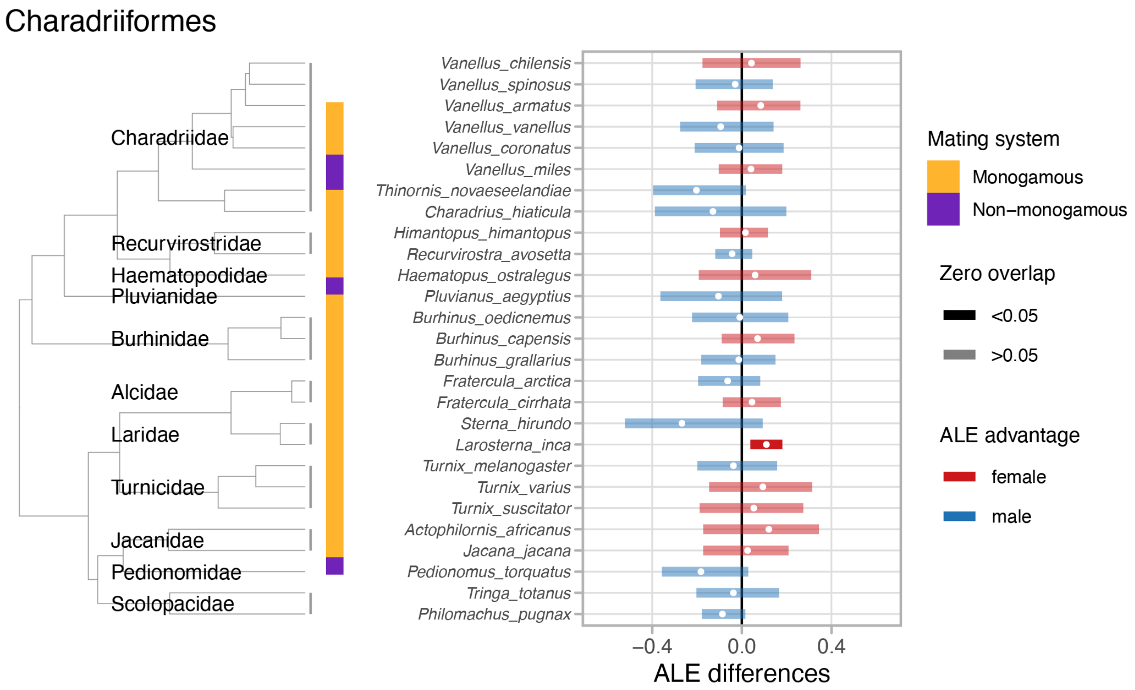

Fig. S4G ALE differences for the order Charadriiformes. Red and blue bars show the 95% credible intervals, while the white dots show the posterior mean ALE difference.

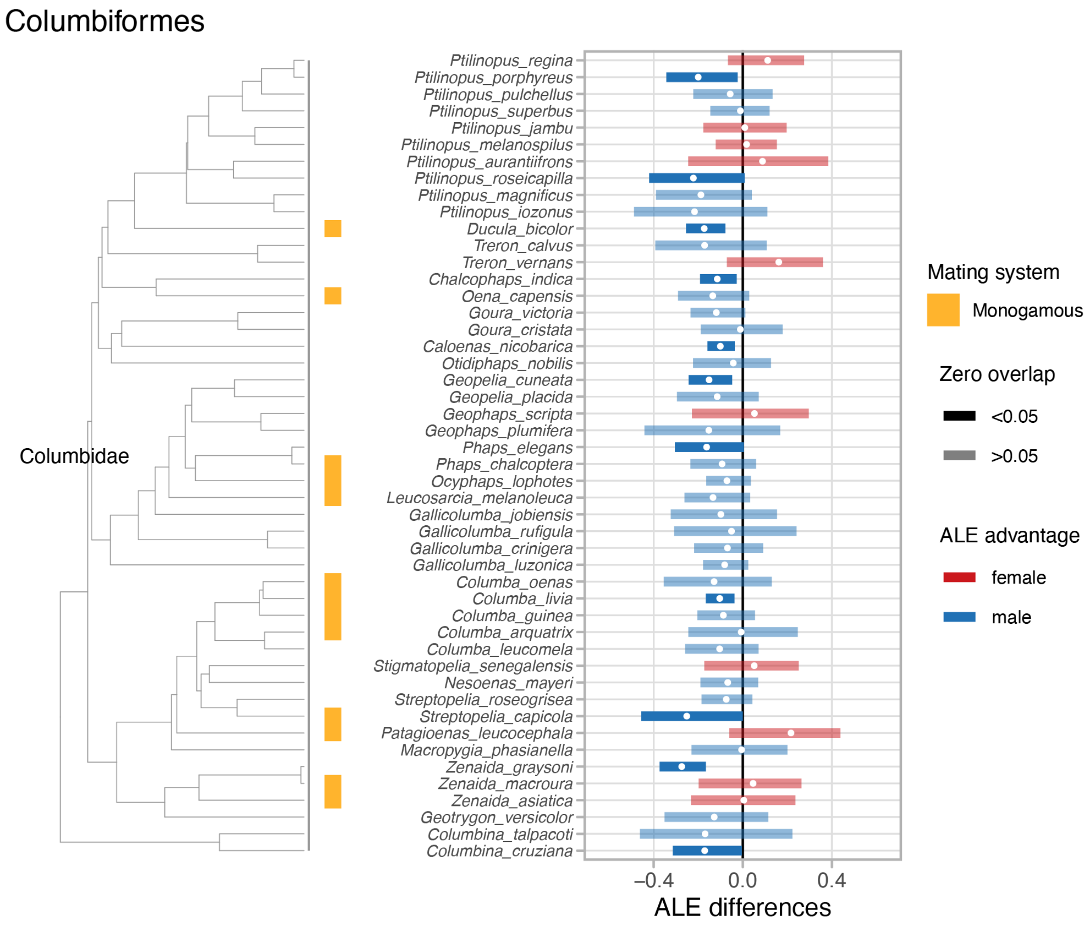

Fig. S4H ALE differences for the order Columbiformes. Red and blue bars show the 95% credible intervals, while the white dots show the posterior mean ALE difference.

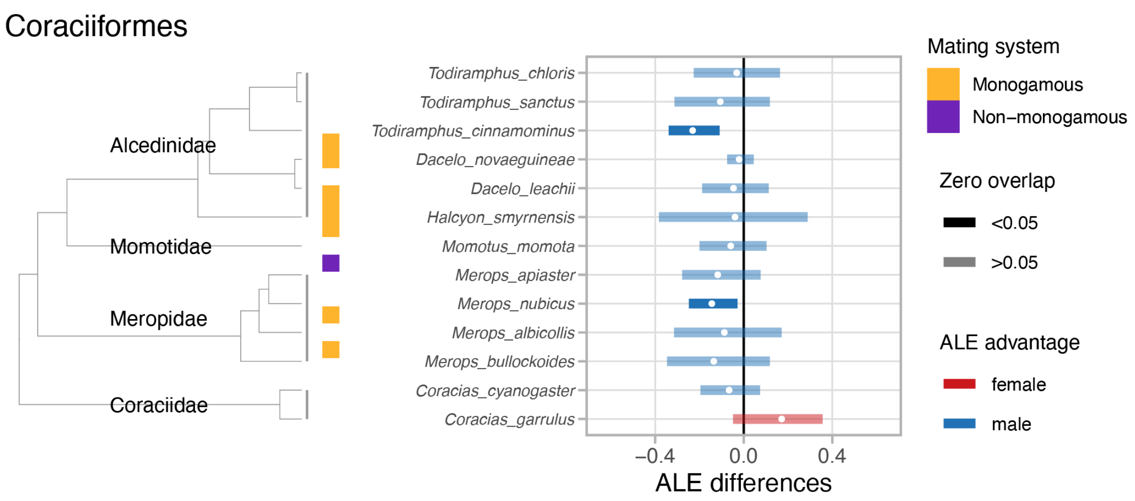

Fig. S4I ALE differences for the order Coraciiformes. Red and blue bars show the 95% credible intervals, while the white dots show the posterior mean ALE difference.

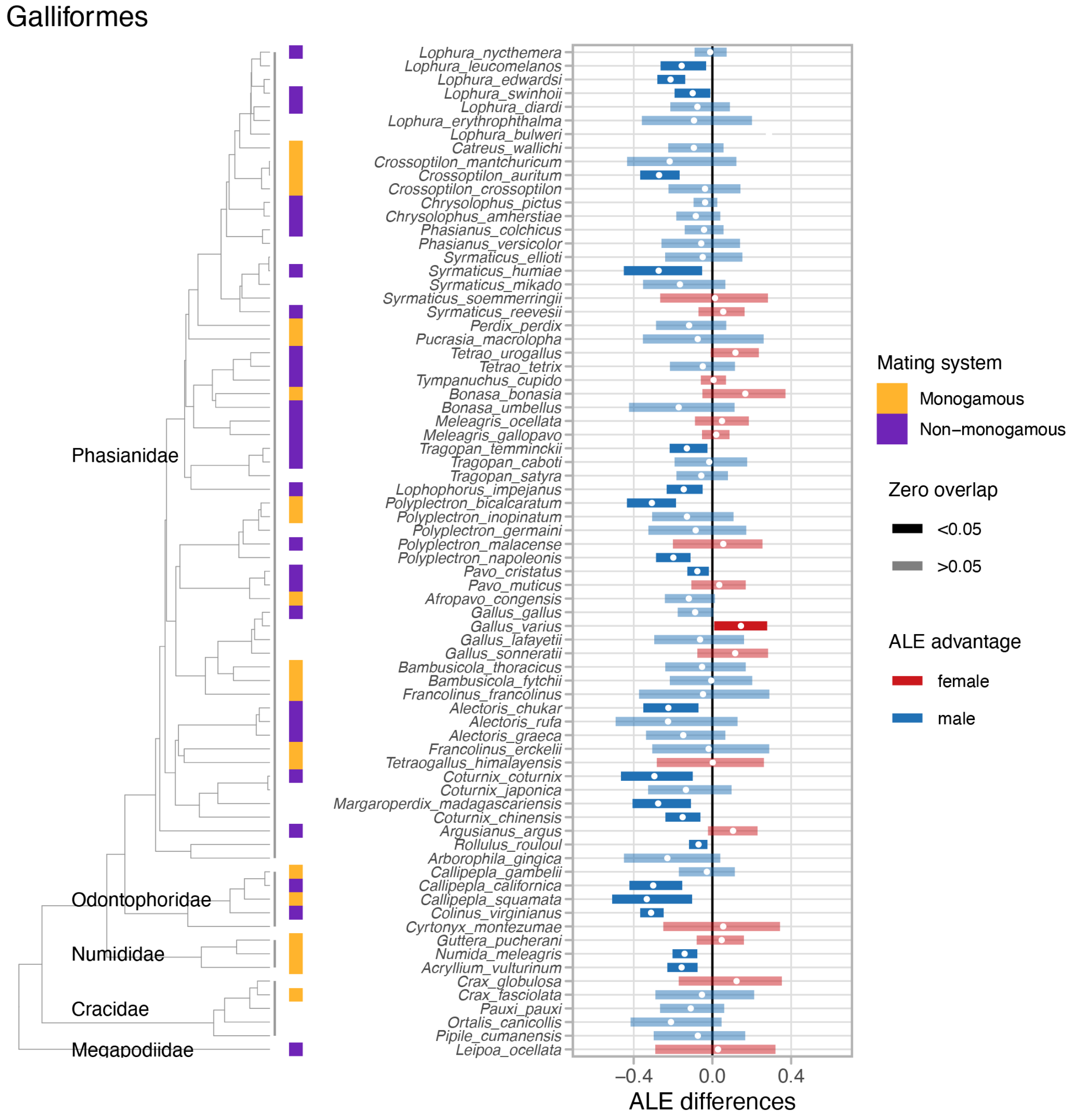

Fig. S4J ALE differences for the order Galliformes. Red and blue bars show the 95% credible intervals, while the white dots show the posterior mean ALE difference.

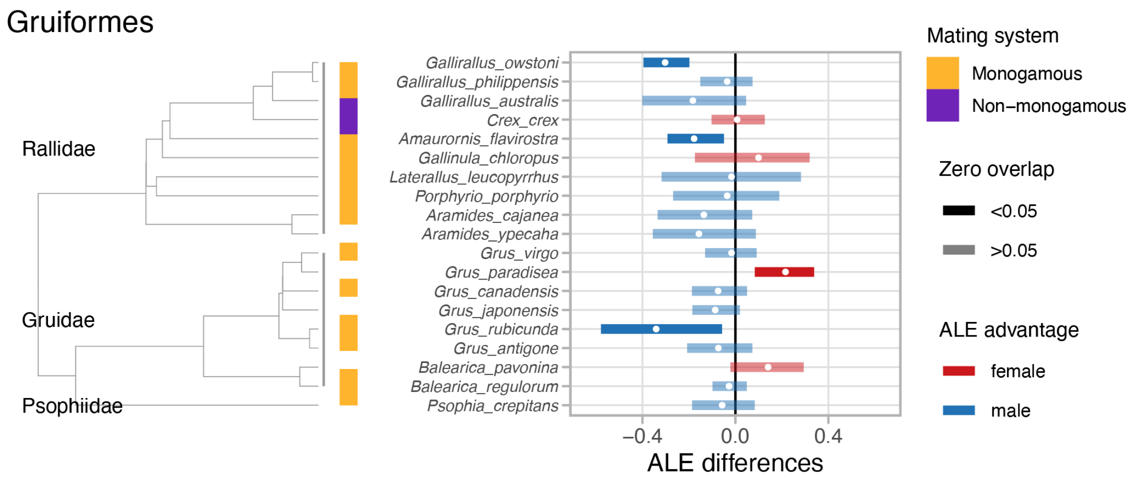

Fig. S4K ALE differences for the order Gruiformes. Red and blue bars show the 95% credible intervals, while the white dots show the posterior mean ALE difference.

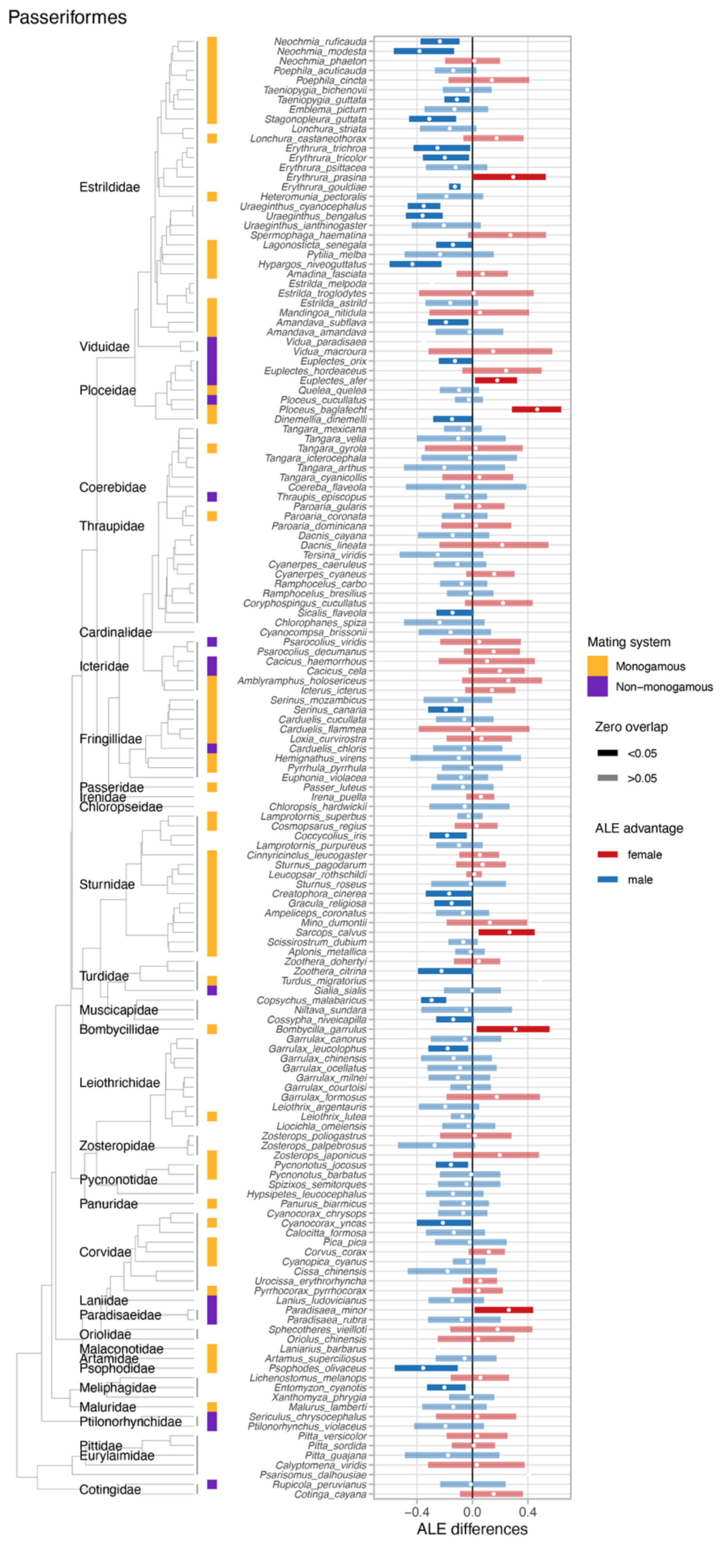

Fig. S4L ALE differences for the order Passeriformes. Red and blue bars show the 95% credible intervals, while the white dots show the posterior mean ALE difference.

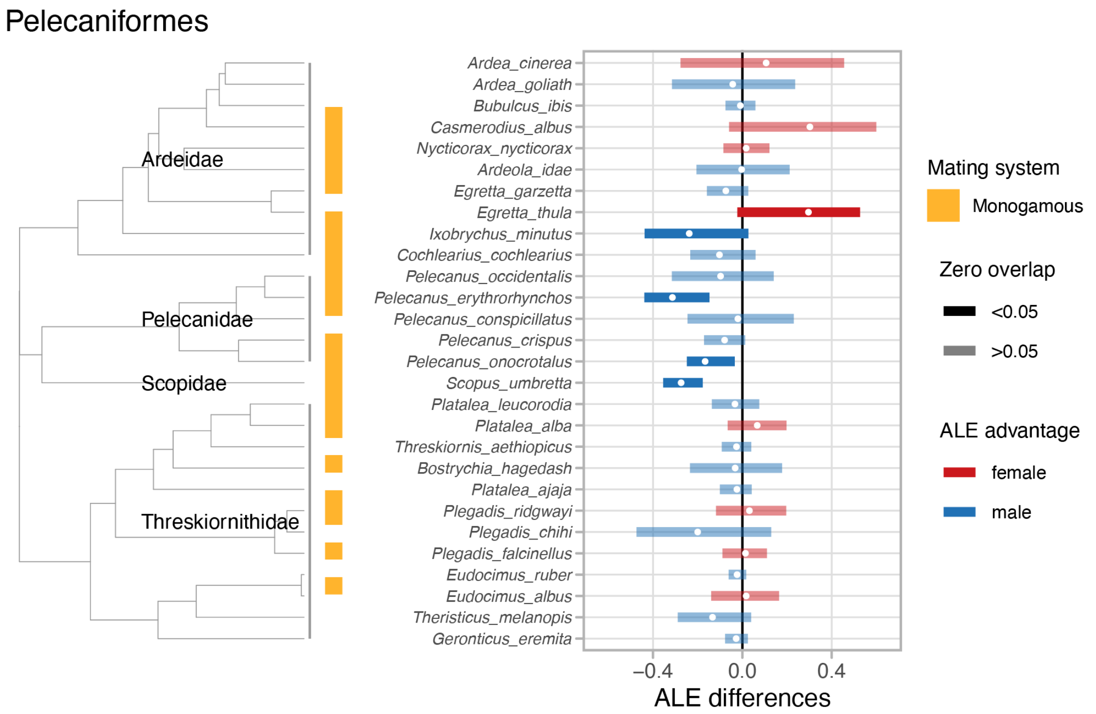

Fig. S4M ALE differences for the order Pelecaniformes. Red and blue bars show the 95% credible intervals, while the white dots show the posterior mean ALE difference.

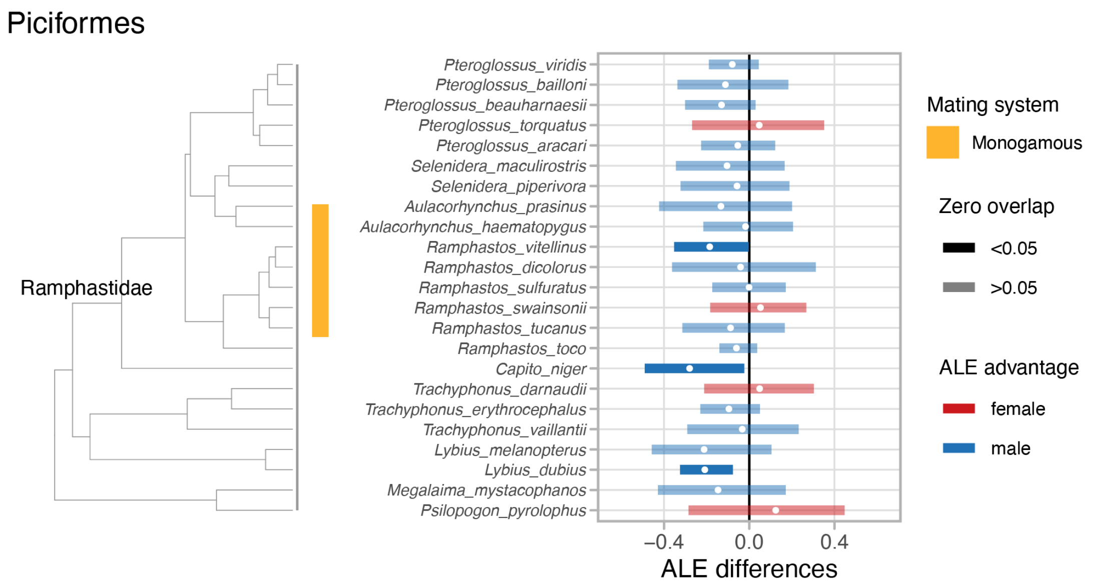

Fig. S4N ALE differences for the order Piciformes. Red and blue bars show the 95% credible intervals, while the white dots show the posterior mean ALE difference.

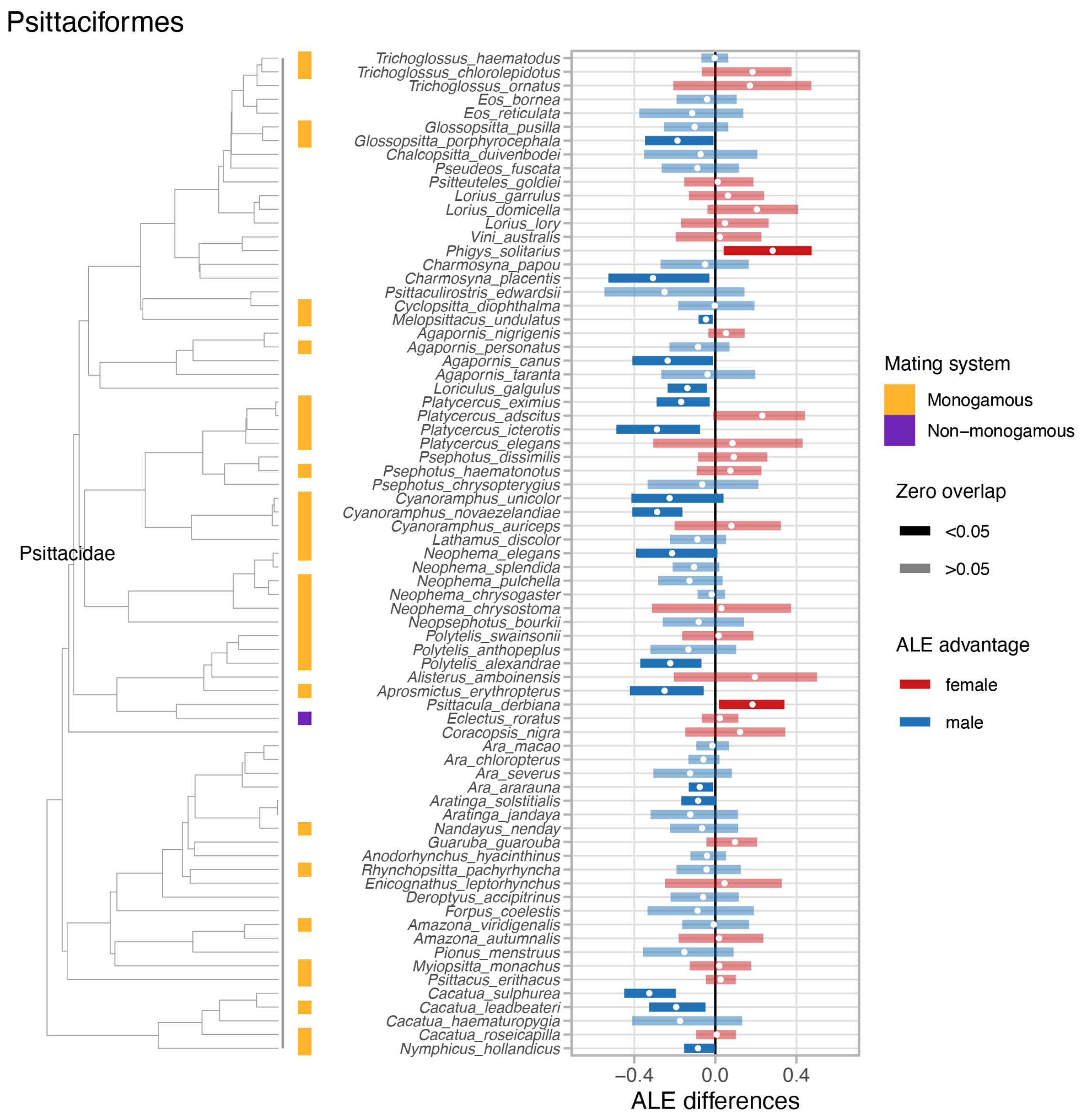

Fig. S4O ALE differences for the order Psittaciformes. Red and blue bars show the 95% credible intervals, while the white dots show the posterior mean ALE difference.

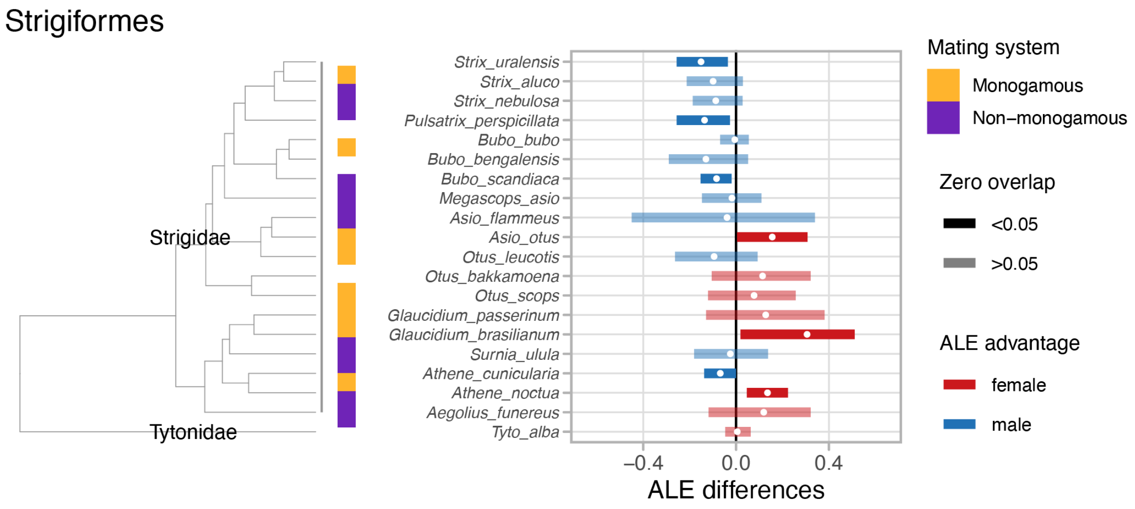

Fig. S4P ALE differences for the order Strigiformes. Red and blue bars show the 95% credible intervals, while the white dots show the posterior mean ALE difference.

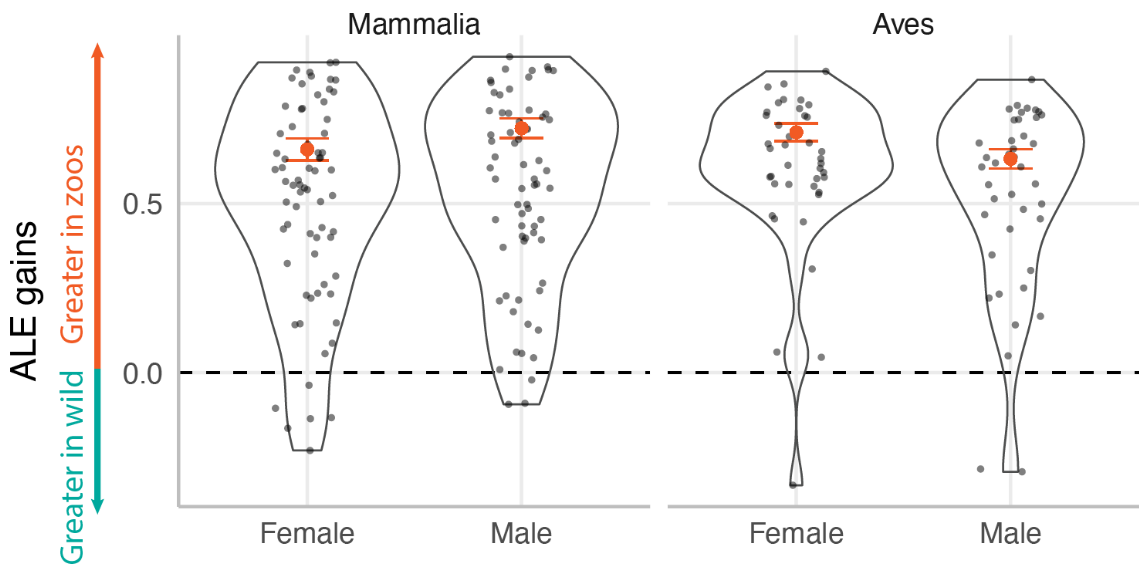

Fig. S5. Adult life expectancy differences (ALE gains) in wild and zoo environments per sex for mammals and birds. Orange points show the weighted mean ALE gains, and error bars show weighted standard errors.

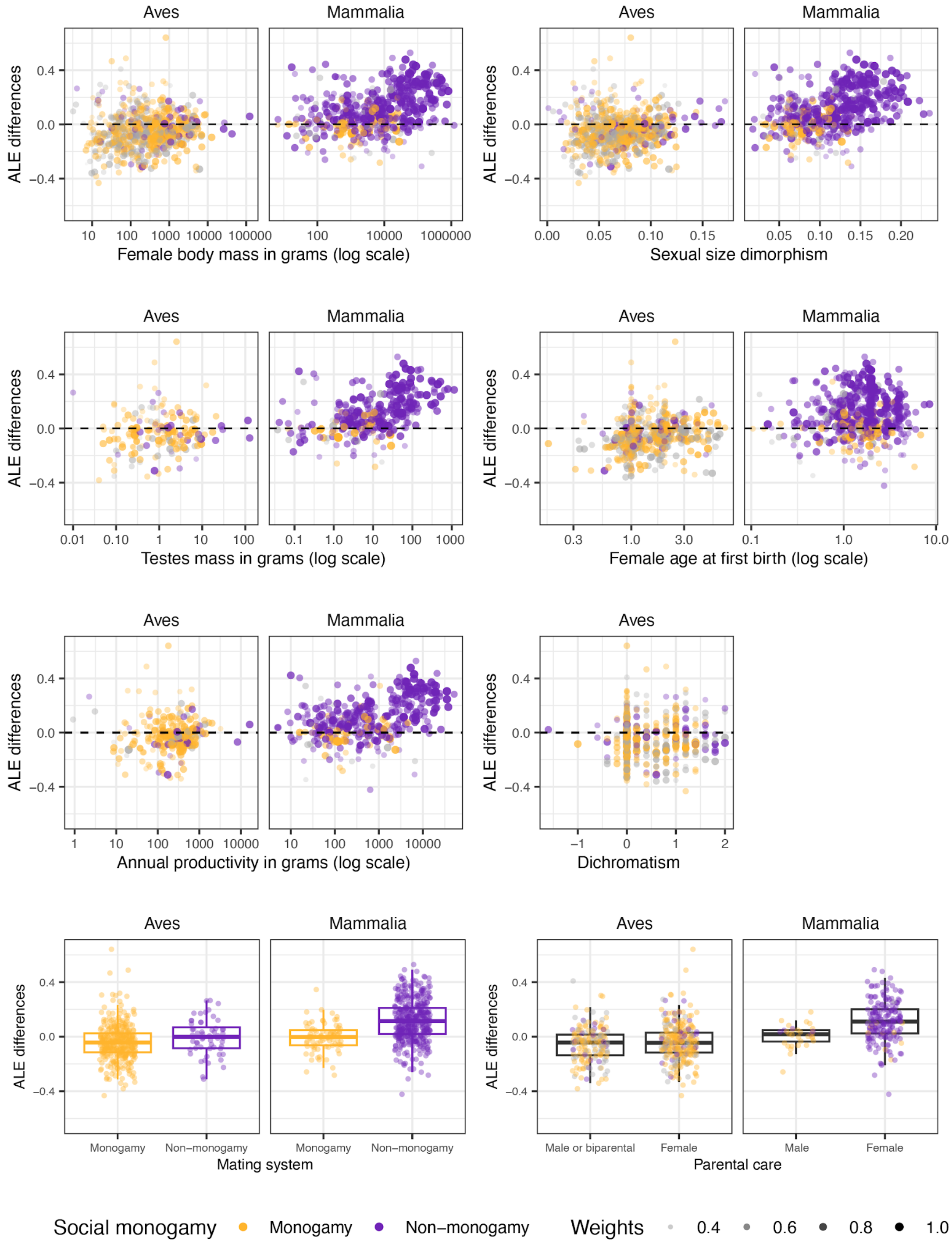

Fig. S6. Life History traits vs Adult Life Expectancy difference for mammals and birds. Weights refer to PGLS weights, whic h are shown in all plots except the boxplots.

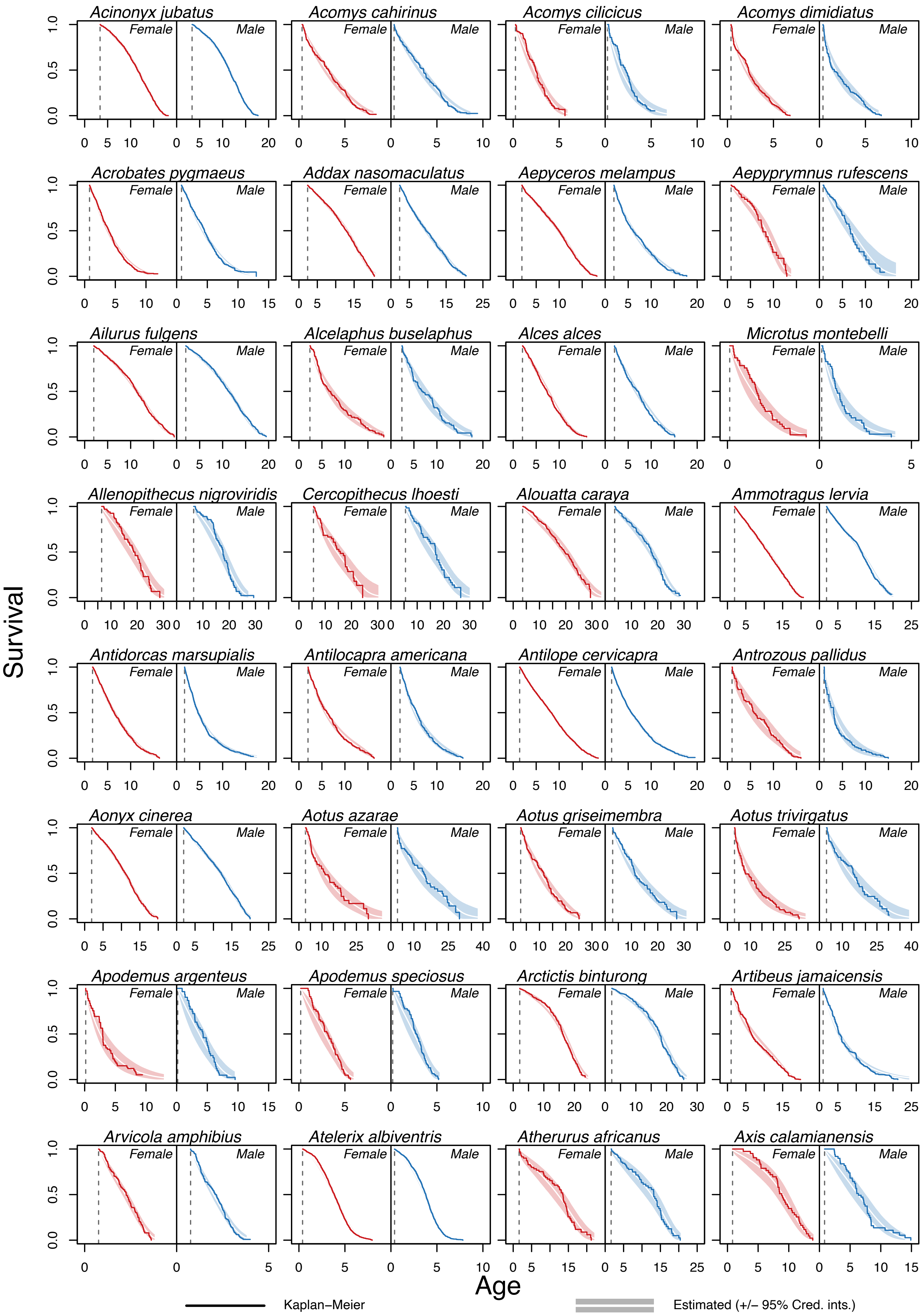

Fig. S7A. Goodness of fit plots for females and males from the Bayesian survival trajectory analysis (BaSTA) for mammals.

The red and blue polygons show the estimated survival from BaSTA with the 95% credible intervals and the dark lines are the Kaplan–Meier survival curves from the data.

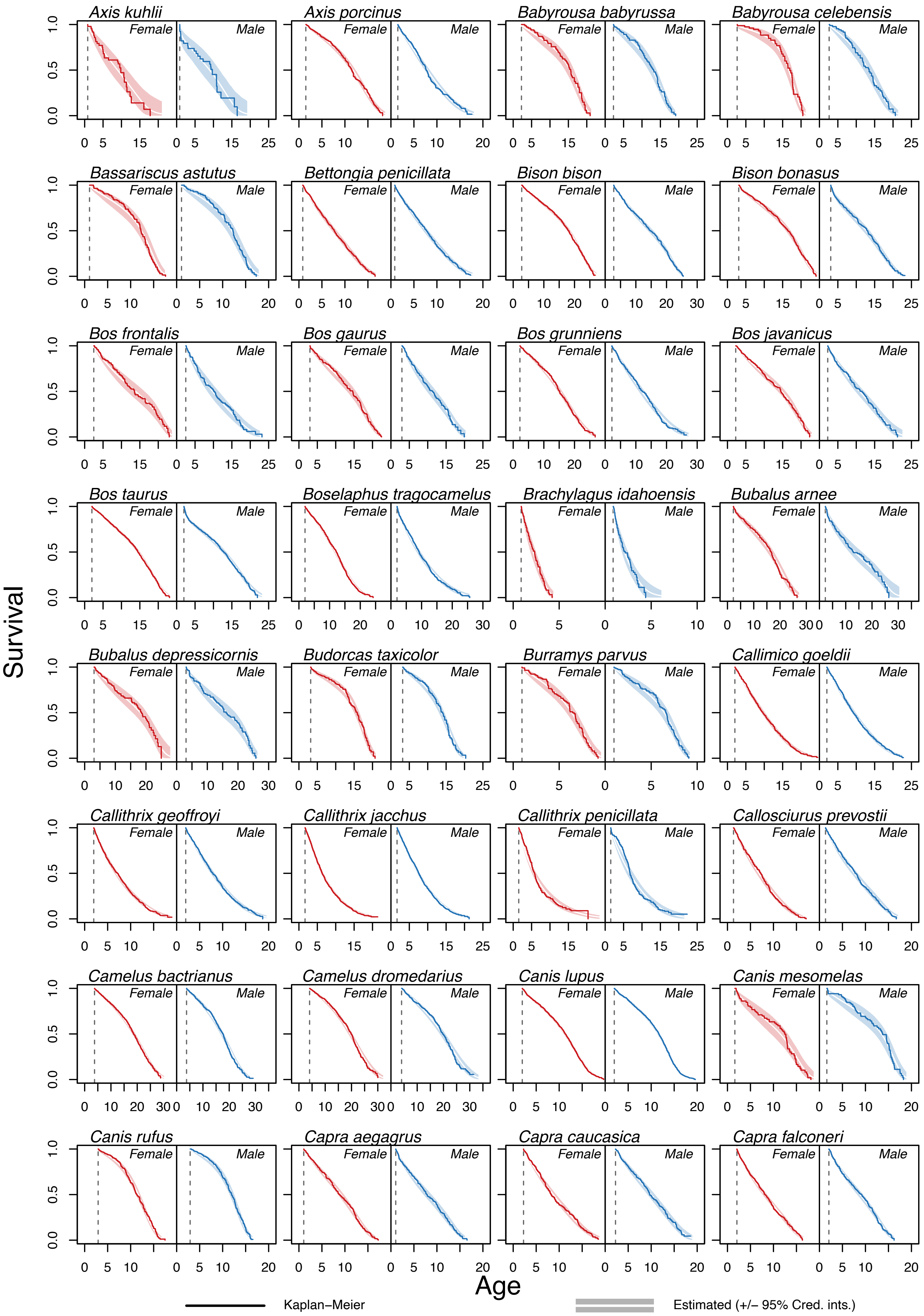

Fig. S7B. Goodness of fit plots for females and males from the Bayesian survival trajectory analysis (BaSTA) for mammals.

The red and blue polygons show the estimated survival from BaSTA with the 95% credible intervals and the dark lines are the Kaplan–Meier survival curves from the data.

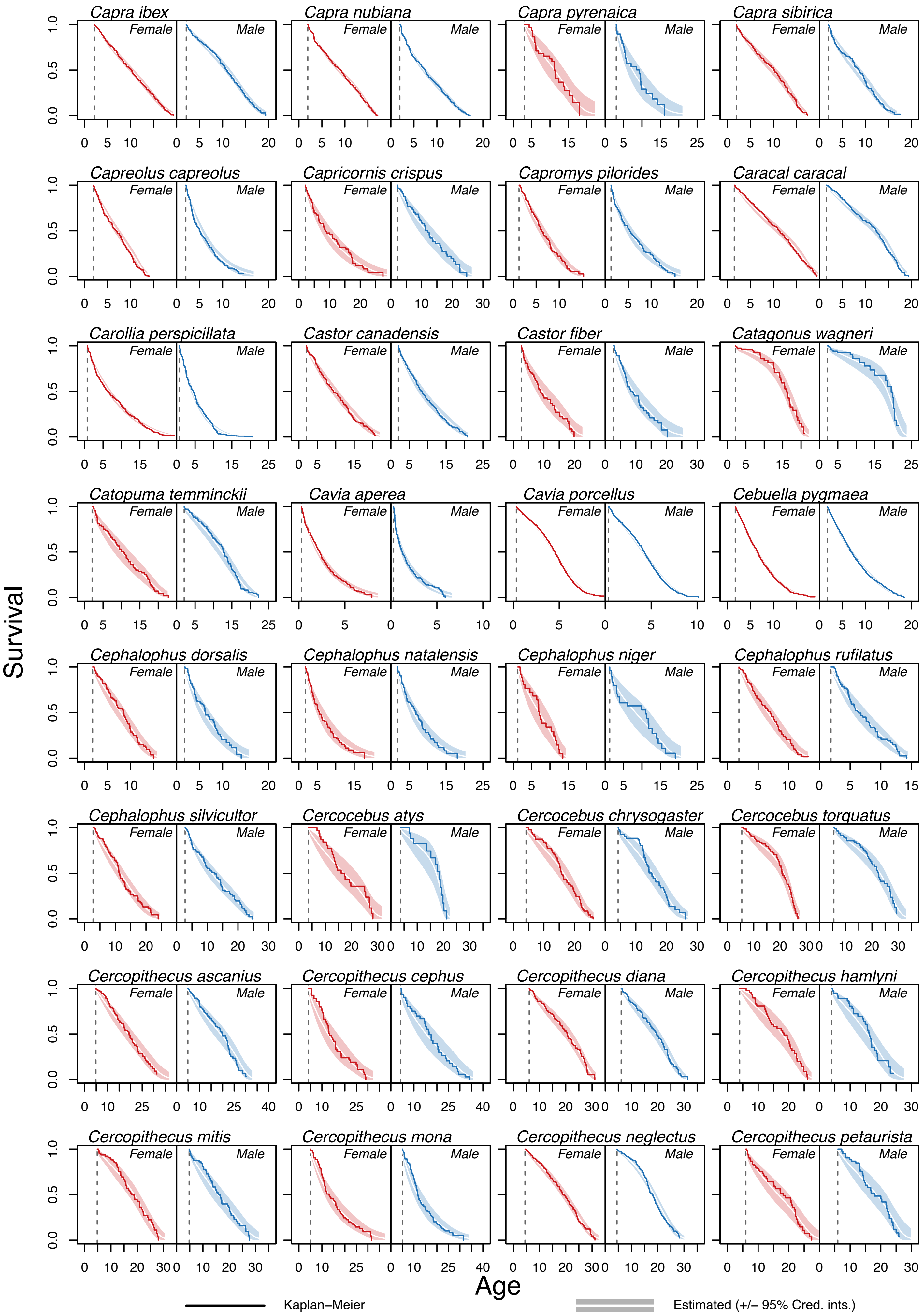

Fig. S7C. Goodness of fit plots for females and males from the Bayesian survival trajectory analysis (BaSTA) for mammals.

The red and blue polygons show the estimated survival from BaSTA with the 95% credible intervals and the dark lines are the Kaplan−Meier survival curves from the data.

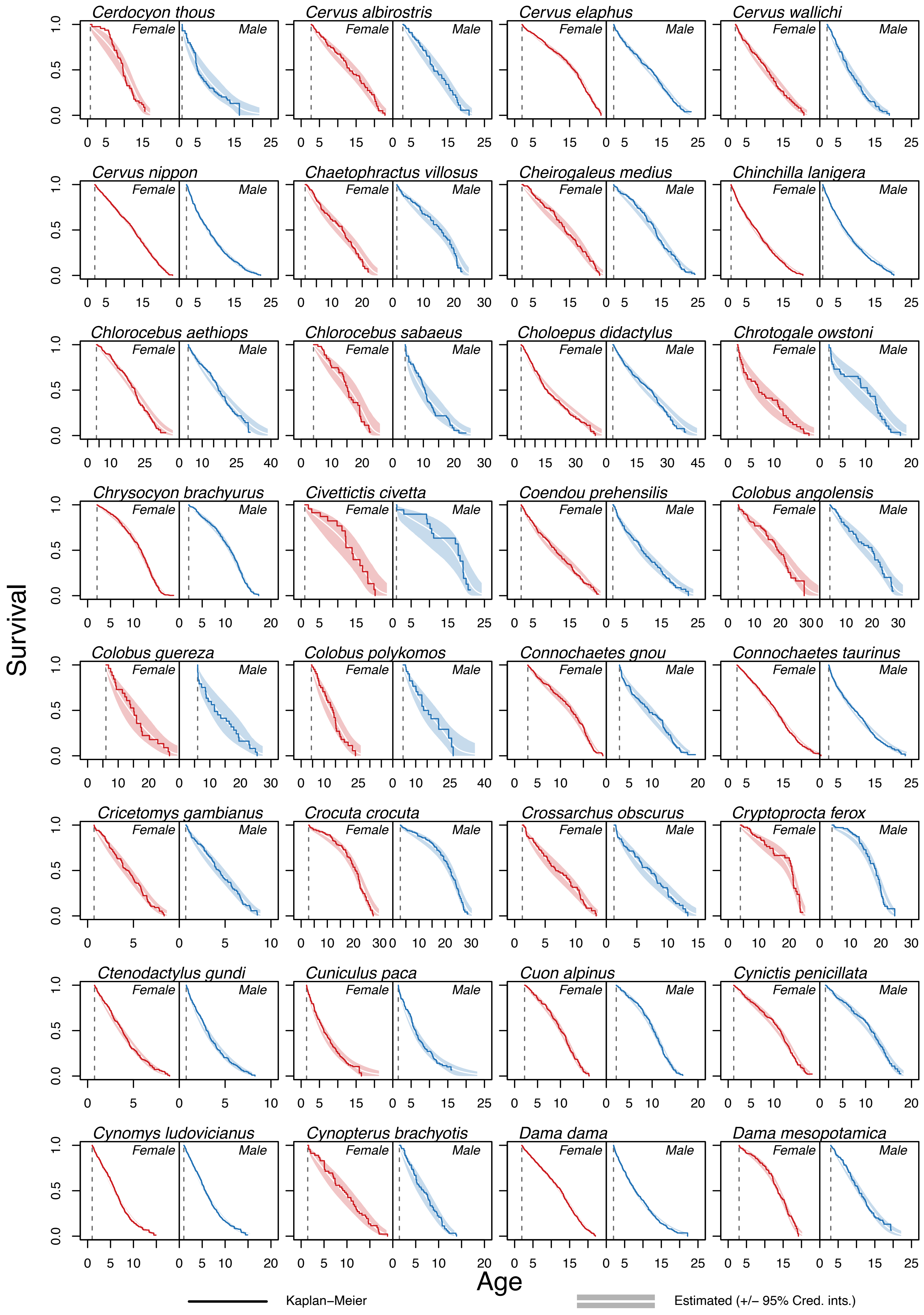

Fig. S7D. Goodness of fit plots for females and males from the Bayesian survival trajectory analysis (BaSTA) for mammals.

The red and blue polygons show the estimated survival from BaSTA with the 95% credible intervals and the dark lines are the Kaplan–Meier survival curves from the data.

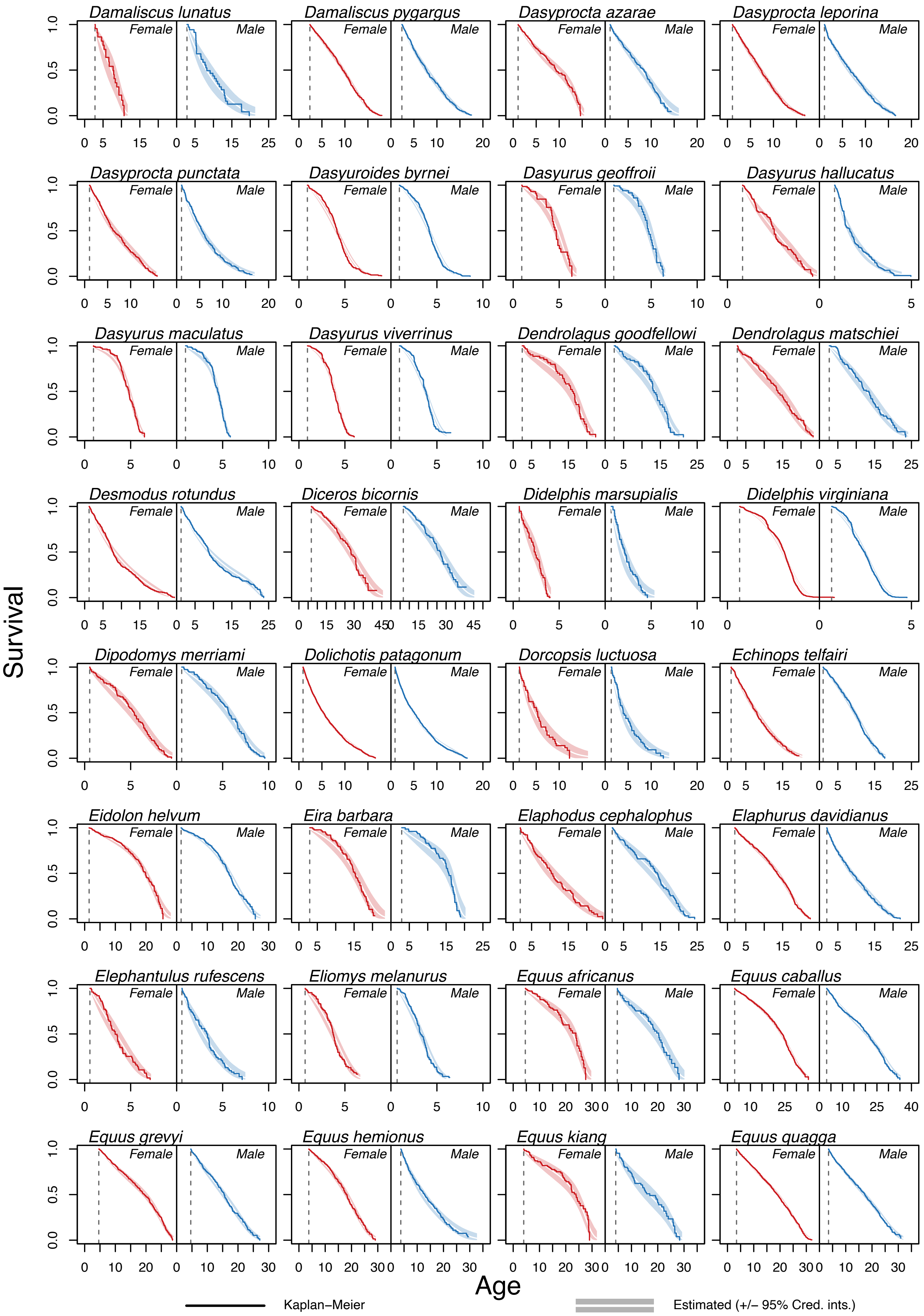

Fig. S7E. Goodness of fit plots for females and males from the Bayesian survival trajectory analysis (BaSTA) for mammals.

The red and blue polygons show the estimated survival from BaSTA with the 95% credible intervals and the dark lines are the Kaplan–Meier survival curves from the data.

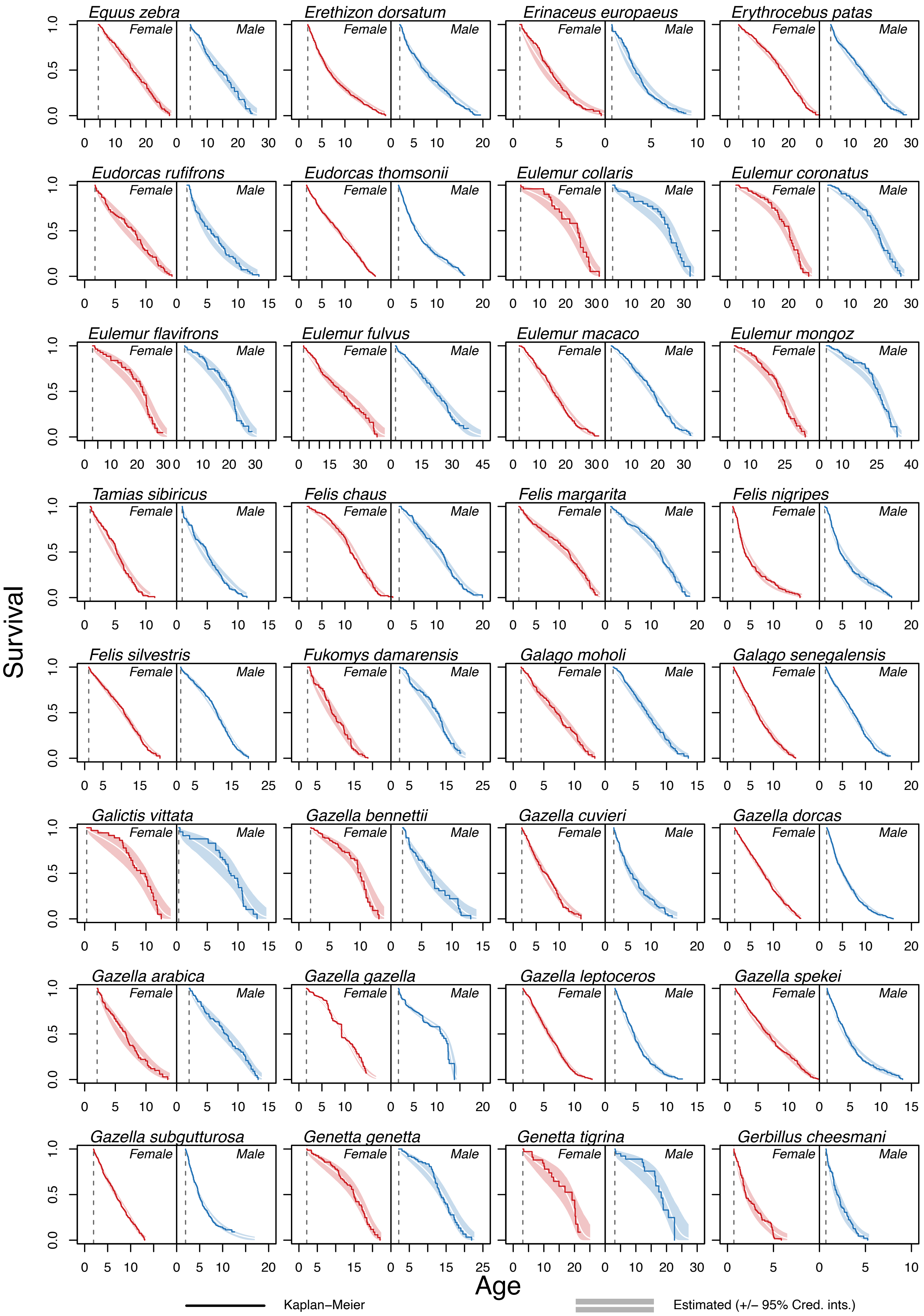

Fig. S7F. Goodness of fit plots for females and males from the Bayesian survival trajectory analysis (BaSTA) for mammals.

The red and blue polygons show the estimated survival from BaSTA with the 95% credible intervals and the dark lines are the Kaplan–Meier survival curves from the data.

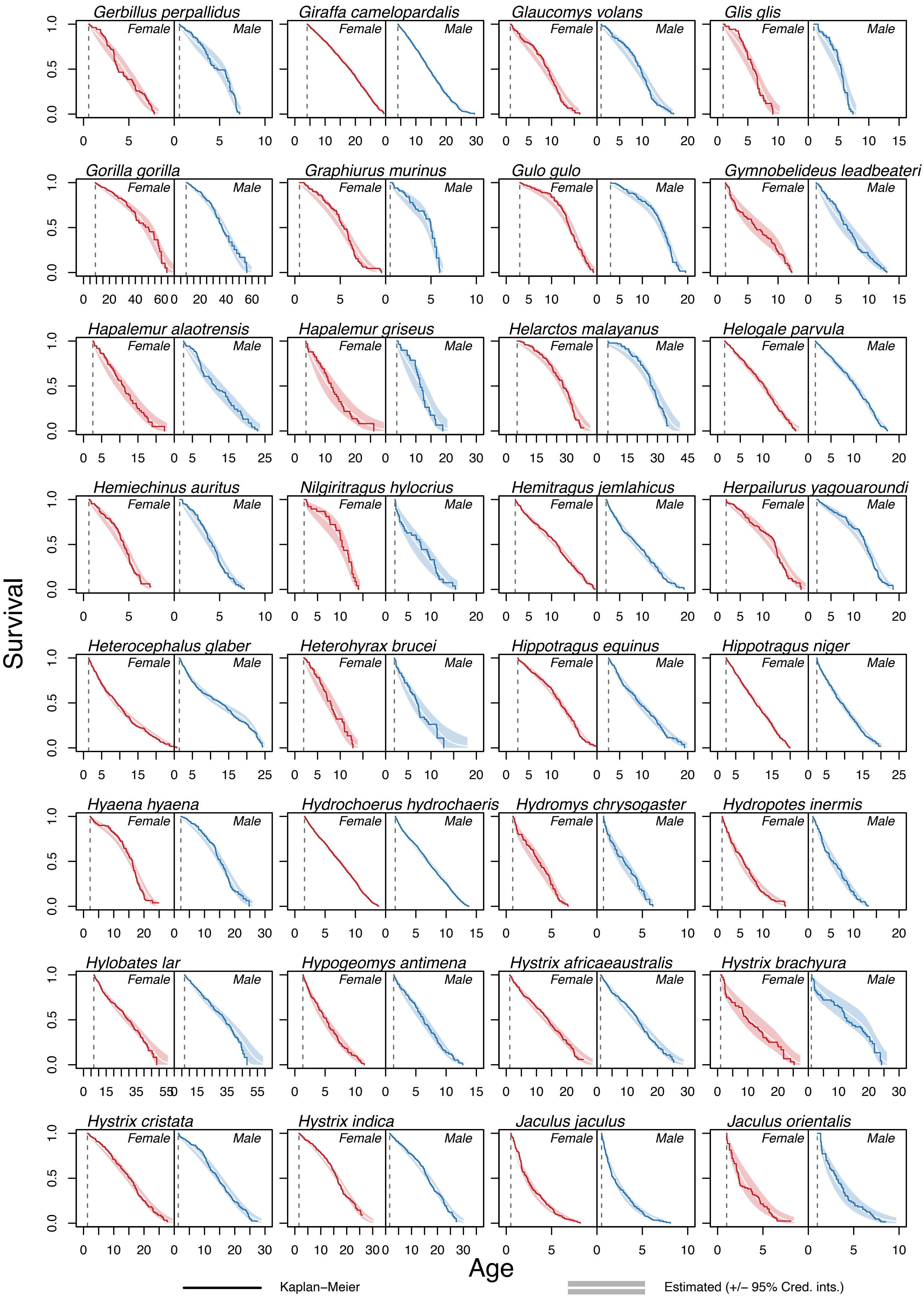

Fig. S7G. Goodness of fit plots for females and males from the Bayesian survival trajectory analysis (BaSTA) for mammals.

The red and blue polygons show the estimated survival from BaSTA with the 95% credible intervals and the dark lines are the Kaplan−Meier survival curves from the data.

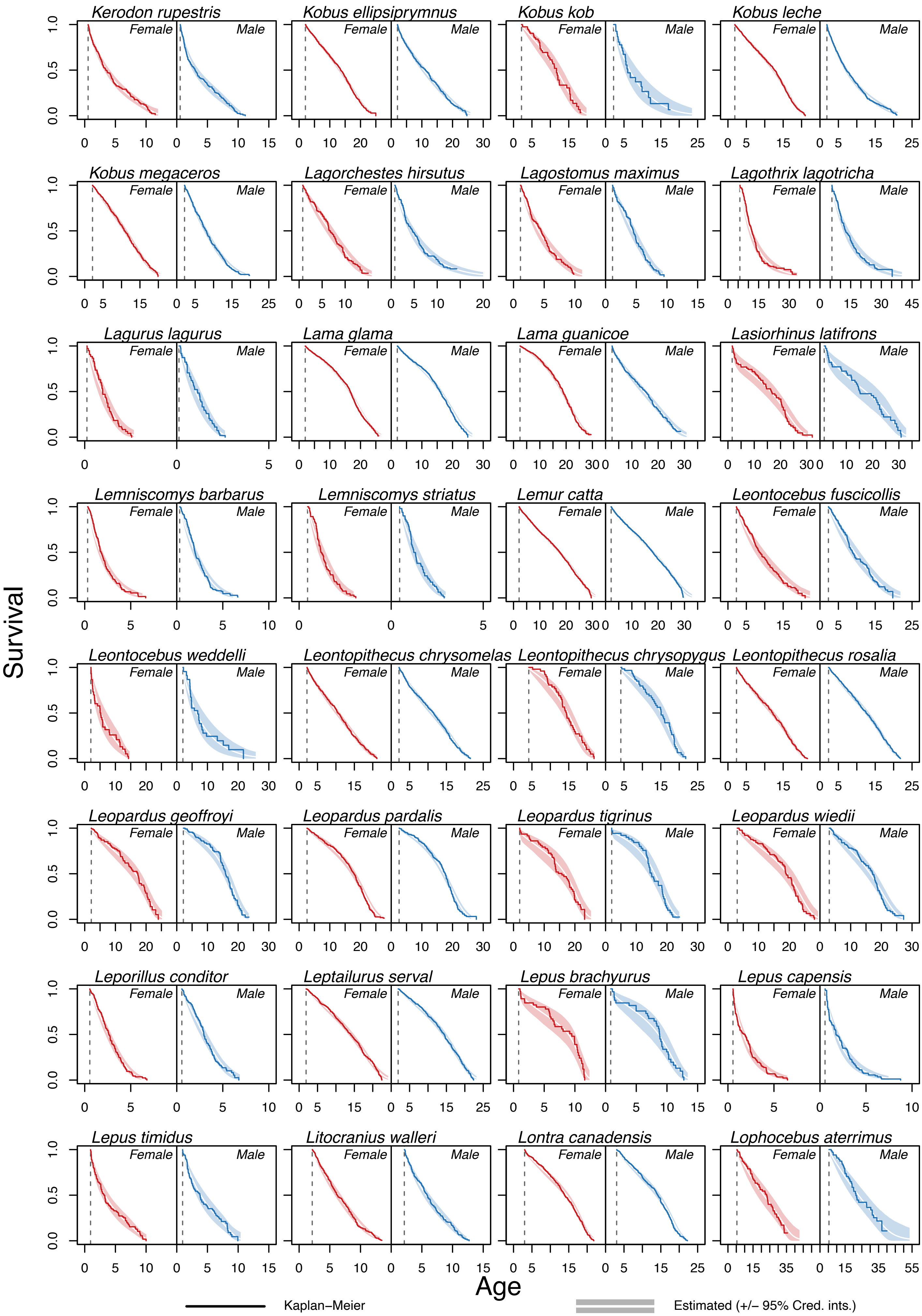

Fig. S7H. Goodness of fit plots for females and males from the Bayesian survival trajectory analysis (BaSTA) for mammals.

The red and blue polygons show the estimated survival from BaSTA with the 95% credible intervals and the dark lines are the Kaplan–Meier survival curves from the data.

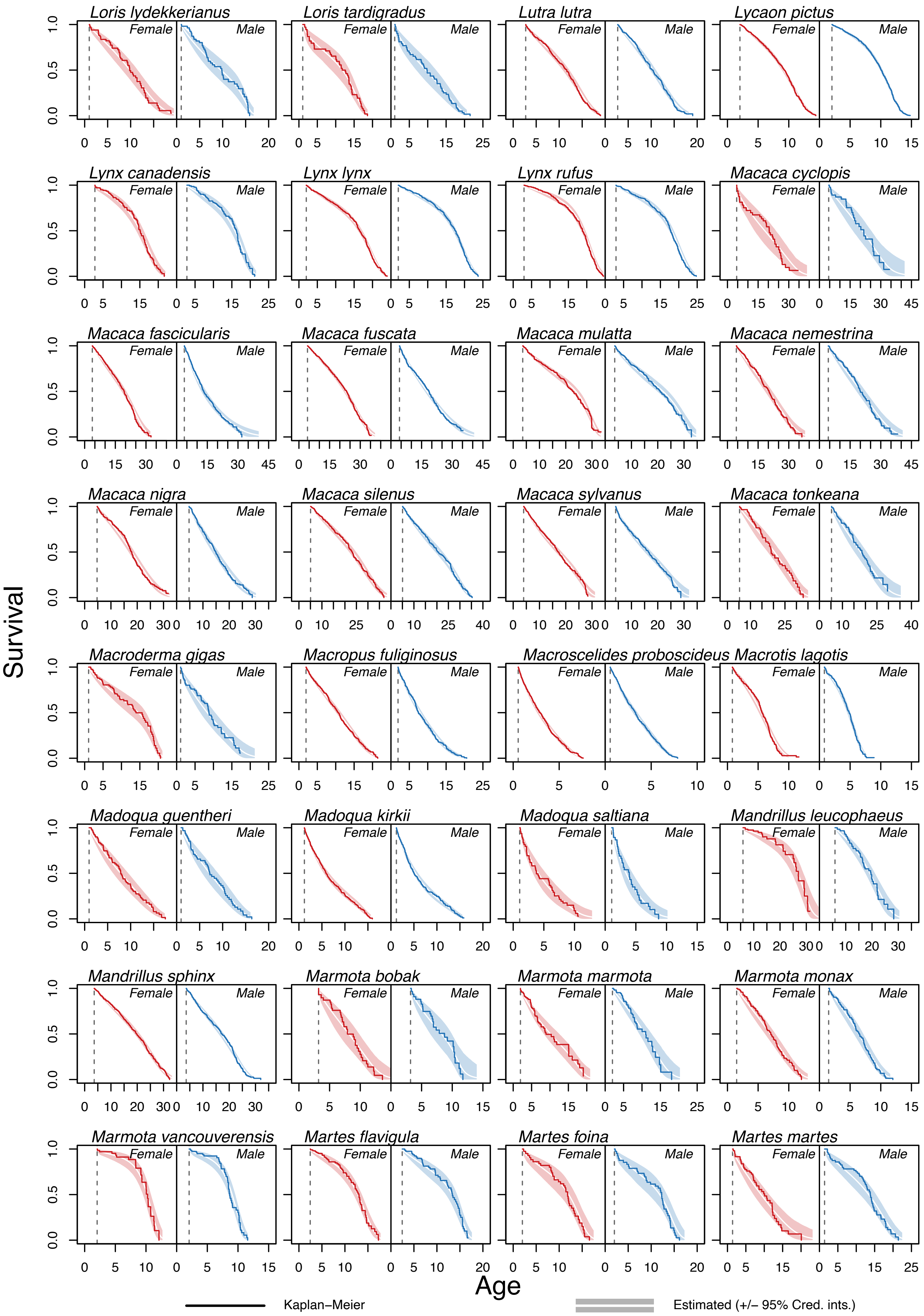

Fig. S7I. Goodness of fit plots for females and males from the Bayesian survival trajectory analysis (BaSTA) for mammals.

The red and blue polygons show the estimated survival from BaSTA with the 95% credible intervals and the dark lines are the Kaplan–Meier survival curves from the data.

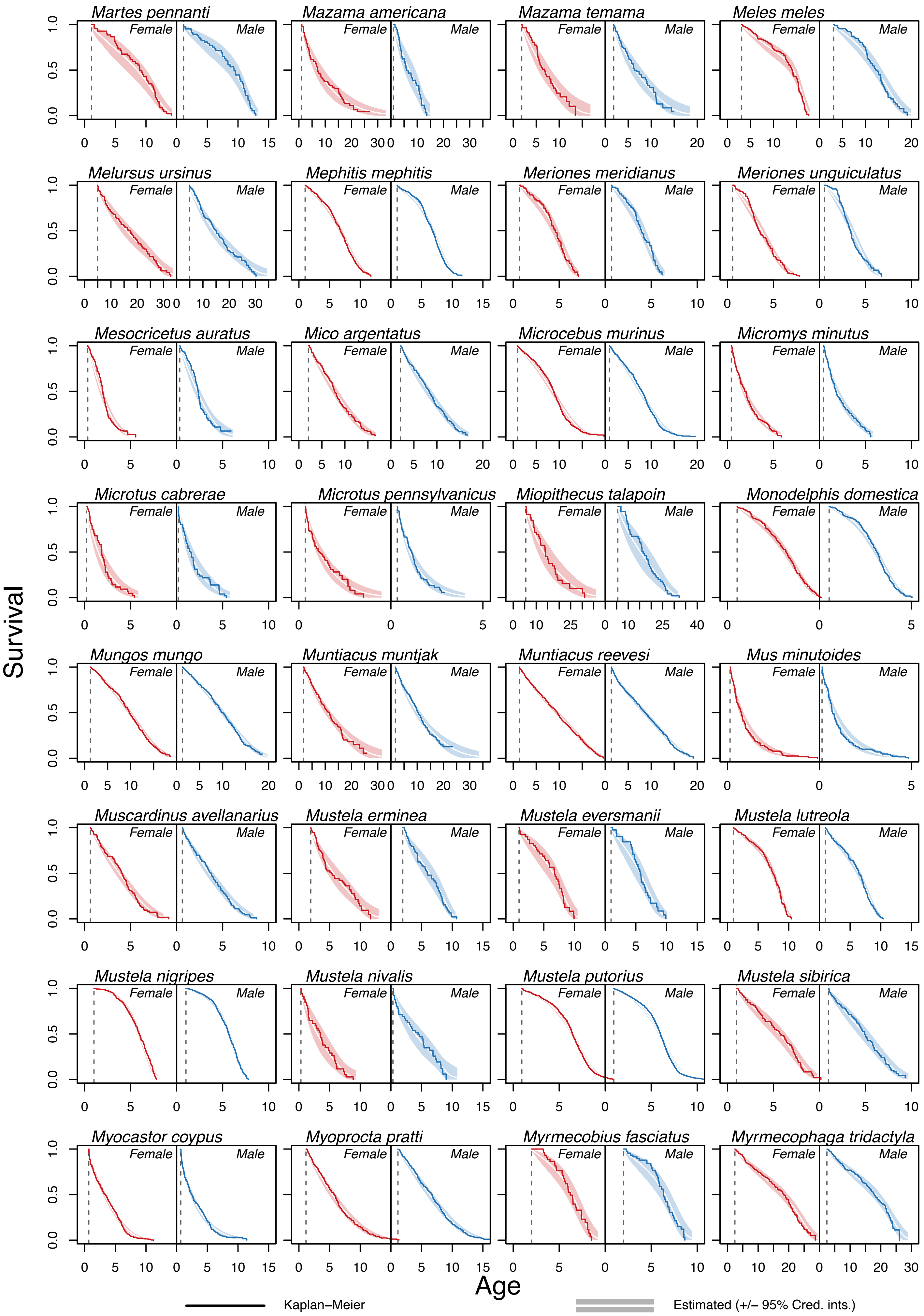

Fig. S7J. Goodness of fit plots for females and males from the Bayesian survival trajectory analysis (BaSTA) for mammals.

The red and blue polygons show the estimated survival from BaSTA with the 95% credible intervals and the dark lines are the Kaplan–Meier survival curves from the data.

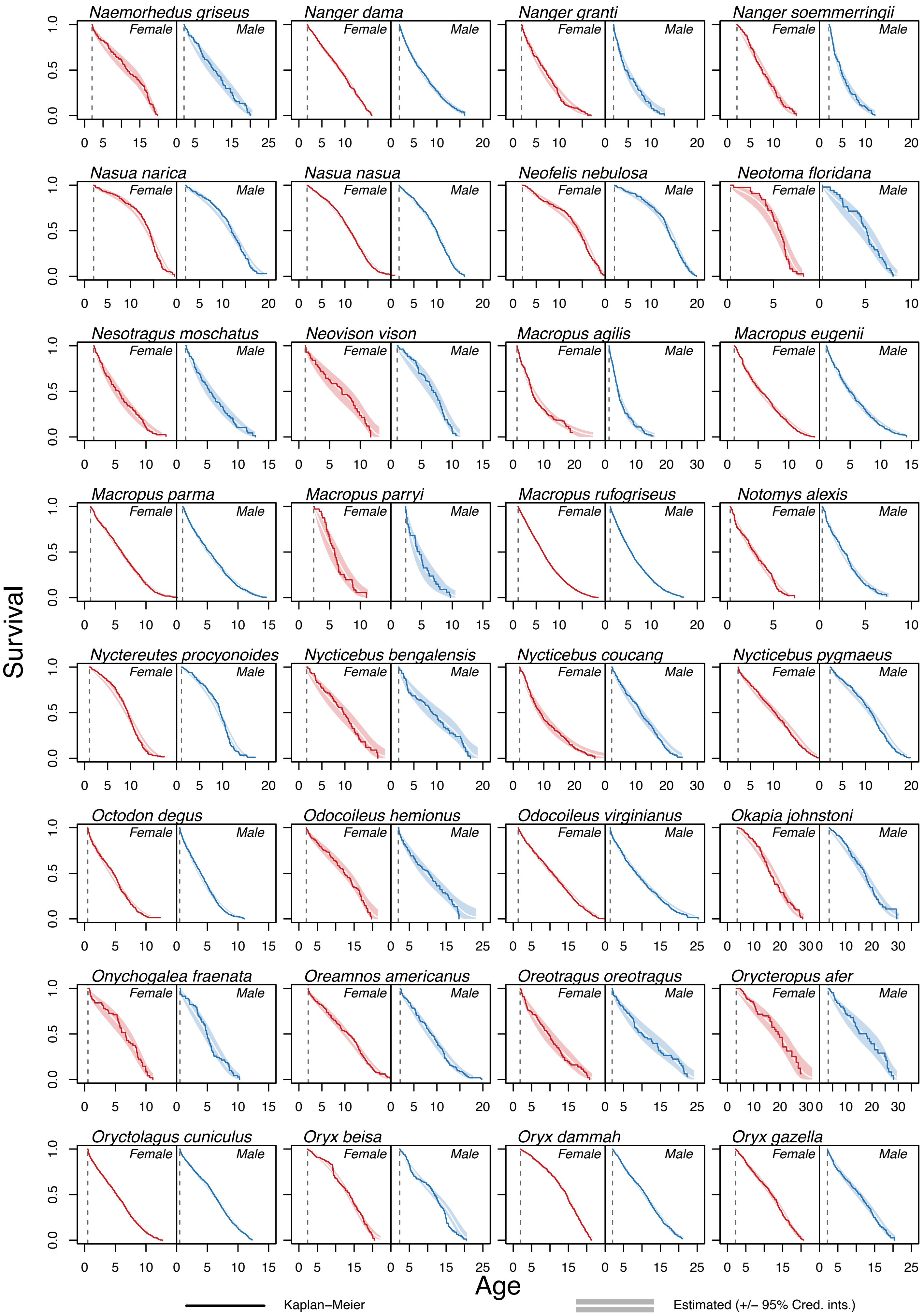

Fig. S7K. Goodness of fit plots for females and males from the Bayesian survival trajectory analysis (BaSTA) for mammals.

The red and blue polygons show the estimated survival from BaSTA with the 95% credible intervals and the dark lines are the Kaplan–Meier survival curves from the data.

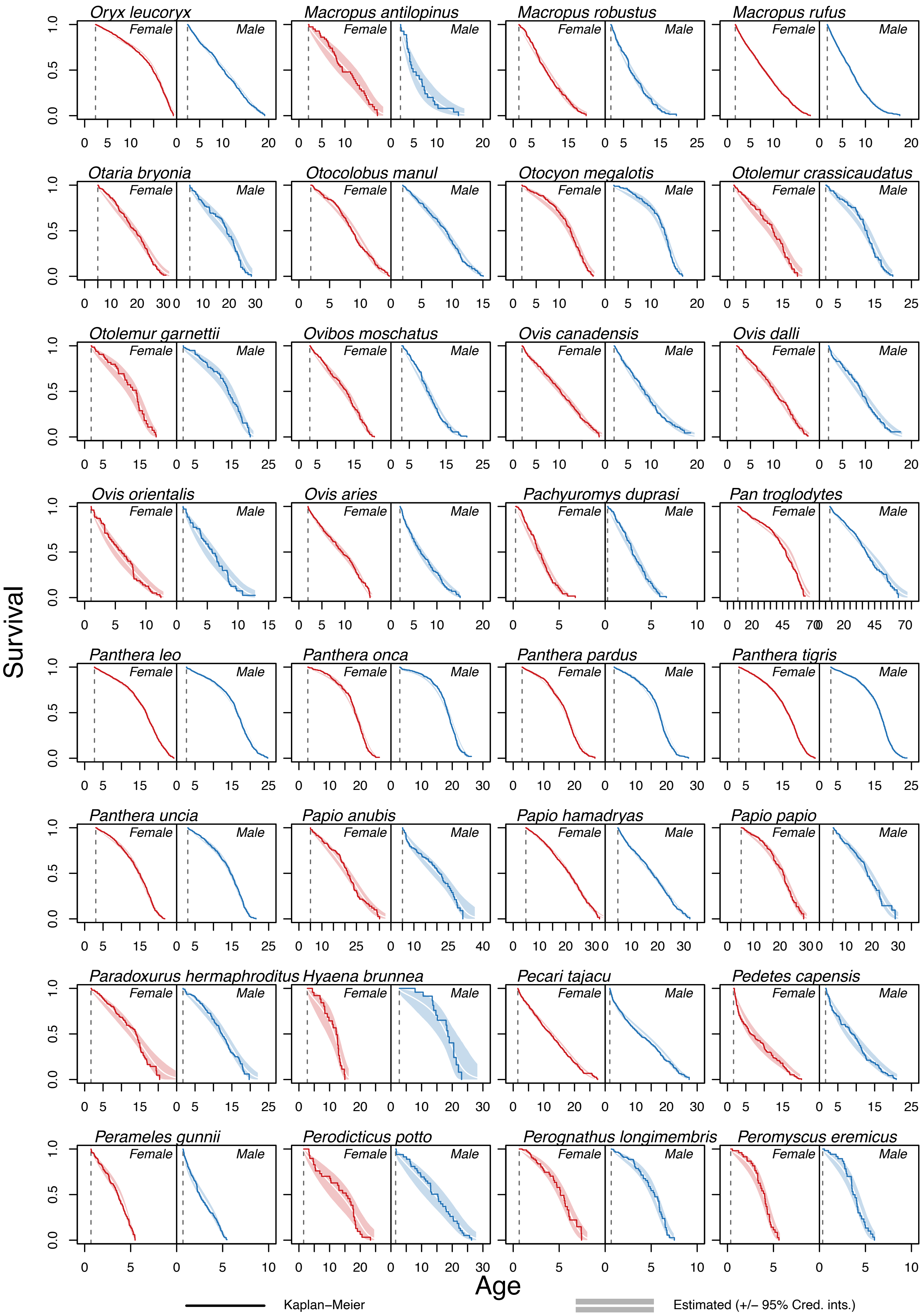

Fig. S7L. Goodness of fit plots for females and males from the Bayesian survival trajectory analysis (BaSTA) for mammals.

The red and blue polygons show the estimated survival from BaSTA with the 95% credible intervals and the dark lines are the Kaplan–Meier survival curves from the data.

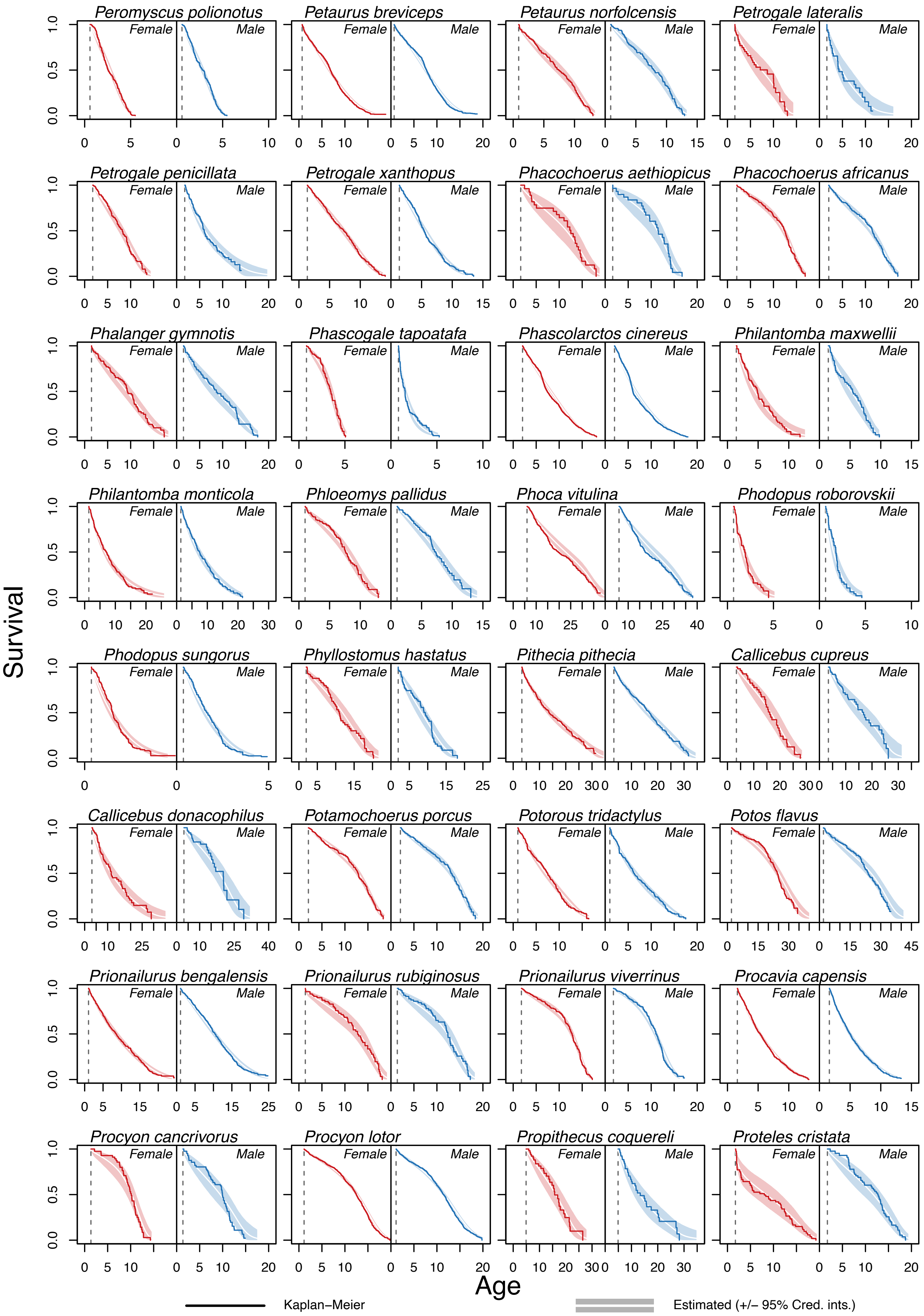

Fig. S7M. Goodness of fit plots for females and males from the Bayesian survival trajectory analysis (BaSTA) for mammals.

The red and blue polygons show the estimated survival from BaSTA with the 95% credible intervals and the dark lines are the Kaplan−Meier survival curves from the data.

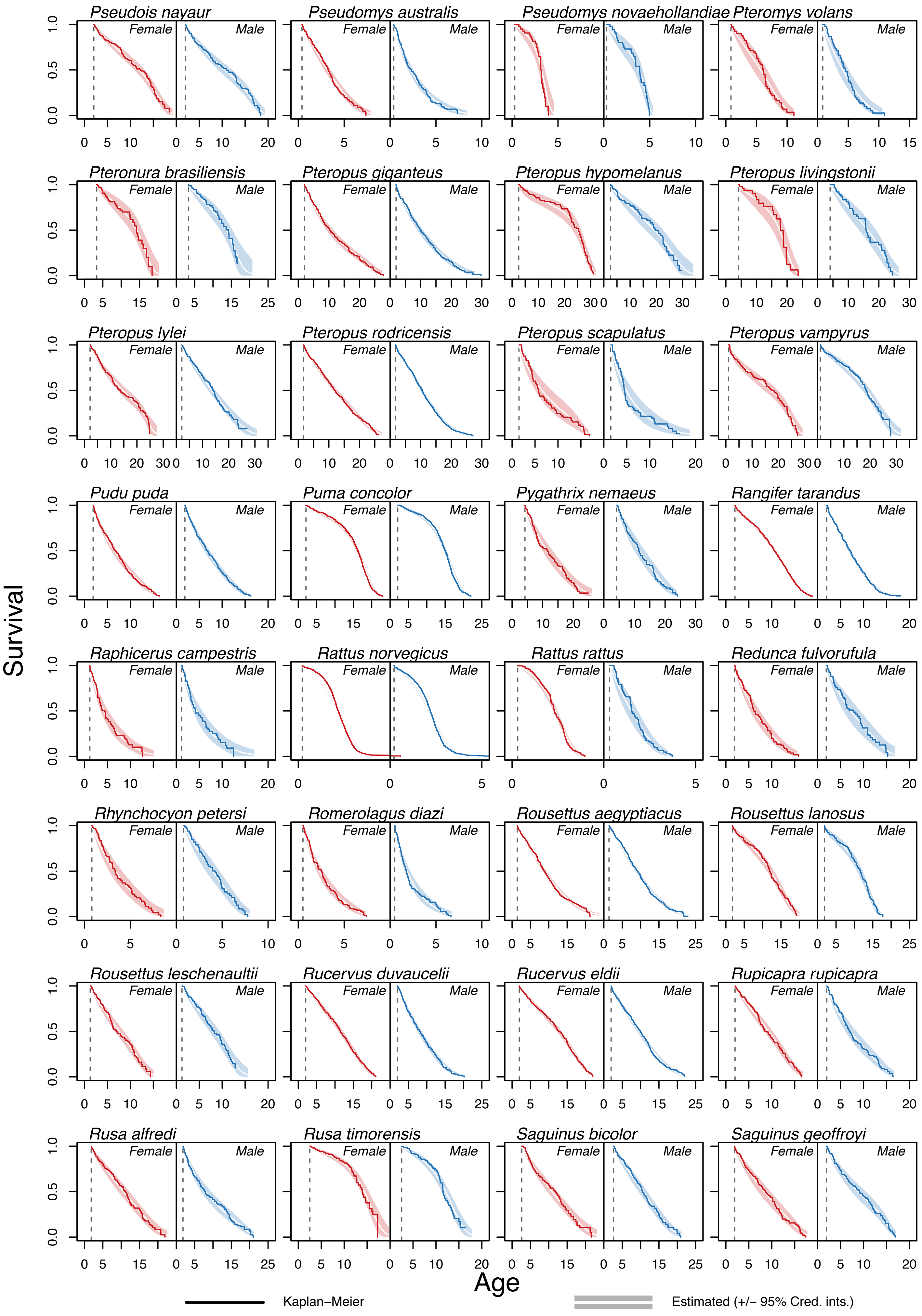

Fig. S7N. Goodness of fit plots for females and males from the Bayesian survival trajectory analysis (BaSTA) for mammals.

The red and blue polygons show the estimated survival from BaSTA with the 95% credible intervals and the dark lines are the Kaplan–Meier survival curves from the data.

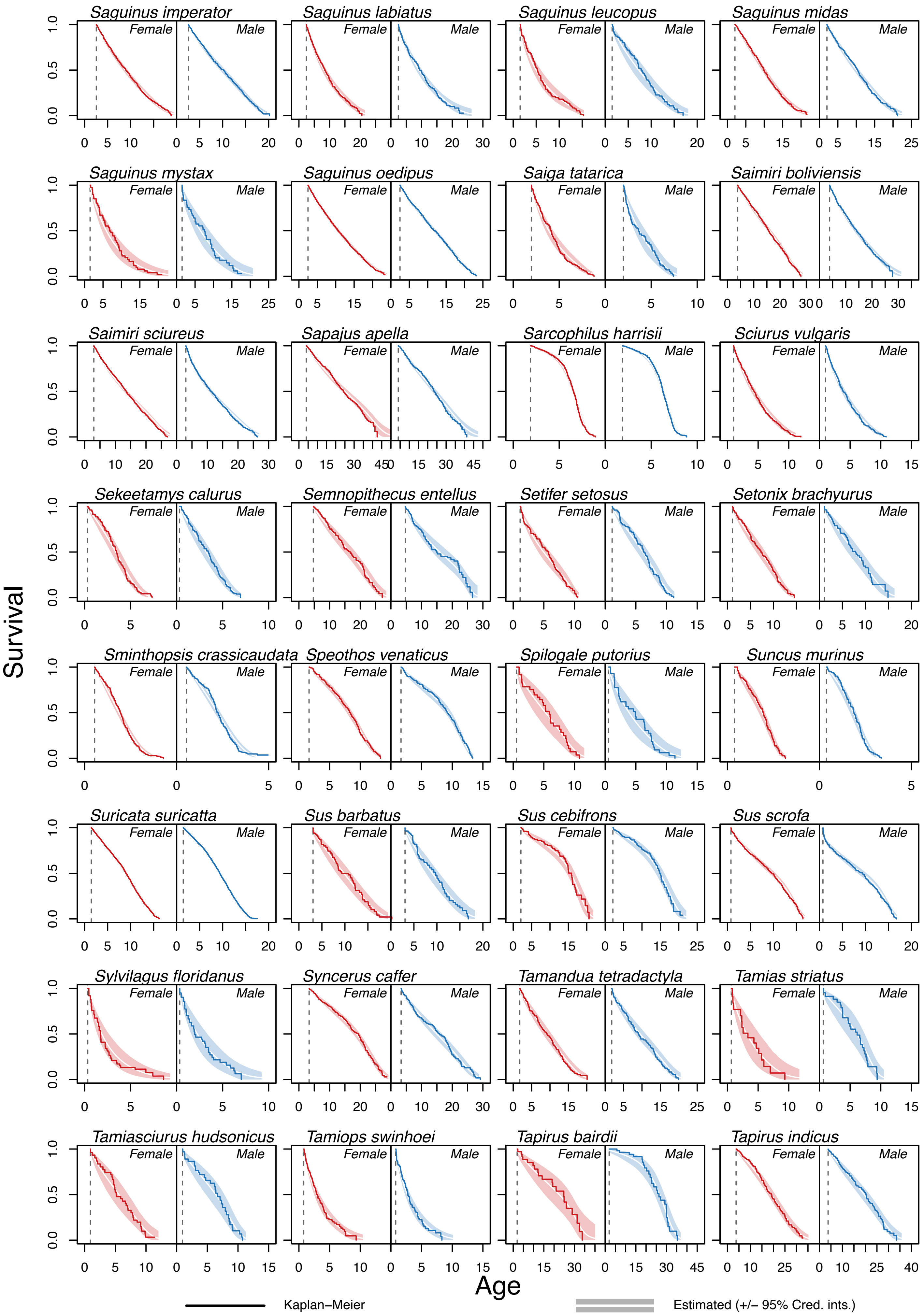

Fig. S7O. Goodness of fit plots for females and males from the Bayesian survival trajectory analysis (BaSTA) for mammals.

The red and blue polygons show the estimated survival from BaSTA with the 95% credible intervals and the dark lines are the Kaplan–Meier survival curves from the data.

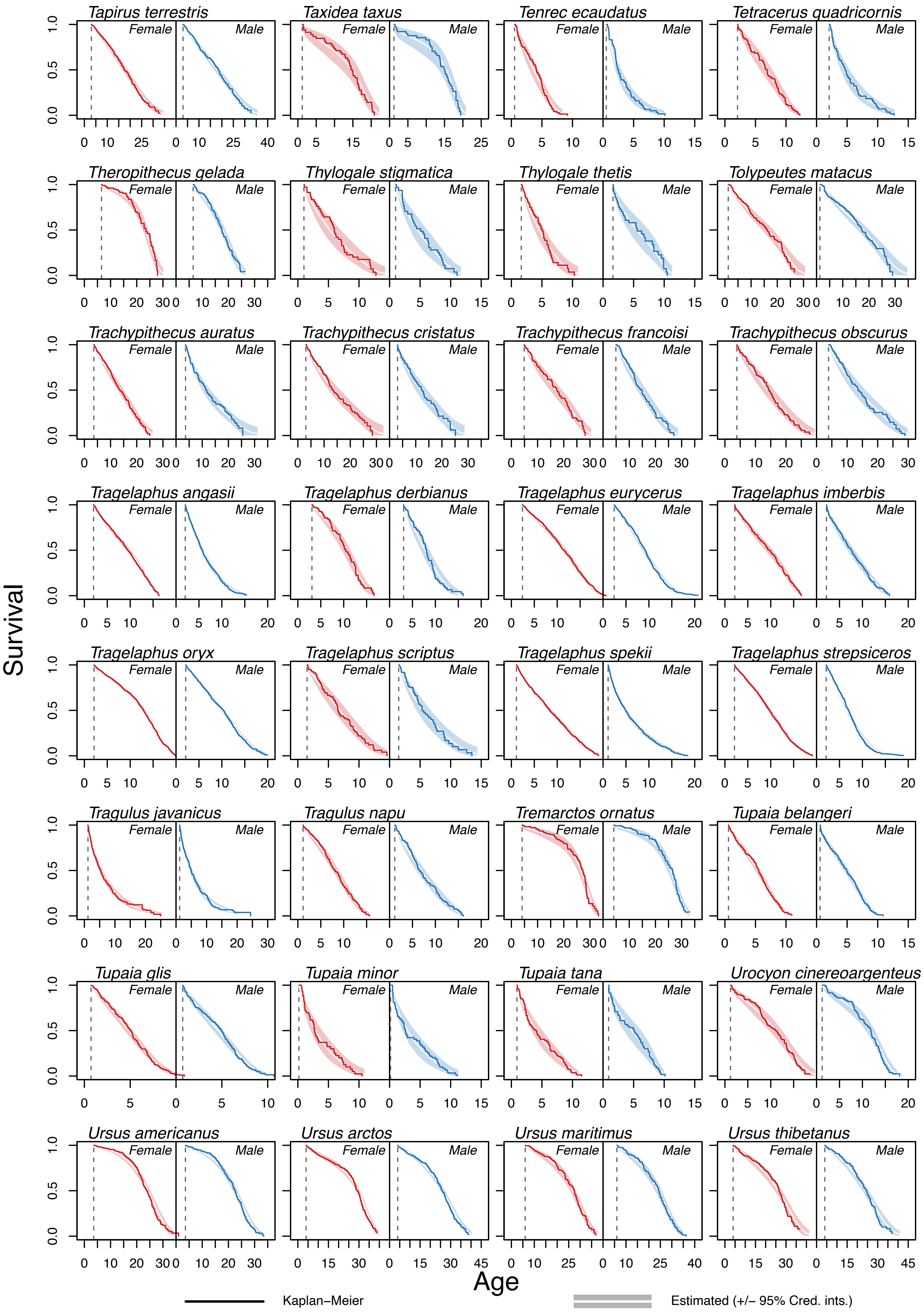

Fig. S7P. Goodness of fit plots for females and males from the Bayesian survival trajectory analysis (BaSTA) for mammals.

The red and blue polygons show the estimated survival from BaSTA with the 95% credible intervals and the dark lines are the Kaplan–Meier survival curves from the data.

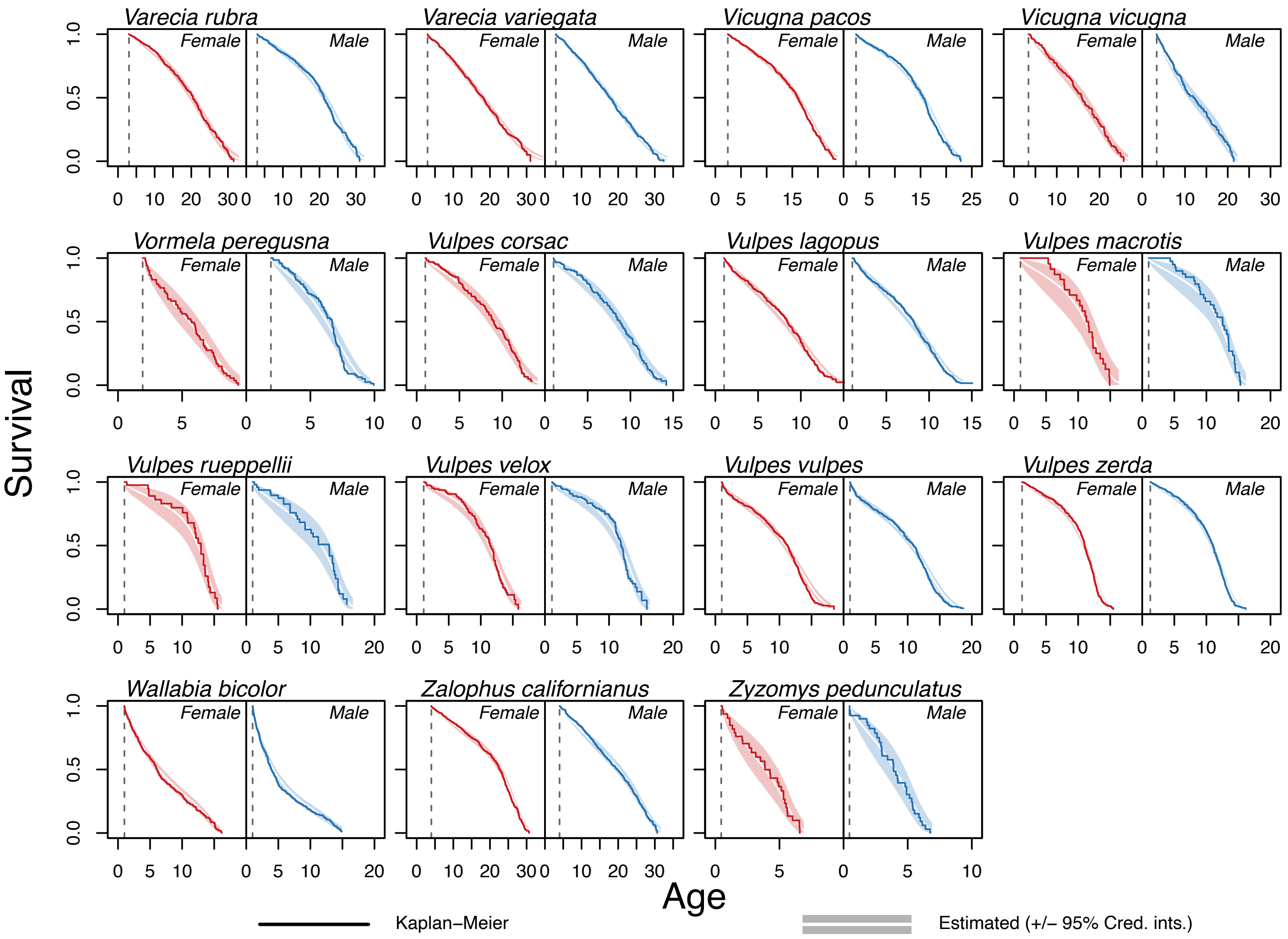

Fig. S7Q. Goodness of fit plots for females and males from the Bayesian survival trajectory analysis (BaSTA) for mammals.

The red and blue polygons show the estimated survival from BaSTA with the 95% credible intervals and the dark lines are the Kaplan–Meier survival curves from the data.

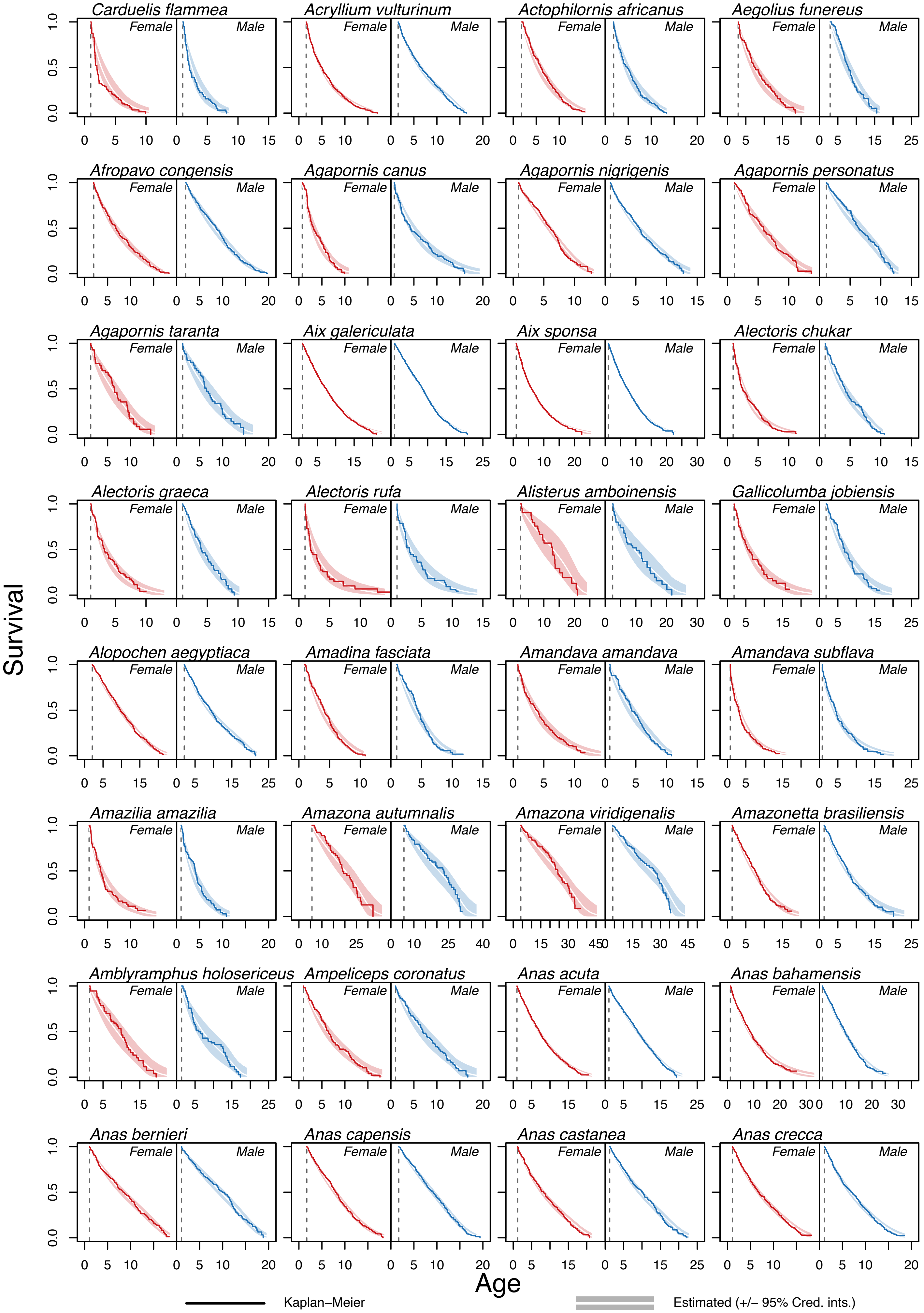

Fig. S8A. Goodness of fit plots for females and males from the Bayesian survival trajectory analysis (BaSTA) for birds.

The red and blue polygons show the estimated survival from BaSTA with the 95% credible intervals and the dark lines are the Kaplan−Meier survival curves from the data.

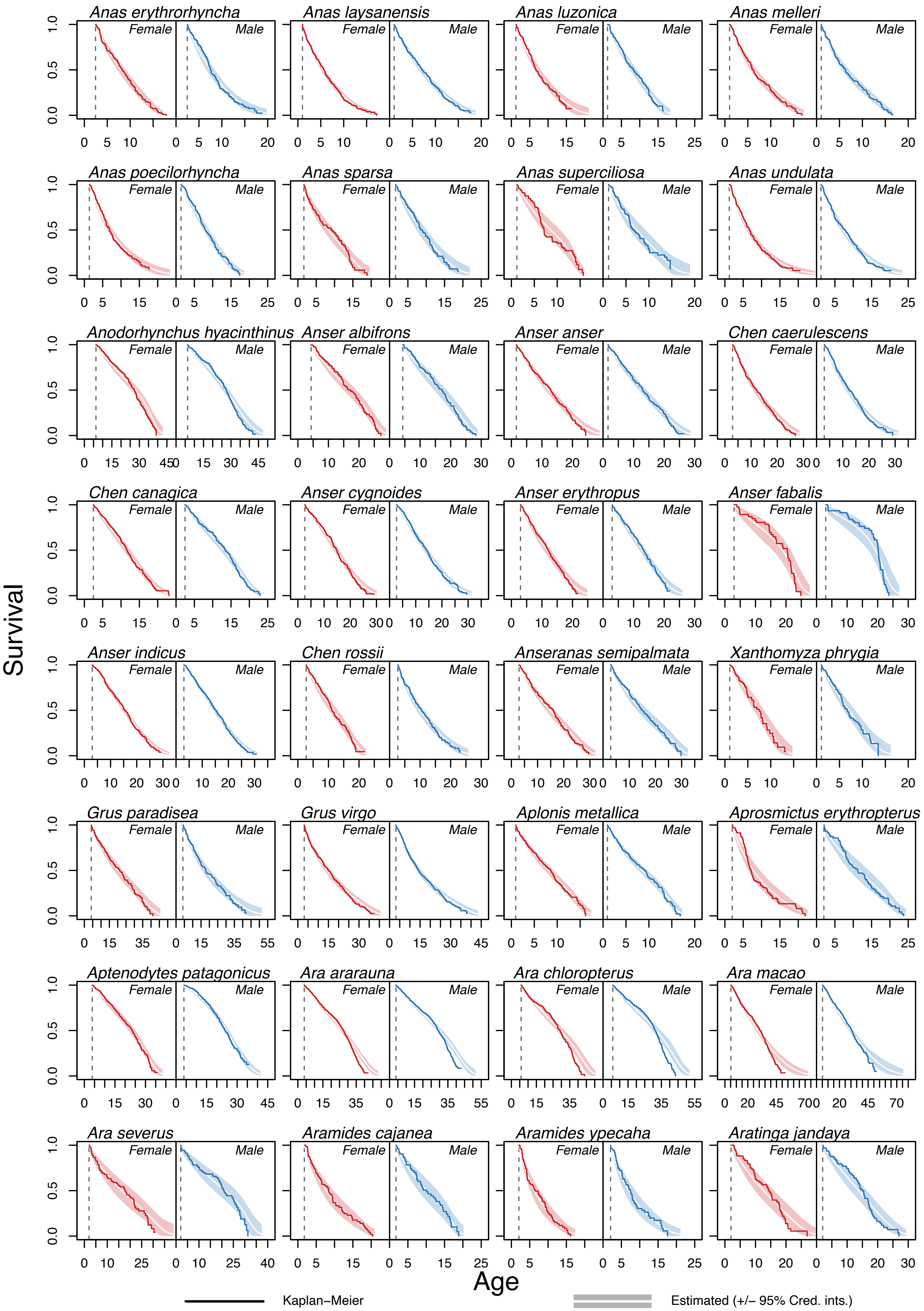

Fig. S8B. Goodness of fit plots for females and males from the Bayesian survival trajectory analysis (BaSTA) for birds.

The red and blue polygons show the estimated survival from BaSTA with the 95% credible intervals and the dark lines are the Kaplan–Meier survival curves from the data.

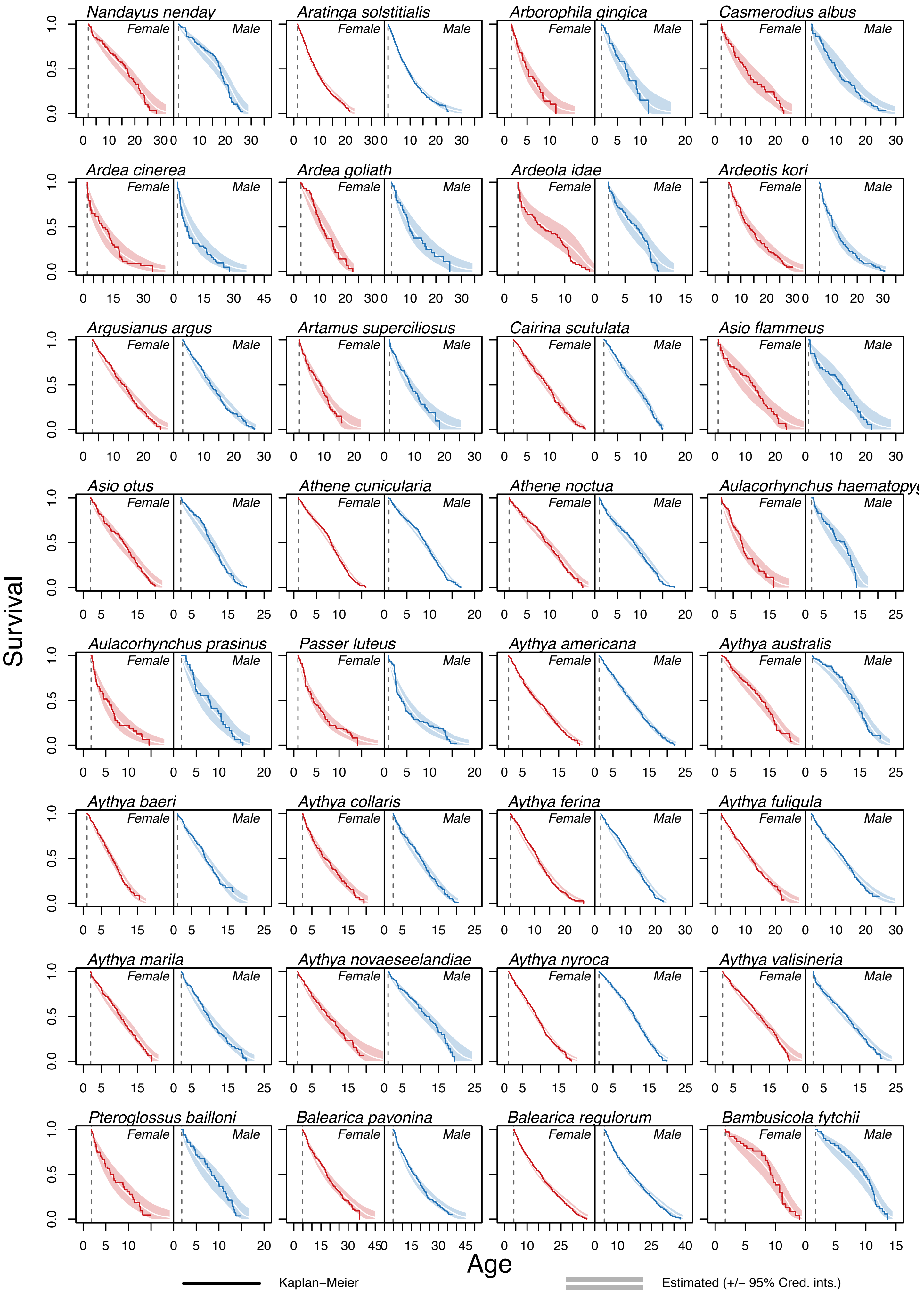

Fig. S8C. Goodness of fit plots for females and males from the Bayesian survival trajectory analysis (BaSTA) for birds.

The red and blue polygons show the estimated survival from BaSTA with the 95% credible intervals and the dark lines are the Kaplan–Meier survival curves from the data.

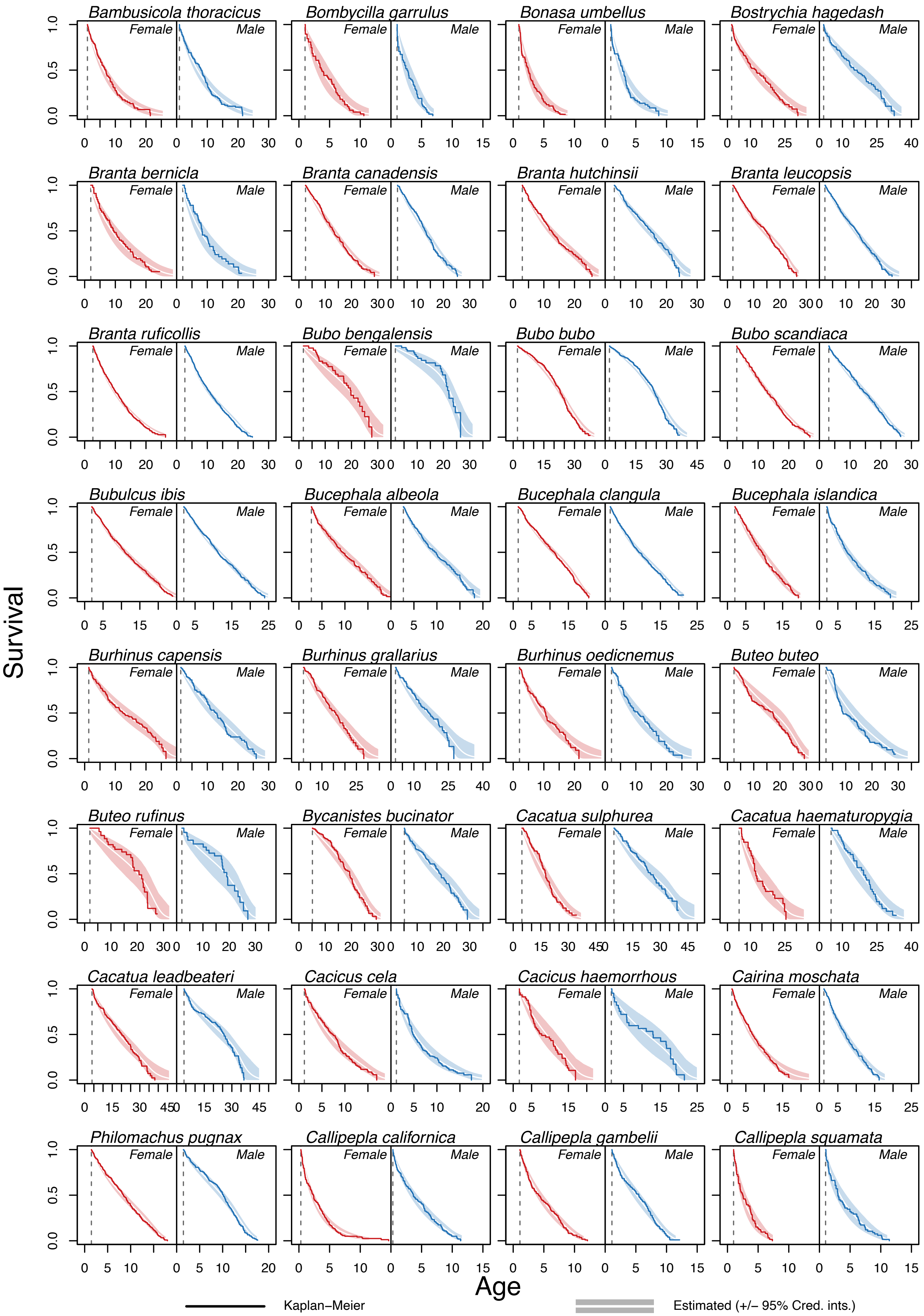

Fig. S8D. Goodness of fit plots for females and males from the Bayesian survival trajectory analysis (BaSTA) for birds.

The red and blue polygons show the estimated survival from BaSTA with the 95% credible intervals and the dark lines are the Kaplan–Meier survival curves from the data.

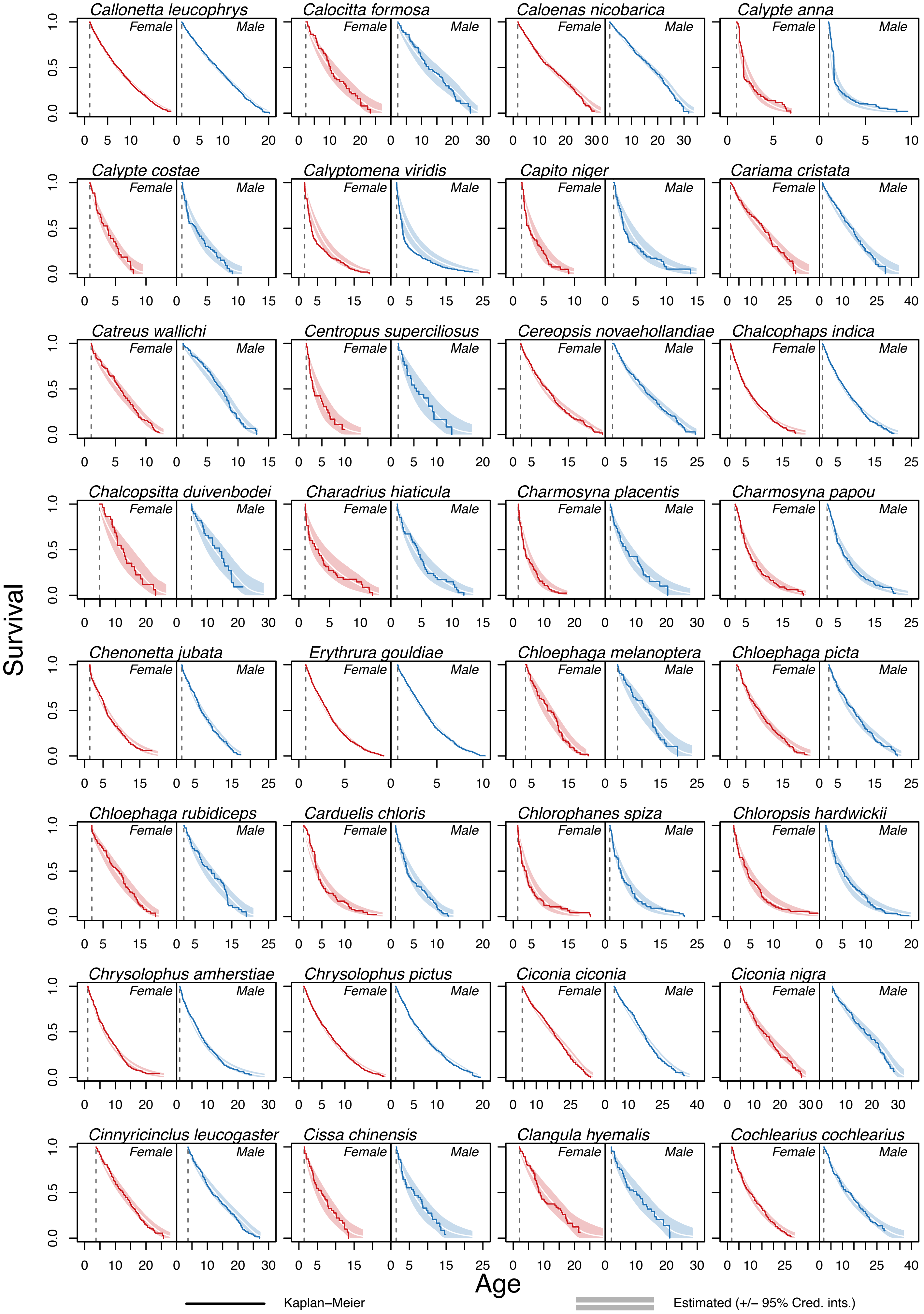

Fig. S8E. Goodness of fit plots for females and males from the Bayesian survival trajectory analysis (BaSTA) for birds.

The red and blue polygons show the estimated survival from BaSTA with the 95% credible intervals and the dark lines are the Kaplan–Meier survival curves from the data.

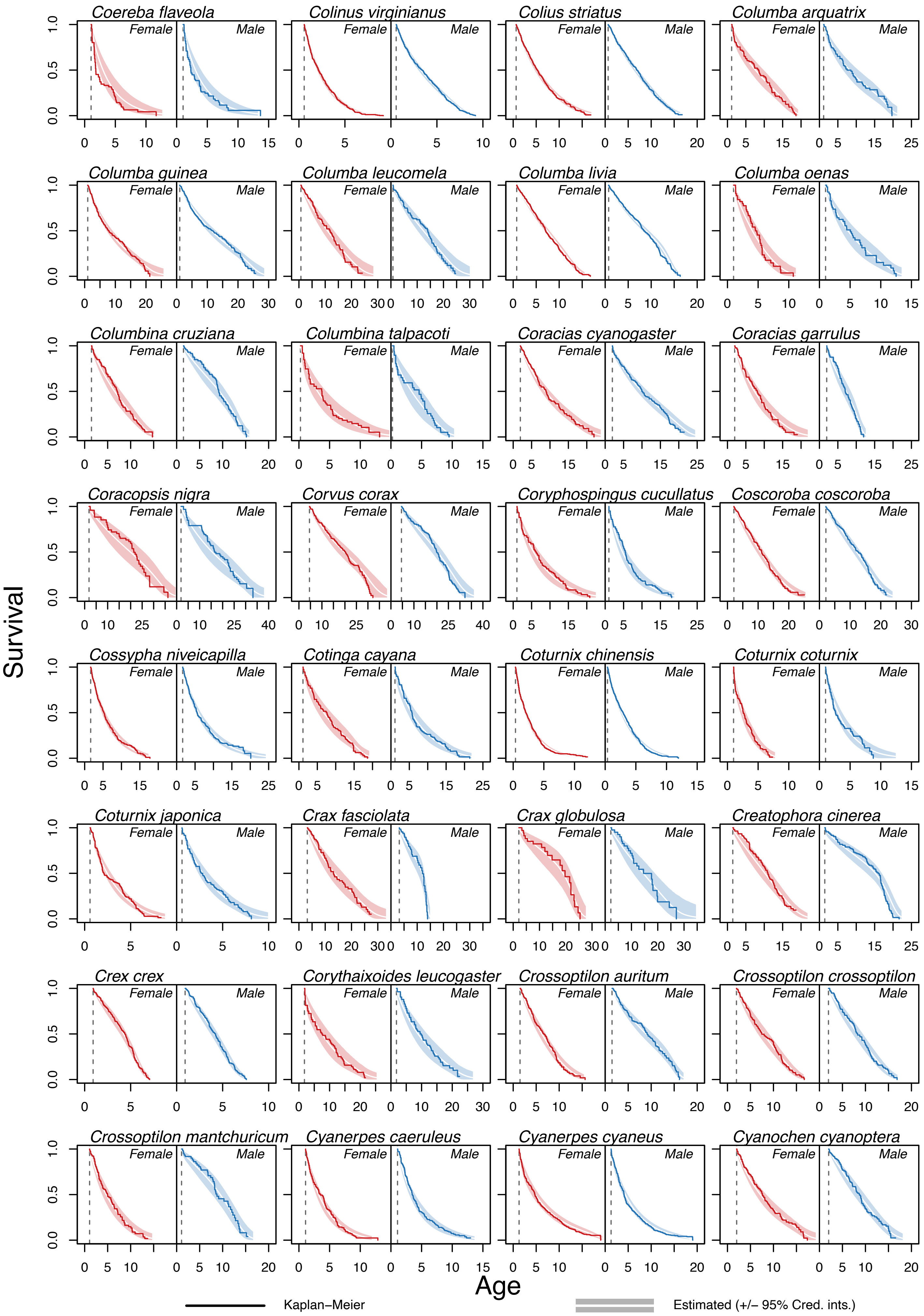

Fig. S8F. Goodness of fit plots for females and males from the Bayesian survival trajectory analysis (BaSTA) for birds.

The red and blue polygons show the estimated survival from BaSTA with the 95% credible intervals and the dark lines are the Kaplan−Meier survival curves from the data.

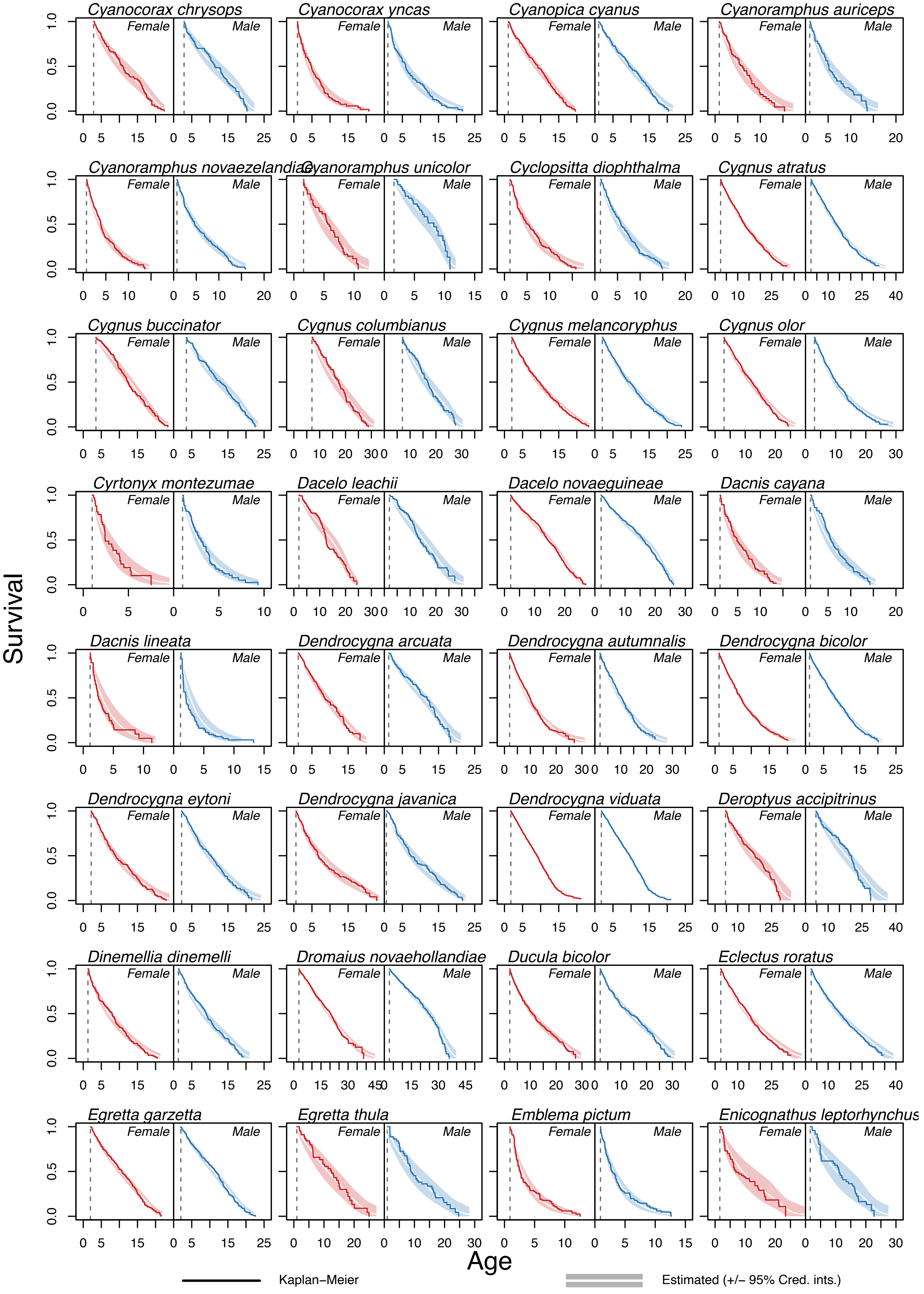

Fig. S8G. Goodness of fit plots for females and males from the Bayesian survival trajectory analysis (BaSTA) for birds.

The red and blue polygons show the estimated survival from BaSTA with the 95% credible intervals and the dark lines are the Kaplan–Meier survival curves from the data.

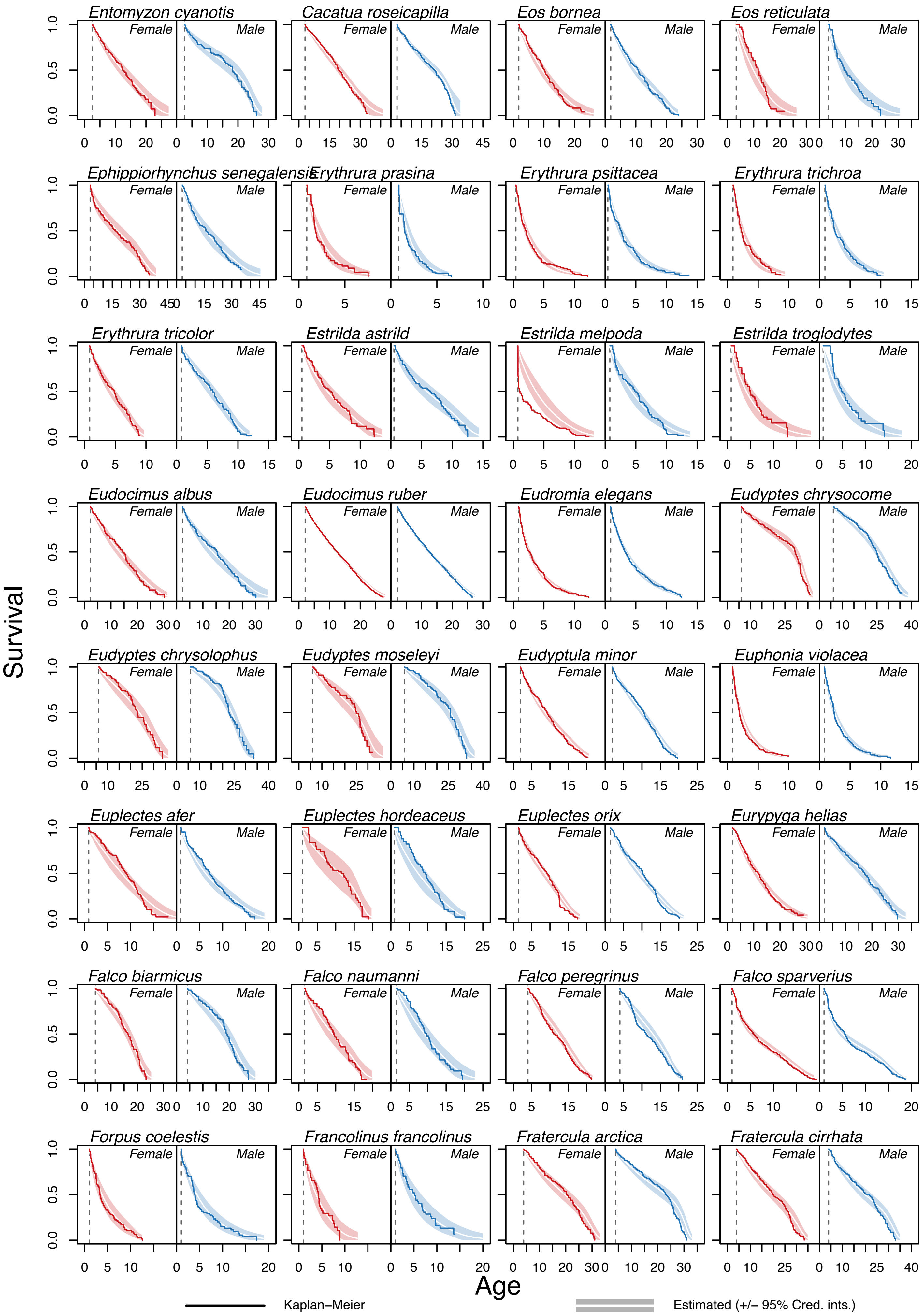

Fig. S8H. Goodness of fit plots for females and males from the Bayesian survival trajectory analysis (BaSTA) for birds.

The red and blue polygons show the estimated survival from BaSTA with the 95% credible intervals and the dark lines are the Kaplan–Meier survival curves from the data.

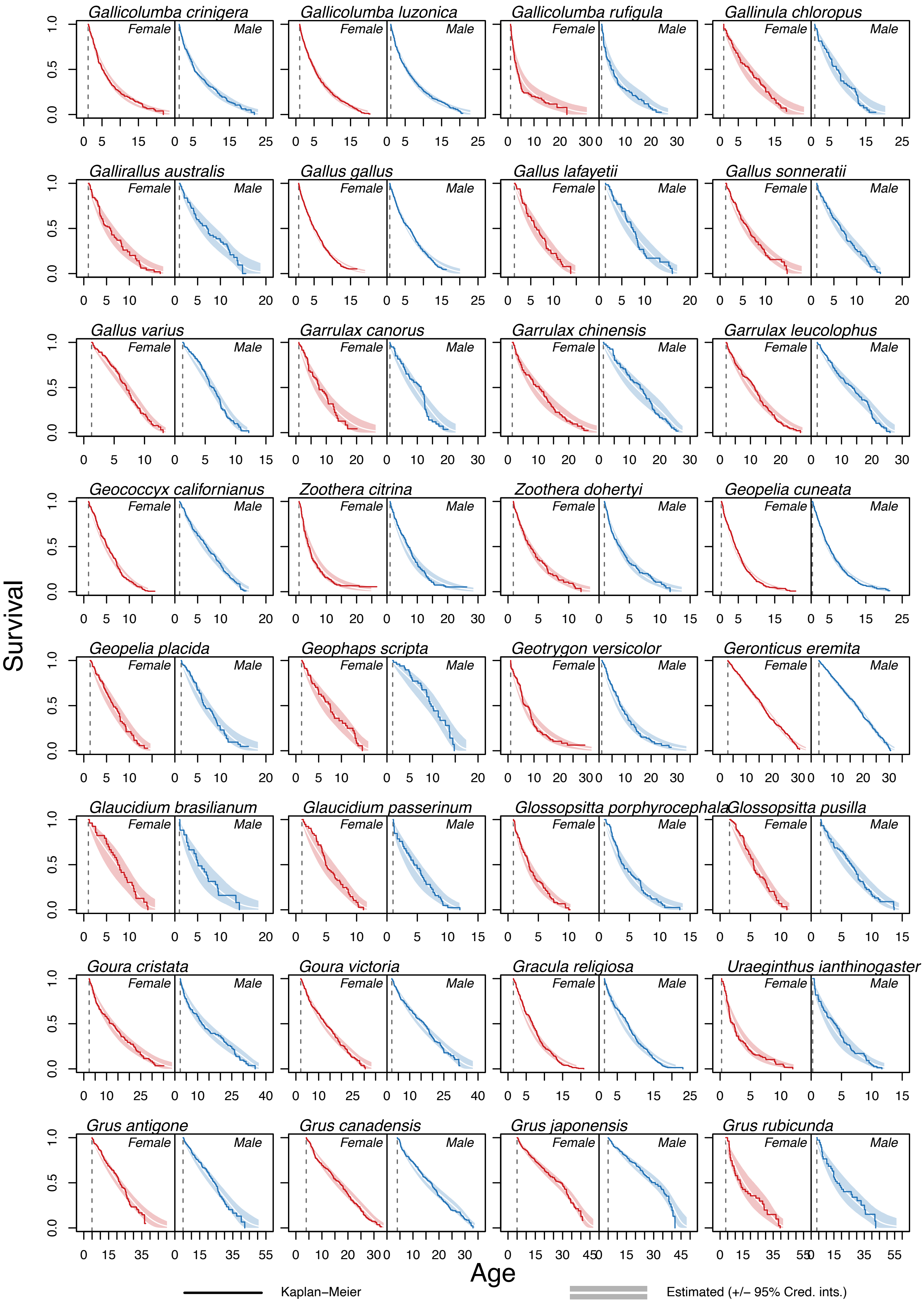

Fig. S8l. Goodness of fit plots for females and males from the Bayesian survival trajectory analysis (BaSTA) for birds.

The red and blue polygons show the estimated survival from BaSTA with the 95% credible intervals and the dark lines are the Kaplan–Meier survival curves from the data.

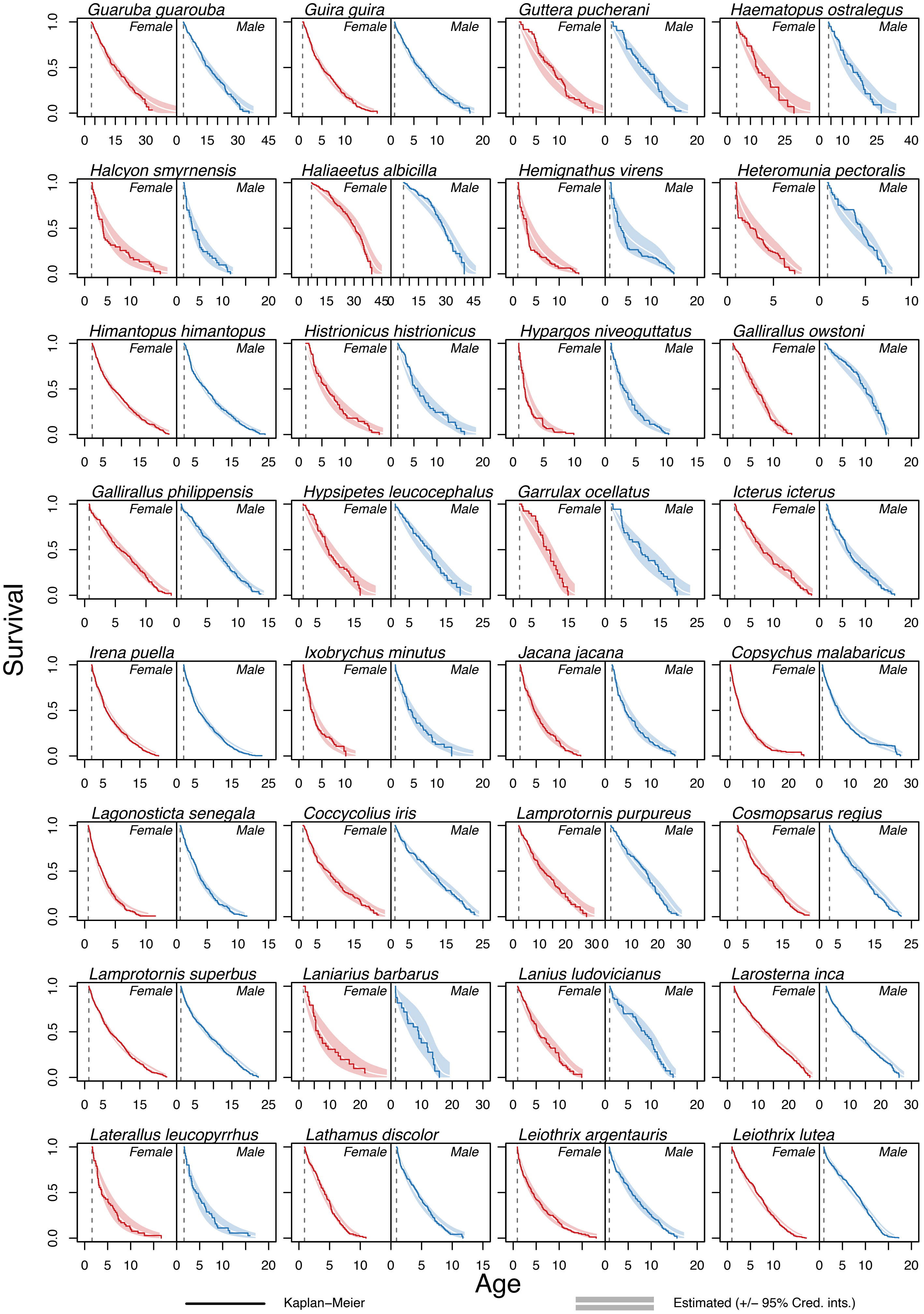

Fig. S8J. Goodness of fit plots for females and males from the Bayesian survival trajectory analysis (BaSTA) for birds.

The red and blue polygons show the estimated survival from BaSTA with the 95% credible intervals and the dark lines are the Kaplan–Meier survival curves from the data.

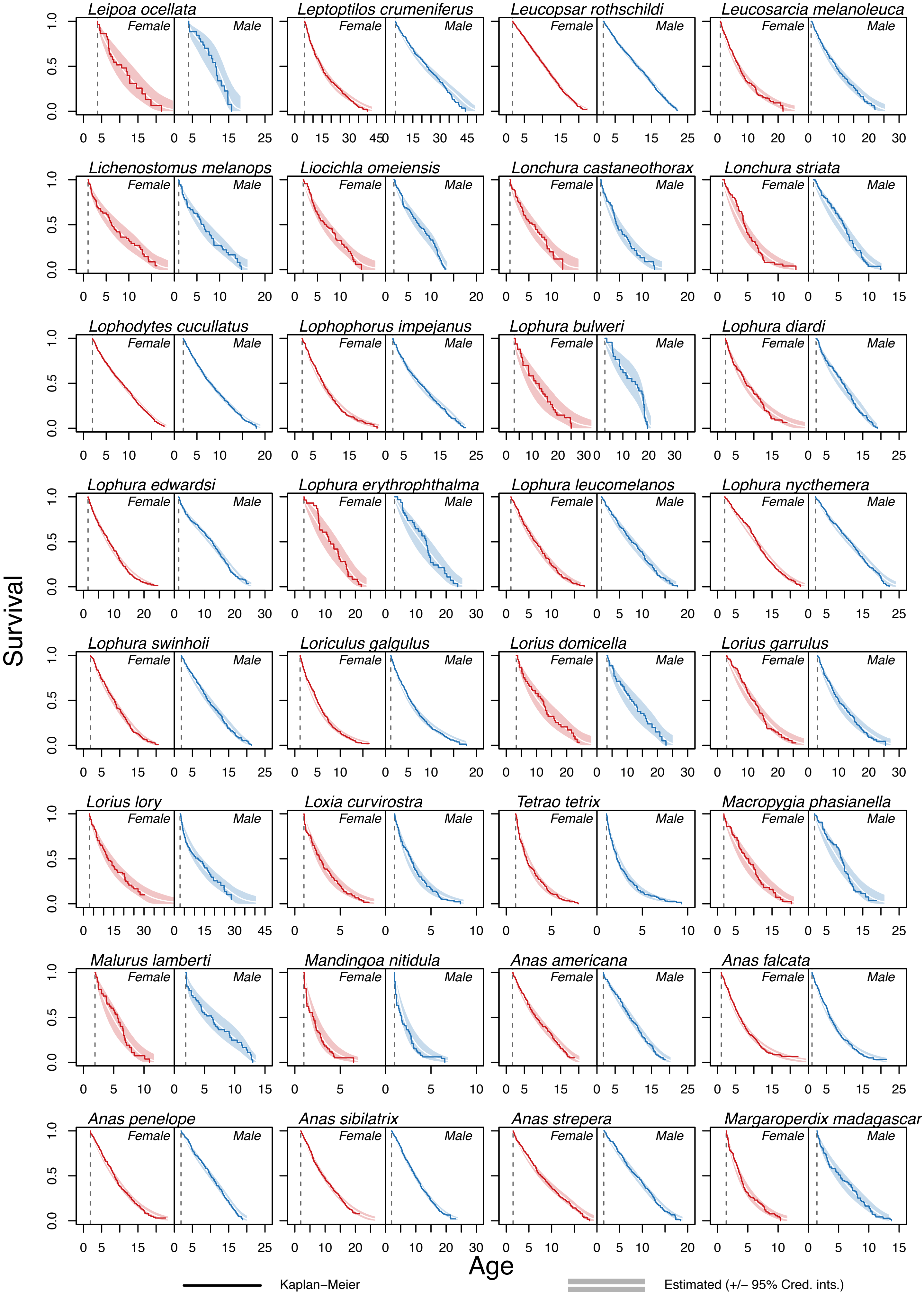

Fig. S8K. Goodness of fit plots for females and males from the Bayesian survival trajectory analysis (BaSTA) for birds.

The red and blue polygons show the estimated survival from BaSTA with the 95% credible intervals and the dark lines are the Kaplan−Meier survival curves from the data.

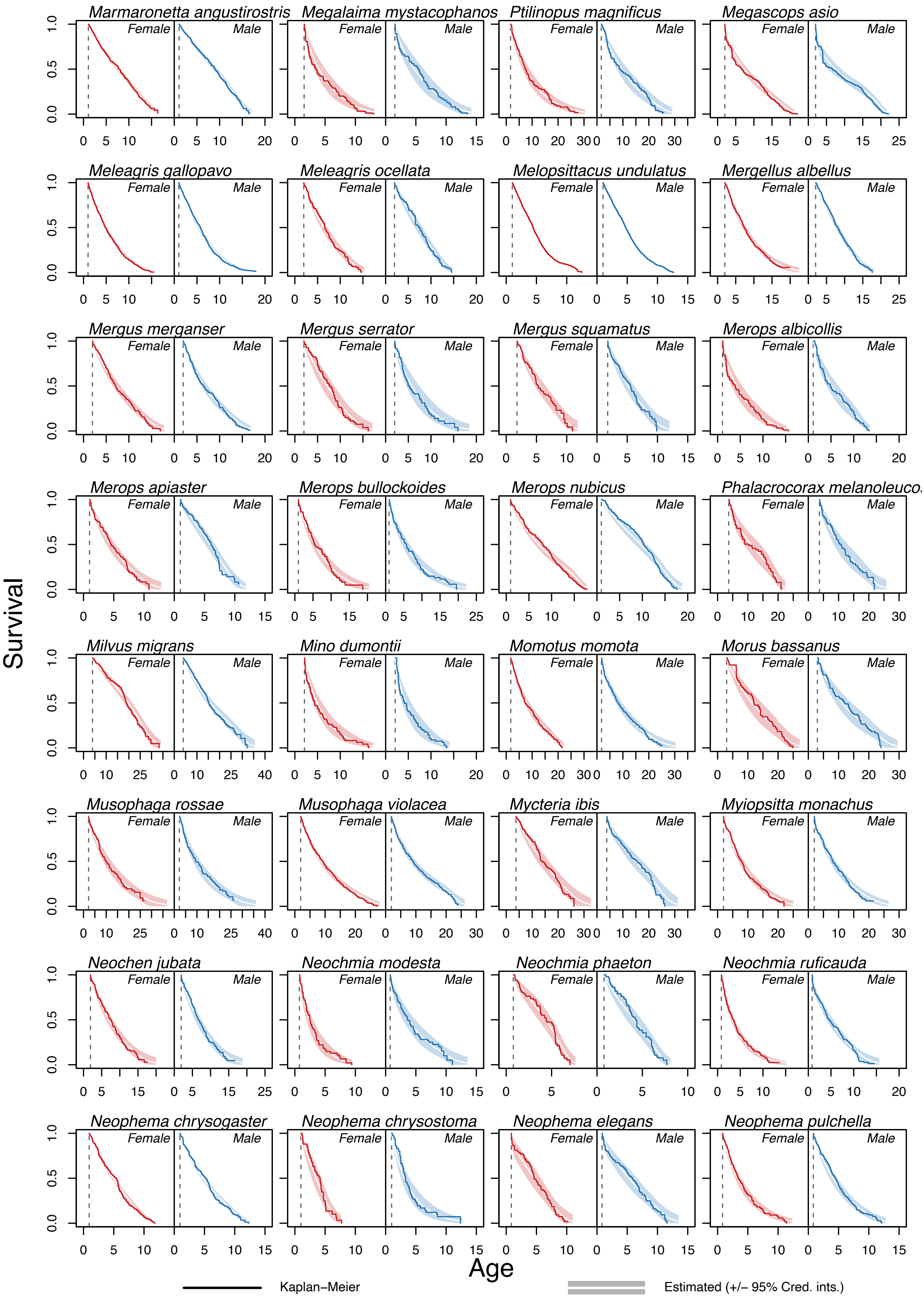

Fig. S8L. Goodness of fit plots for females and males from the Bayesian survival trajectory analysis (BaSTA) for birds.

The red and blue polygons show the estimated survival from BaSTA with the 95% credible intervals and the dark lines are the Kaplan–Meier survival curves from the data.

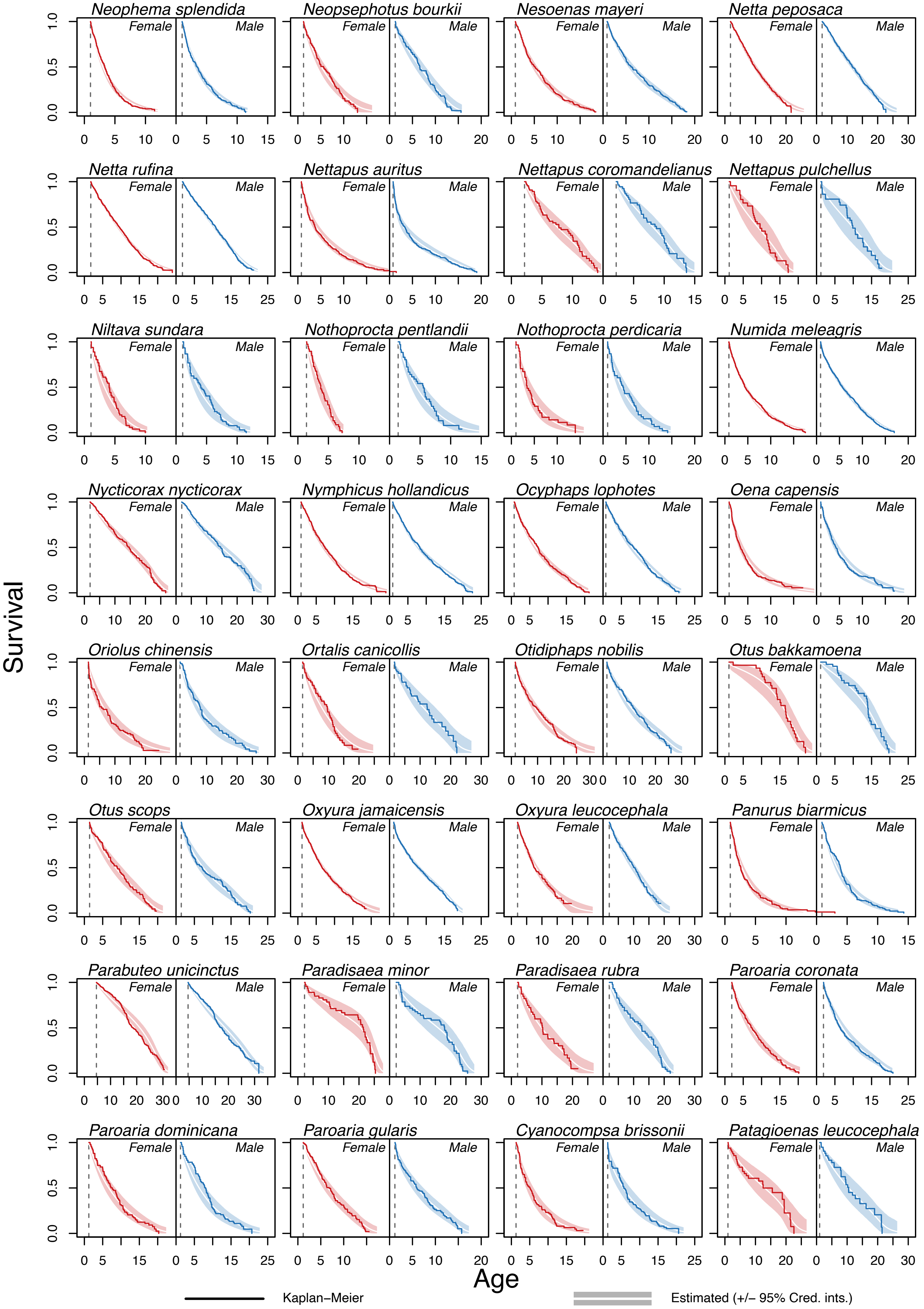

Fig. S8M. Goodness of fit plots for females and males from the Bayesian survival trajectory analysis (BaSTA) for birds.

The red and blue polygons show the estimated survival from BaSTA with the 95% credible intervals and the dark lines are the Kaplan–Meier survival curves from the data.

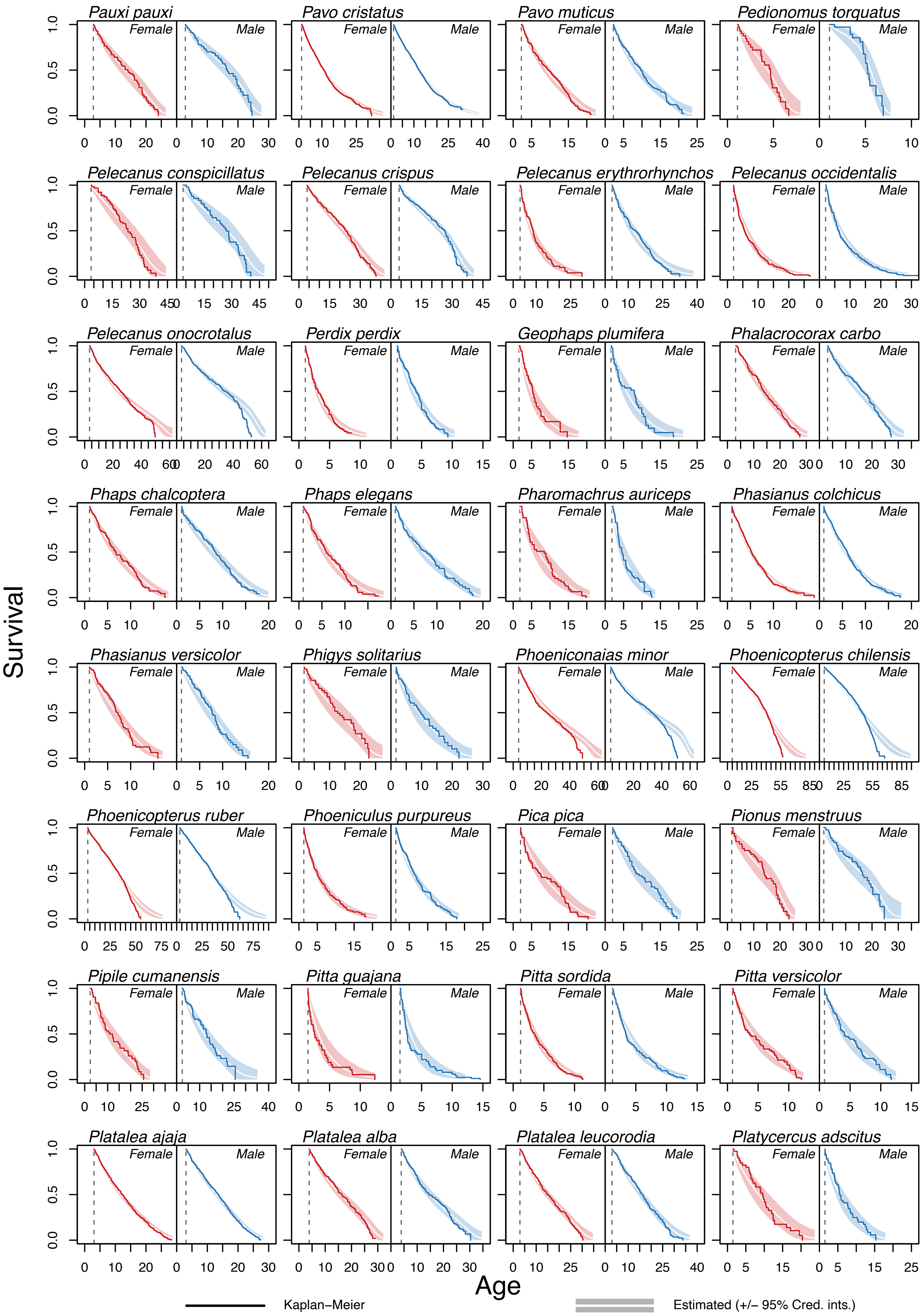

Fig. S8N. Goodness of fit plots for females and males from the Bayesian survival trajectory analysis (BaSTA) for birds.

The red and blue polygons show the estimated survival from BaSTA with the 95% credible intervals and the dark lines are the Kaplan–Meier survival curves from the data.

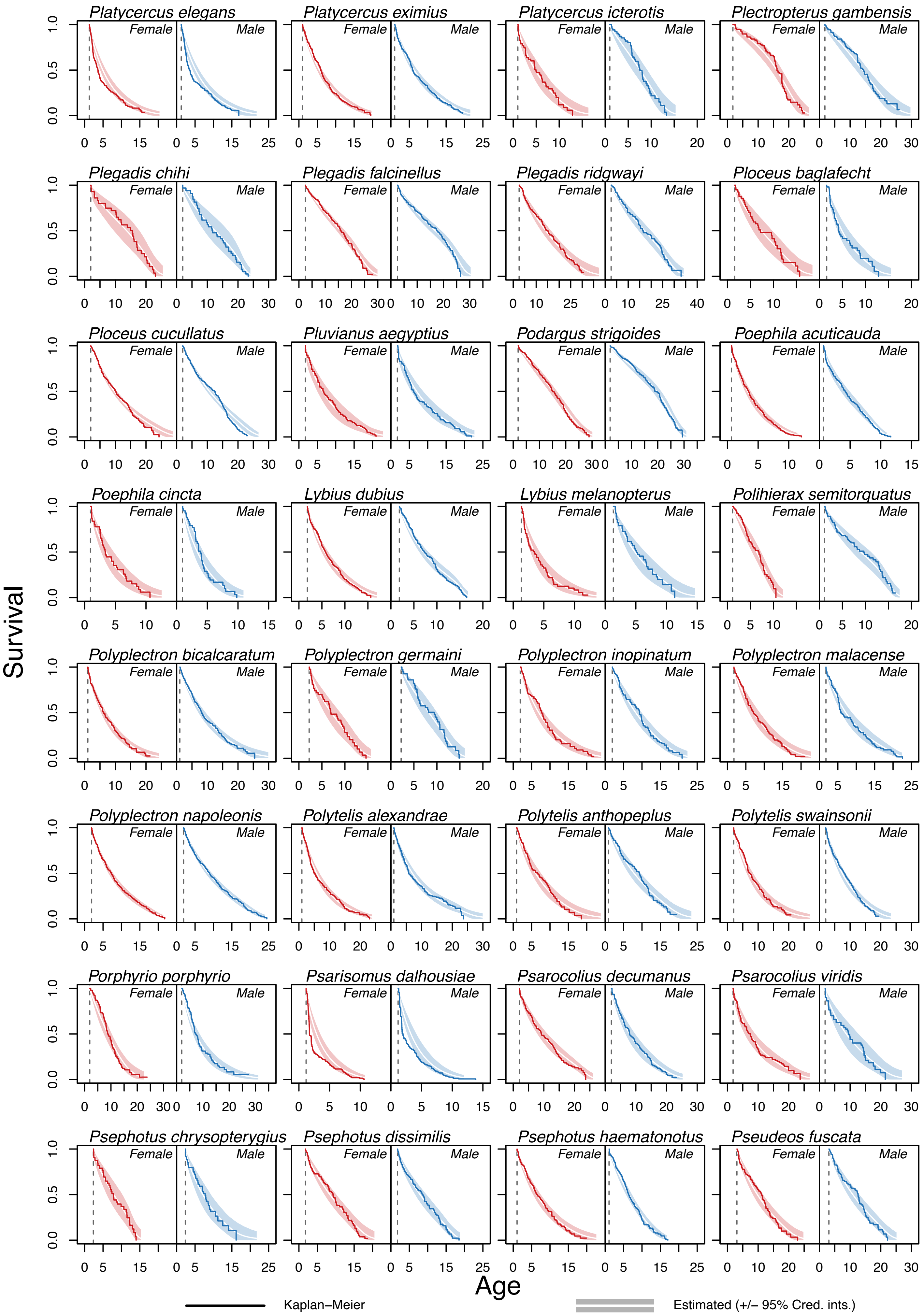

Fig. S8O. Goodness of fit plots for females and males from the Bayesian survival trajectory analysis (BaSTA) for birds.

The red and blue polygons show the estimated survival from BaSTA with the 95% credible intervals and the dark lines are the Kaplan–Meier survival curves from the data.

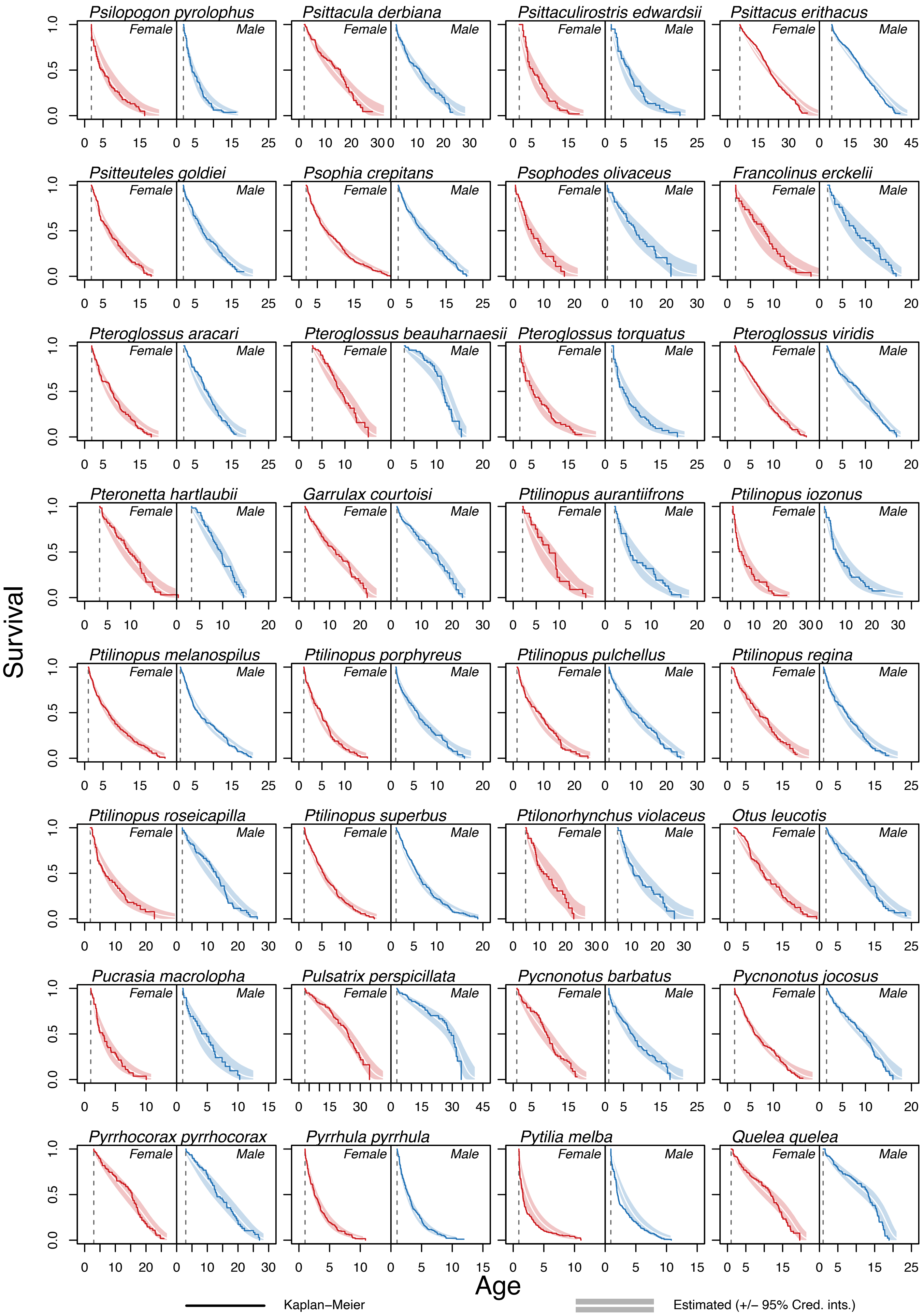

Fig. S8P. Goodness of fit plots for females and males from the Bayesian survival trajectory analysis (BaSTA) for birds.

The red and blue polygons show the estimated survival from BaSTA with the 95% credible intervals and the dark lines are the Kaplan–Meier survival curves from the data.

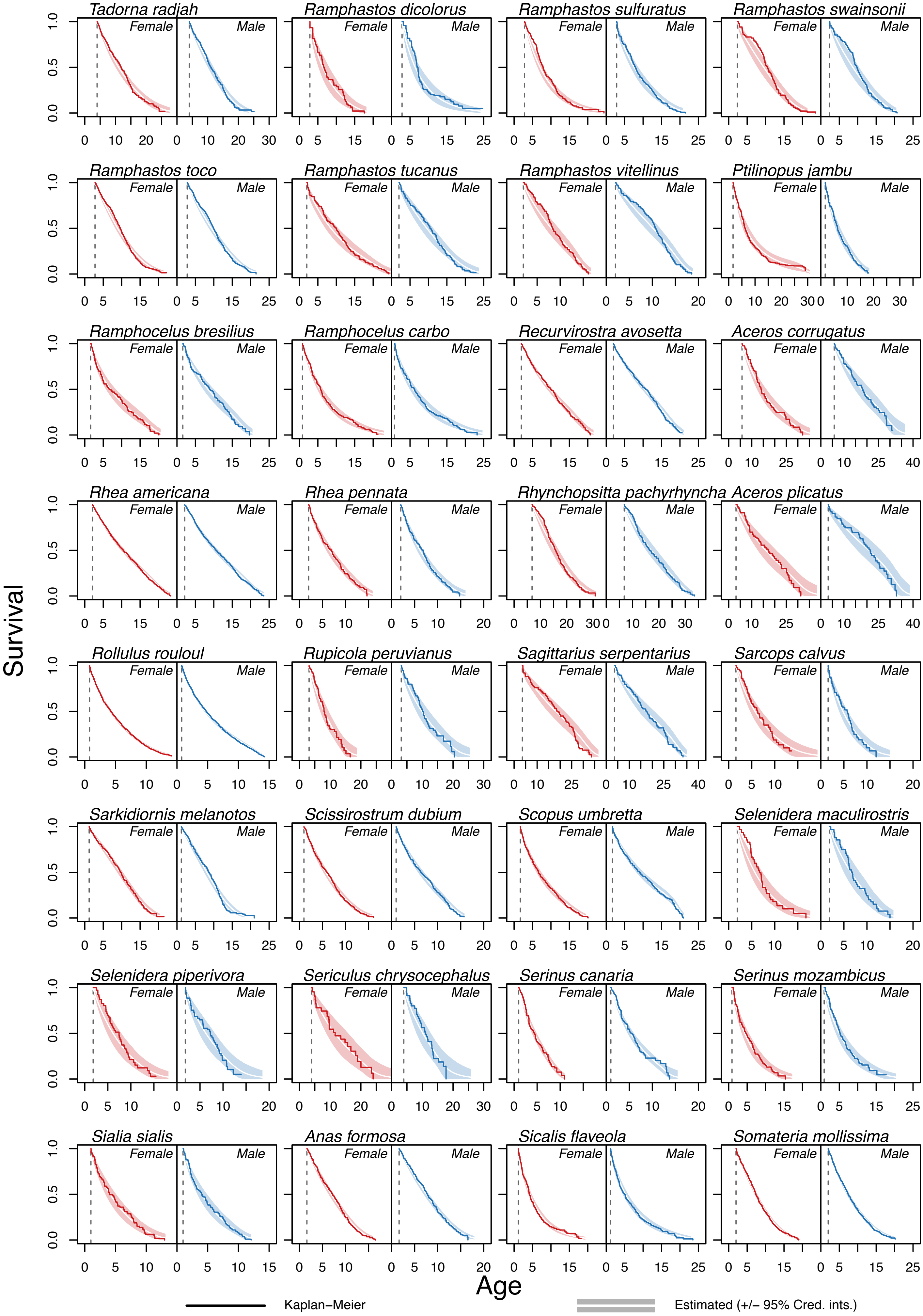

Fig. S8Q. Goodness of fit plots for females and males from the Bayesian survival trajectory analysis (BaSTA) for birds.

The red and blue polygons show the estimated survival from BaSTA with the 95% credible intervals and the dark lines are the Kaplan–Meier survival curves from the data.

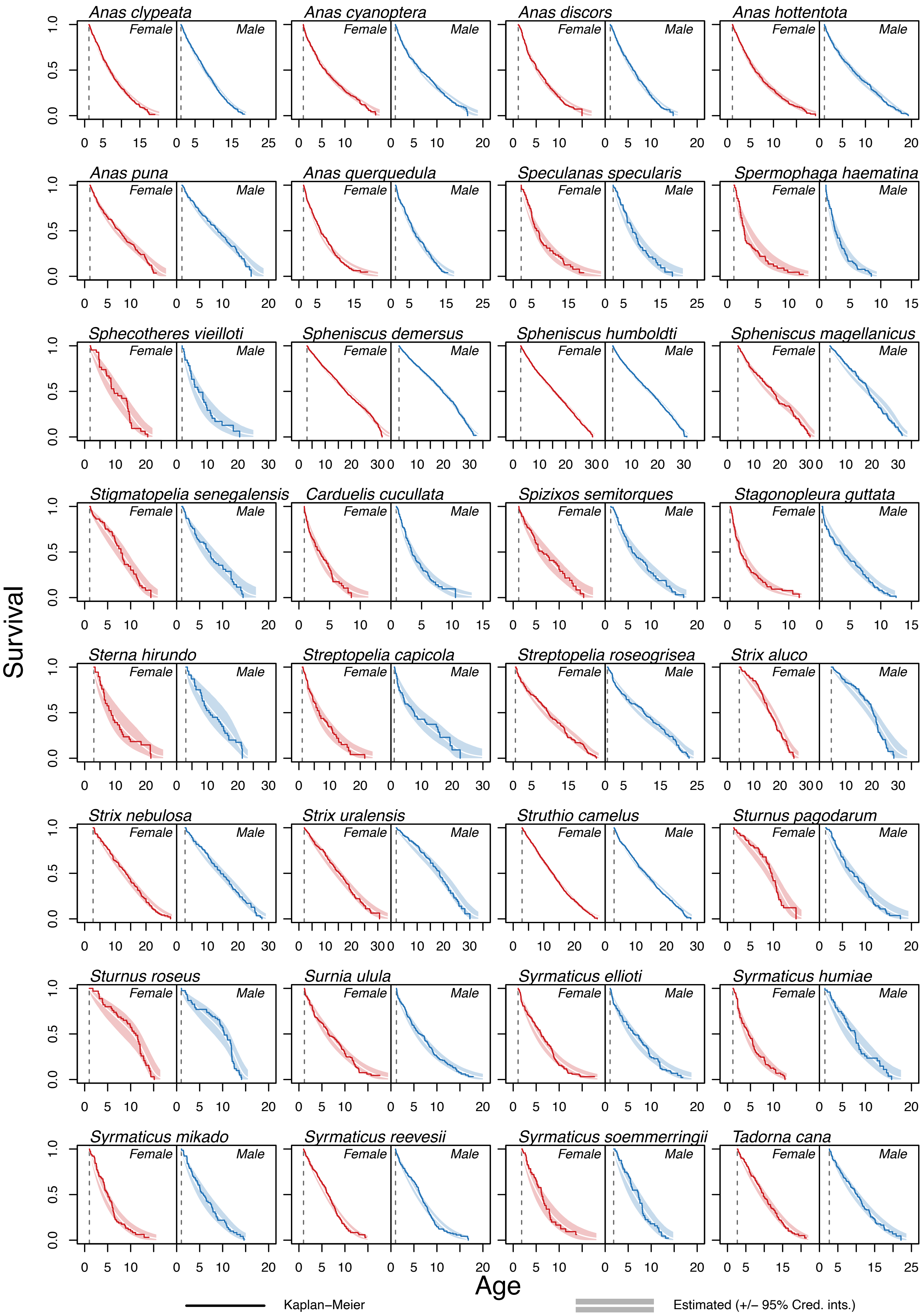

Fig. S8R. Goodness of fit plots for females and males from the Bayesian survival trajectory analysis (BaSTA) for birds.

The red and blue polygons show the estimated survival from BaSTA with the 95% credible intervals and the dark lines are the Kaplan–Meier survival curves from the data.

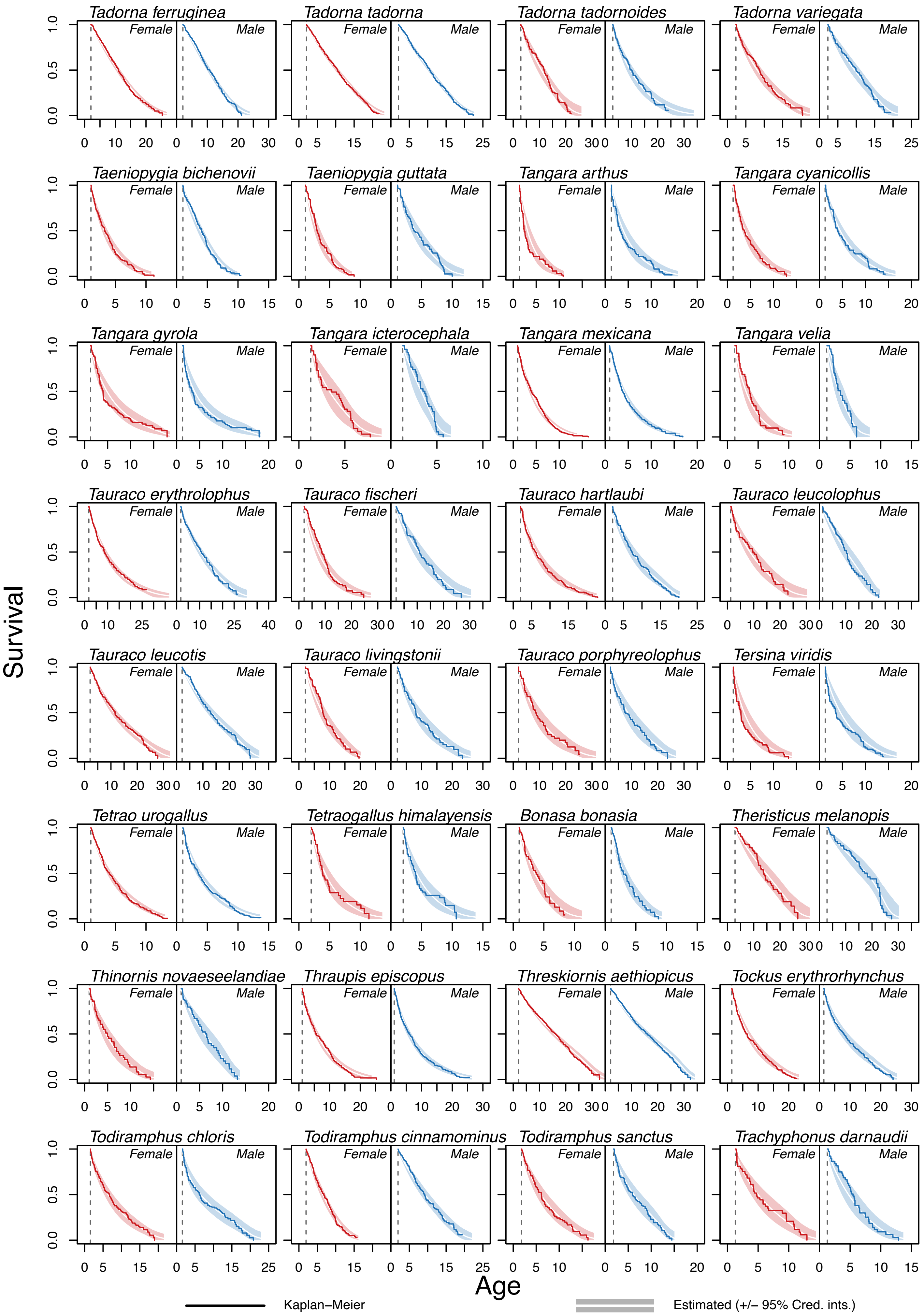

Fig. S8S. Goodness of fit plots for females and males from the Bayesian survival trajectory analysis (BaSTA) for birds.

The red and blue polygons show the estimated survival from BaSTA with the 95% credible intervals and the dark lines are the Kaplan–Meier survival curves from the data.

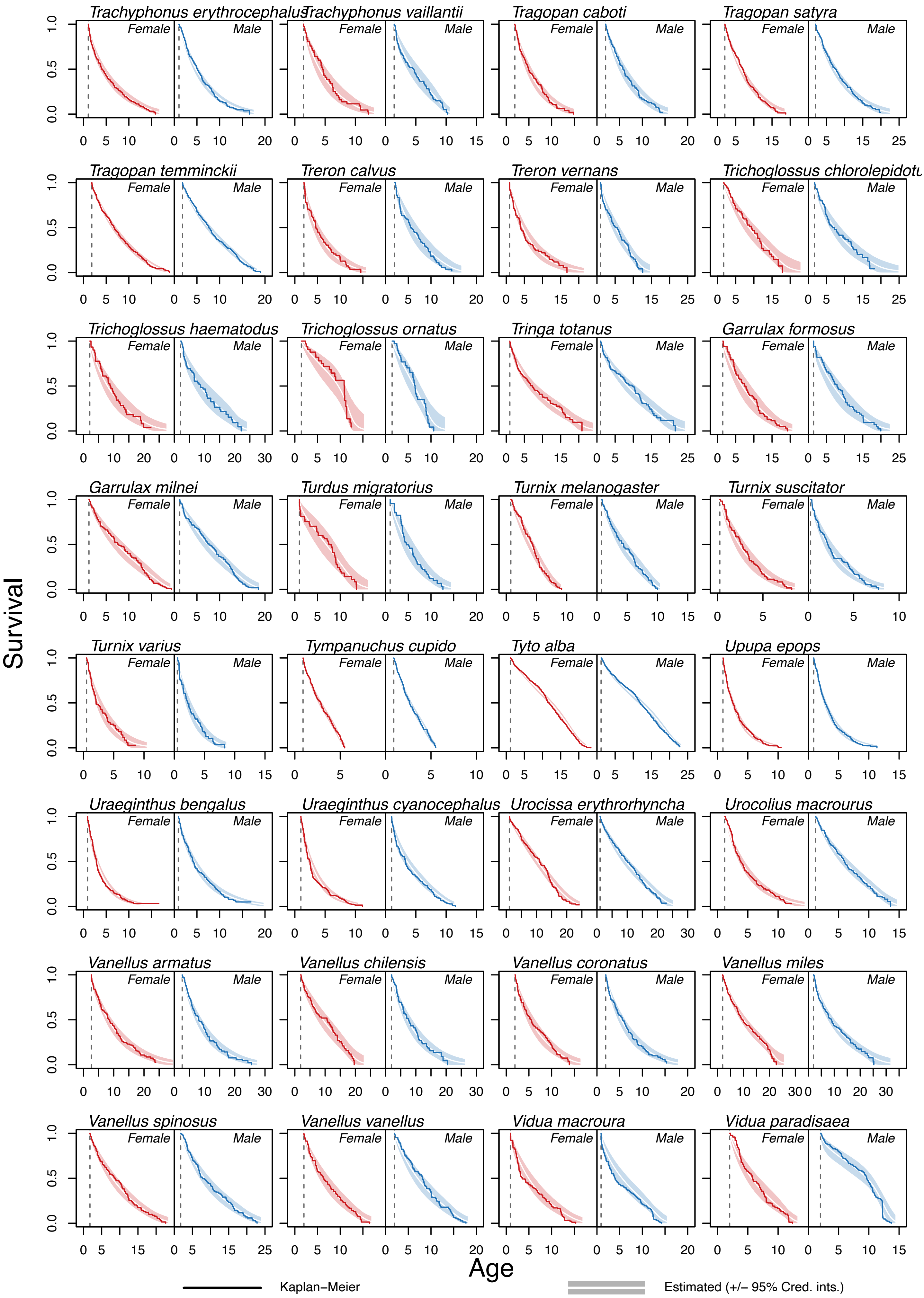

Fig. S8T. Goodness of fit plots for females and males from the Bayesian survival trajectory analysis (BaSTA) for birds.

The red and blue polygons show the estimated survival from BaSTA with the 95% credible intervals and the dark lines are the Kaplan–Meier survival curves from the data.

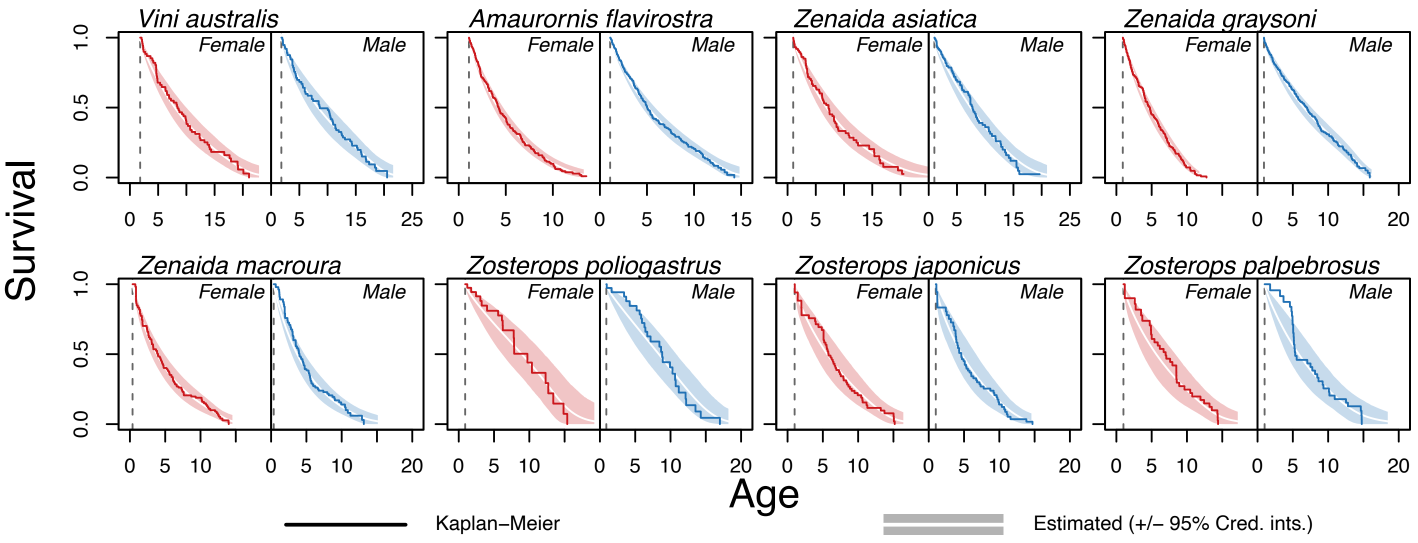

Fig. S8U. Goodness of fit plots for females and males from the Bayesian survival trajectory analysis (BaSTA) for birds.

The red and blue polygons show the estimated survival from BaSTA with the 95% credible intervals and the dark lines are the Kaplan–Meier survival curves from the data.

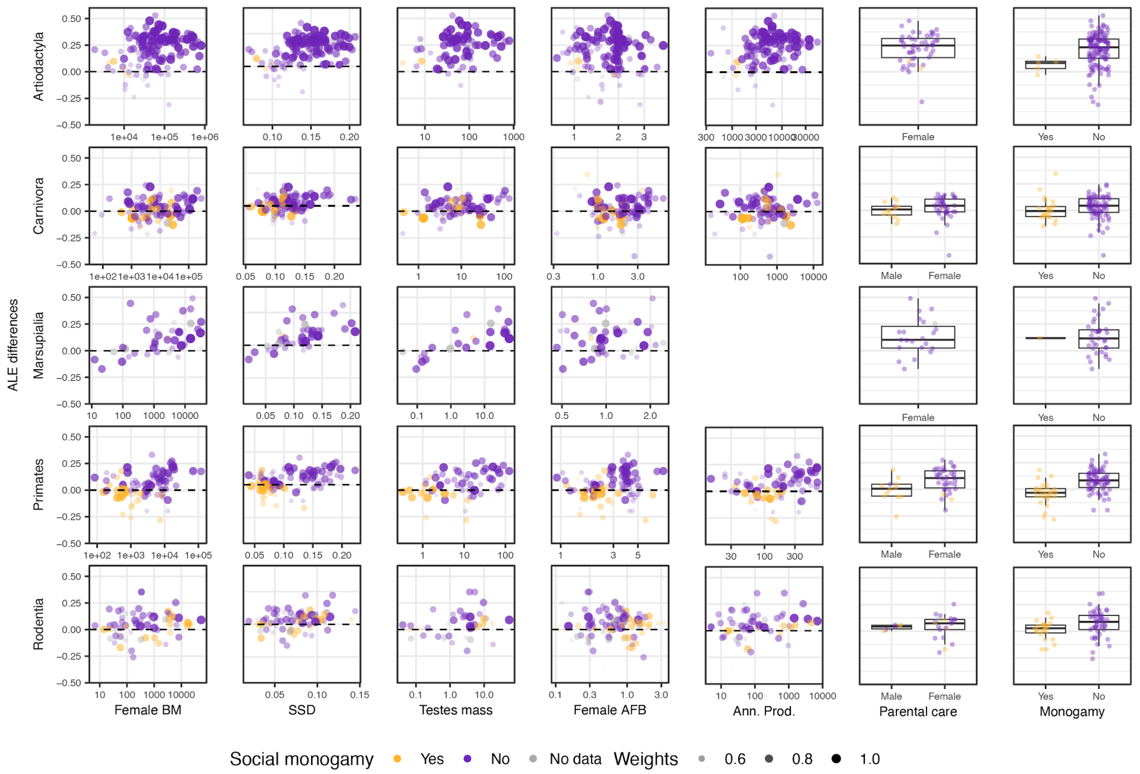

Fig. S9. Life history traits vs. Adult Life Expectancy differences for mammalian orders with a minimum sample size of 30 species. BM = Body mass in grams, SSD = Sexual size dimorphism, calculated as log(Male mass) - log(Female mass), Testes mass = Testes mass in grams, AFB = age at first birth in years, Ann. Prod. = Annual productivity, refers to annual litter/clutch mass in grams. Weights refer to PGLS weights (not shown in boxplots). Marsupialia includes the orders Dasyuromorphia, Peramelemorphia, Diprotodontia, and Didelphimorphia.

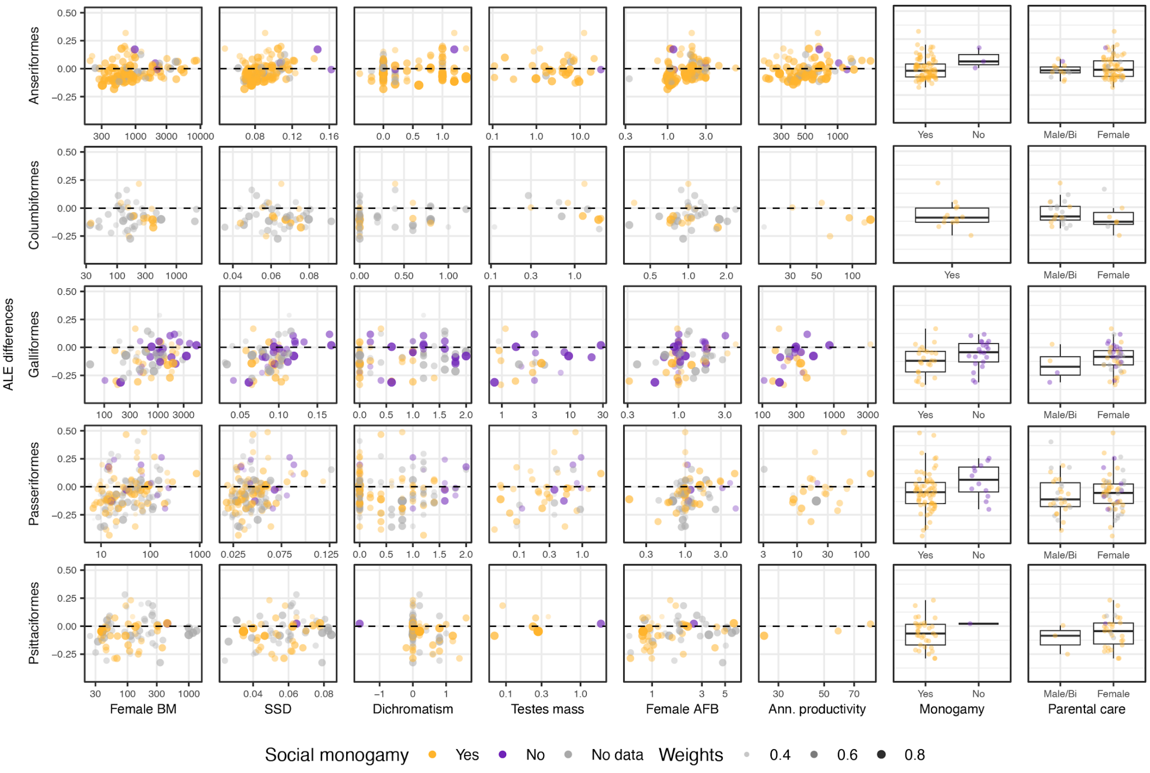

Fig. S10. Life history traits *vs.* Adult Life Expectancy differences for bird orders with a minimum sample size of 30 species. BM = Body mass in grams, SSD = Sexual size dimorphism calculated as log(Male mass) - log(Female mass), Testes mass = Testes mass in grams, AFB = age at first birth in years, Ann. Productivity. = Annual productivity, refers to annual clutch mass in grams, MS = Mating system. Weights refer to PGLS weights (see Supplementary Methods) but are not shown in the boxplots.

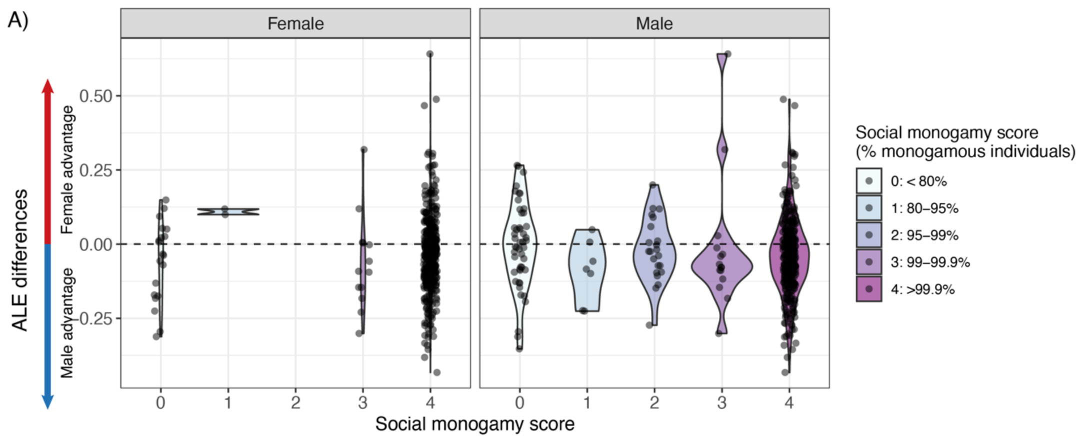

Fig. S11. Mating system *vs.* Adult Life Expectancy (ALE) differences in birds. A) Mating system in birds was scored from 0-4 in both sexes, depending on the percentage of individuals being socially monogamous within a species following Székely et al. (2022). For the analysis in this study, we used a combined measure for males and females, where monogamous refers to more than 80% of individuals being monogamous in both sexes (scores 1-4), and non-monogamous (including polyandrous, polygynous, promiscuous, and polygynandrous species) when less than 80% were monogamous in either sex or both sexes (score 0).

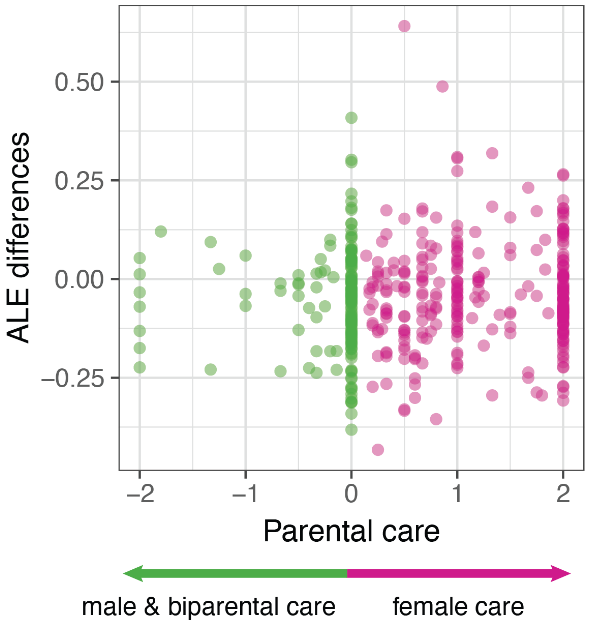

Fig S12. Parental care *vs.* Adult Life Expectancy (ALE) differences in birds. Parental care in birds was measured as the female contribution to care compared to the males, based on mean values across eight original parental care behaviors following Székely et al. (2022) resulting in a continuous scale in the interval [-2, 2], where -2 is male-only care and 2 is female-only care. For comparability with mammals, we transformed these values into two categories: male/biparental care (for values ≤ 0, green points) and female care (for values > 0, pink points).

Table S1. Weighted mean Adult Life Expectancy (ALE) differences and weighted standard errors and sample size per order for mammals and birds in zoo and in the wild. ALE differences are expressed as percentages, where positive values indicate a female ALE advantage, and negative values indicate a male ALE advantage. Results are shown for all data and for species with sex differences with zero overlap ≤ 0.05 (marked with *). † marks marsupial orders, grouped as the superorder Australidelphia and the order Didelphimorphia in Fig. 1 ‡ marks orders grouped as the superorder Afrotheria in Fig. 1.

Mammalia									
		**Zoo**				**Wild**			
Order	**Common names**	**N**	**Mean (SE)**	**N***	**Mean (SE)***	**N**	**Mean (SE)**	**N***	**Mean (SE)***
Artiodactyla	Camels, pigs, ruminants	136	24.5 (1.1)	100	27.9 (1.0)	25	20.1 (5.2)	14	27.2 (7.2)
Peramelemorphia†	Bandicoots, bilbies	2	21.4 (7.9)	2	21.4 (7.9)	-	-	-	-
Perissodactyla	Horses, zebras, rhinos, tapirs	11	18.3 (4.5)	7	24.3 (3.8)	4	-1.2 (4.9)	-	-
Scandentia	Treeshrews	4	16.6 (4.1)	3	18.7 (2.9)	1	43.2	1	43.2
Didelphimorphia†	Opossums	3	16.3 (10.0)	1	33.9	-	-	-	-
Chiroptera	Bats	18	13.8 (4.0)	10	22.3 (5.4)	-	-	-	-
Diprotodontia†	Kangaroos, wallabies, possums, koalas, wombats	32	12.9 (2.4)	16	16.2 (3.5)	1	28.0	-	-
Hyracoidea‡	Hyraxes	2	8.9 (1.6)	1	9.7	1	58.2	1	58.2
Dasyuromorphia†	Quolls, dunnarts, numbat, Tasmanian devil	9	6.3 (5.2)	5	9.3 (8.8)	-	-	-	-
Primates	Monkeys, apes, lemurs, lorisids, bush babies	99	6.2 (1.1)	29	12.9 (2.4)	7	24.0 (4.6)	4	25.9 (6.1)
Rodentia	Rodents	86	5.3 (1.2)	22	11.0 (3.0)	6	11.3 (9.1)	2	15.3 (25.1)
Cingulata	Armadillos	2	3.5 (3.4)	-	-	-	-	-	-
Carnivora	Dog-likes & cat-likes	102	3.1 (1.0)	33	6.2 (2.1)	21	19.3 (5.4)	9	26.3 (8.8)
Erinaceomorpha	Hedgehogs	3	2.7 (2.9)	-	-	1	18.9	1	18.9
Tubulidentata‡	Aardvark	1	2.4	-	-	-	-	-	-
Lagomorpha	Rabbits, hares	7	0.6 (6.5)	2	3.6 (17.6)	2	5.1 (5.0)	1	7.0
Soricomorpha	Asian house shrew	1	0.3	-	-	-	-	-	-
Afrosoricida‡	Tenrecs	3	-2.8 (8.8)	2	-0.5 (13.5)	-	-	-	-
Pilosa	Anteaters, sloths	3	-8.7 (2.7)	1	-12.6	-	-	-	-
Macroscelidea‡	Elephant shrews	3	-9.0 (4.4)	1	-10.1	-	-	-	-
Aves									
Otidiformes	Kori bustard	1	17.1	-	-	-	-	-	-
Apodiformes	Hummingbirds	3	16.6 (4.9)	1	26.5	-	-	-	-
Trogoniformes	Golden-headed quetzal	1	15.7	-	-	-	-	-	-
Accipitriformes	Birds of prey	6	15.5 (9.0)	2	30.0 (24.9)	-	-	-	-
Struthioniformes	Common ostrich	1	5.9	-	-	-	-	-	-
Cariamiformes	Red-legged seriema	1	3.8	-	-	-	-	-	-
Charadriiformes	Waders, auks, gulls	27	-1.1 (1.7)	1	10.9	2	-1.5 (11.8)	-	-
Suliformes	Gannets, cormorants	3	-1.2 (4.8)	-	-	-	-	-	-
Strigiformes	Owls	20	-1.4 (2.4)	7	-1.5 (5.5)	4	1.1 (13.9)	1	26.8
Rheiformes	Rheas	2	-2.0 (2.1)	-	-	-	-	-	-
Sphenisciformes	Penguins	8	-2.3 (2.1)	2	0.4 (10.4)	2	-25.3 (4.0)	1	-27.0

Anseriformes	Ducks, geese, swans, magpie goose	106	-2.8 (0.8)	22	-6.4 (2.3)	17	-29.9 (3.5)	14	-33.3 (2.8)
Falconiformes	Falcons	5	-4.4 (4.1)	-	-	2	-5.0 (5.3)	-	-
Pelecaniformes	Herons, pelicans, ibises, spoonbills, hamerkop	28	-4.8 (2.1)	5	-19.1 (8.5)	-	-	-	-
Musophagiformes	Turacos	10	-4.9 (3.1)	1	-27.7	-	-	-	-
Psittaciformes	Parrots	73	-5.1 (1.4)	19	-12.8 (3.0)	1	-67.0	1	-67.0
Passeriformes	Songbirds	151	-5.4 (1.3)	36	-13.0 (3.5)	5	-7.3 (7.1)	1	-40.5
Gruiformes	Crane-likes	19	-6.3 (3.0)	4	-13.9 (12.6)	1	-2.7	-	-
Casuariiformes	Emu	1	-7.0	-	-	-	-	-	-
Coraciiformes	Kingfishers, rollers, bee-eaters, motmots	13	-7.4 (2.5)	2	-18.7 (4.3)	-	-	-	-
Piciformes	Toucans	23	-8.5 (1.9)	3	-21.8 (2.5)	-	-	-	-
Columbiformes	Doves, pigeons	48	-8.8 (1.3)	11	-15.8 (1.8)	1	-25.8	1	-25.8
Galliformes	Pheasants, chicken, peafowl, etc.	74	-9.1 (1.4)	21	-17.4 (2.3)	4	-13.9 (10.4)	1	-54.0
Ciconiiformes	Storks	5	-10.7 (8.3)	2	-27.5 (6.7)	1	29.4	-	-
Bucerotiformes	Hornbills, hoopoes	6	-10.8 (3.5)	1	-20.0	-	-	-	-
Phoenicopteriformes	Flamingos	3	-12.8 (5.4)	2	-17.9 (4.5)	-	-	-	-
Cuculiformes	Cuckoos	3	-17.0 (4.9)	1	-23.0	1	29.1		-
Tinamiformes	Tinamous	3	-18.0 (2.1)	1	-17.5	-	-	-	-
Caprimulgiformes	Nightjar	1	-18.3	1	-18.3	-	-	-	-
Coliiformes	Mousebirds	2	-18.8 (6.3)	2	-18.8 (6.3)	-	-	-	-
Eurypygiformes	Sunbittern	1	-23.2	1	-23.2	-	-	-	-

Table S2. Parameter estimates of the models fitted using Bayesian PGLS to account for variation observed in Adult Life Expectancy (ALE) differences for mammals and birds. Results show all tested models for the three tested hypotheses relating to pre- and post-copulatory sexual selection and the cost of reproduction. Note, given that that plumage dichromatism was only available in birds, we tested the pre-copulatory sexual selection hypothesis separately in this class. Columns show the deviance information criterion (DIC), the *Δ*DIC, calculated as the differences in DIC with respect to the lowest DIC model, sample sizes (N), posterior means and standard deviations (SD), and lower and upper 95% credible intervals from the posterior densities of the regression parameters. Zero overlap provides a two-sided test that indicates the area under the posterior density below or above 0.

Variable	DIC	ΔDIC	Mean	SD	Lower CI	Upper CI	Zero overlap
**Pre-copulatory sexual selection (n = 456 mammals, 392 birds)**							
Intercept	-2756.4	0	-0.329	0.075	-0.476	-0.184	**<0.001**
ClassMammalia			0.587	0.091	0.411	0.765	**<0.001**
Log(Male body mass)			0.106	0.024	0.059	0.155	**<0.001**
Log(Female body mass)			-0.095	0.025	-0.145	-0.046	**<0.001**
Monogamy			-0.063	0.015	-0.092	-0.034	**<0.001**
Residual variance σ^2			0.029	0.004	0.022	0.039	-
Pagel's λ			0.770	0.043	0.675	0.843	-
Intercept	-2754.5	1.9	-0.331	0.076	-0.479	-0.184	**<0.001**
ClassMammalia			0.591	0.091	0.414	0.770	**<0.001**
Log(Male body mass)			0.106	0.024	0.058	0.154	**<0.001**
Log(Female body mass)			-0.095	0.025	-0.144	-0.046	**<0.001**
ClassAves:Monogamy			-0.061	0.021	-0.103	-0.019	**0.004**
ClassMammalia:Monogamy			-0.065	0.020	-0.104	-0.025	**0.001**
Residual variance σ^2			0.029	0.004	0.022	0.039	-
Pagel's λ			0.770	0.043	0.677	0.845	-
Intercept	-2753.8	2.6	-0.373	0.087	-0.543	-0.203	**<0.001**
ClassMammalia			0.649	0.112	0.428	0.867	**<0.001**
Monogamy			-0.063	0.015	-0.092	-0.034	**<0.001**
ClassAves: Log(Male body mass)			0.073	0.048	-0.022	0.169	0.129
ClassMammalia:Log(Male body mass)			0.117	0.028	0.062	0.169	**<0.001**
ClassAves:Log(Female body mass)			-0.056	0.050	-0.155	0.043	0.265
ClassMammalia:Log(Female body mass)			-0.107	0.029	-0.162	-0.051	**<0.001**
Residual variance σ^2			0.029	0.004	0.022	0.039	-
Pagel's λ			0.769	0.044	0.673	0.845	-
Intercept	-2751.9	4.5	-0.374	0.088	-0.547	-0.205	**<0.001**
ClassMammalia			0.650	0.112	0.431	0.869	**<0.001**
ClassAves: Log(Male body mass)			0.074	0.048	-0.021	0.170	0.124
ClassMammalia: Log(Male body mass)			0.116	0.028	0.062	0.171	**<0.001**
ClassAves:Log(Female body mass)			-0.057	0.050	-0.157	0.041	0.256
ClassMammalia: Log(Female body mass)			-0.107	0.029	-0.163	-0.051	**<0.001**
ClassAves:Monogamy			-0.062	0.022	-0.104	-0.019	**0.004**
ClassMammalia:Monogamy			-0.064	0.020	-0.103	-0.025	**0.001**
Residual variance σ^2			0.029	0.004	0.022	0.039	-
Pagel's λ			0.770	0.043	0.673	0.844	-
Intercept	-2715.9	40.5	0.008	0.054	-0.097	0.116	0.881
Log(Male body mass)			0.113	0.025	0.065	0.162	**<0.001**
Log(Female body mass)			-0.099	0.026	-0.149	-0.049	**<0.001**
Monogamy			-0.073	0.015	-0.102	-0.044	**<0.001**
Residual variance σ^2			0.029	0.005	0.021	0.039	-
Pagel's λ			0.747	0.052	0.634	0.834	-
Intercept	-2694.5	61.9	-0.289	0.073	-0.432	-0.145	**<0.001**
ClassMammalia			0.732	0.092	0.550	0.912	**<0.001**
Residual variance σ^2			0.035	0.005	0.026	0.046	-
Pagel's λ			0.799	0.039	0.714	0.865	-
**Pre-copulatory sexual selection including plumage dichromatism (n = 392 birds)**							

Intercept	-1228.1	0.0	-0.176	0.069	-0.311	-0.043	**0.010**
Log(Male body mass)			0.053	0.052	-0.05	0.153	0.306
Log(Female body mass)			-0.036	0.053	-0.14	0.069	0.495
Monogamy			-0.064	0.025	-0.113	-0.014	**0.012**
Plumage dichromatism			0.005	0.014	-0.021	0.032	0.700
Residual variance σ^2			0.014	0.002	0.011	0.018	-
Pagel's λ			0.437	0.09	0.254	0.607	-
Intercept	-1224.4	3.7	-0.034	0.04	-0.114	0.041	0.395
Monogamy			-0.083	0.024	-0.13	-0.037	**<0.001**
Plumage dichromatism			-0.003	0.013	-0.029	0.023	0.827
Residual variance σ^2			0.014	0.002	0.011	0.018	-
Pagel's λ			0.401	0.095	0.209	0.58	-
Intercept	-1223.2	4.9	-0.244	0.064	-0.374	-0.125	**<0.001**
Log(Male body mass)			0.093	0.049	-0.004	0.191	0.056
Log(Female body mass)			-0.075	0.051	-0.176	0.026	0.142
Plumage dichromatism			0.016	0.013	-0.01	0.041	0.227
Residual variance σ^2			0.015	0.002	0.012	0.019	-
Pagel's λ			0.451	0.091	0.266	0.619	-
**Post-copulatory sexual selection (n = 278 mammals, 137 birds)**							
Intercept	-1341.4	0	-0.398	0.117	-0.631	-0.171	**0.001**
ClassMammalia			0.558	0.130	0.303	0.812	**<0.001**
Log(Male body mass)			0.009	0.006	-0.003	0.021	0.134
Log(Testes mass)			0.011	0.007	-0.002	0.024	0.102
Monogamy			-0.053	0.020	-0.092	-0.013	**0.009**
Residual variance σ^2			0.037	0.008	0.025	0.055	-
Pagel's λ			0.827	0.050	0.713	0.906	-
Intercept	-1340.3	1.1	-0.408	0.117	-0.636	-0.179	**0.001**
ClassMammalia			0.567	0.129	0.314	0.818	**<0.001**
Log(Male body mass)			0.009	0.006	-0.003	0.021	0.147
Log(Testes mass)			0.011	0.007	-0.002	0.024	0.101
ClassAves:Monogamy			-0.031	0.031	-0.093	0.030	0.315
ClassMammalia:Monogamy			-0.068	0.026	-0.119	-0.016	**0.010**
Residual variance σ^2			0.038	0.008	0.025	0.055	-
Pagel's λ			0.828	0.049	0.713	0.906	-
Intercept	-1339.9	1.5	-0.515	0.145	-0.800	-0.235	**<0.001**
ClassMammalia			0.720	0.172	0.388	1.060	**<0.001**
Monogamy			-0.052	0.020	-0.093	-0.013	**0.01**
ClassAves:Log(Male body mass)			0.025	0.012	0.001	0.049	**0.041**
ClassMammalia:Log(Male body mass)			0.003	0.007	-0.011	0.017	0.678
ClassAves:Log(Testes mass)			0.002	0.011	-0.019	0.022	0.870
ClassMammalia:Log(Testes mass)			0.017	0.009	0.001	0.034	**0.049**
Residual variance σ^2			0.038	0.008	0.025	0.055	-
Pagel's λ			0.831	0.048	0.722	0.908	-
Intercept	-1338.7	2.7	-0.533	0.147	-0.825	-0.248	**<0.001**
ClassMammalia			0.735	0.172	0.401	1.076	**<0.001**
ClassAves:Log(Male body mass)			0.025	0.012	0.001	0.049	**0.039**
ClassMammalia:Log(Male body mass)			0.003	0.007	-0.011	0.017	0.668
ClassAves:Log(Testes mass)			0.003	0.011	-0.018	0.024	0.775
ClassMammalia:Log(Testes mass)			0.016	0.009	0.000	0.033	0.056
ClassAves:Monogamy			-0.030	0.032	-0.093	0.032	0.342
ClassMammalia:Monogamy			-0.067	0.026	-0.118	-0.015	**0.011**
Residual variance σ^2			0.038	0.008	0.025	0.056	-
Pagel's λ			0.830	0.049	0.719	0.907	-
Intercept	-1324	17.4	-0.020	0.076	-0.170	0.126	0.793
Log(Male body mass)			0.011	0.006	-0.001	0.022	0.075
Log(Testes mass)			0.011	0.007	-0.003	0.024	0.117
Monogamy			-0.062	0.021	-0.102	-0.022	**0.003**
Residual variance σ^2			0.035	0.008	0.022	0.053	-
Pagel's λ			0.792	0.064	0.644	0.894	-
**Cost of Reproduction (n = 183 mammals, 217 birds)**							
Intercept	-1359.7	0	-0.325	0.094	-0.507	-0.143	**0.001**
ClassMammalia			0.540	0.115	0.312	0.770	**<0.001**

Log(Female body mass)			0.001	0.009	-0.017	0.018	0.934
Log(Female age at first birth)			0.024	0.015	-0.005	0.053	0.108
Log(Annual productivity)			0.006	0.011	-0.015	0.028	0.56
Female parental care			0.056	0.016	0.023	0.088	**0.001**
Residual variance σ^2			0.033	0.006	0.022	0.046	-
Pagel's λ			0.837	0.044	0.737	0.907	-
Intercept	-1358	1.7	-0.355	0.114	-0.58	-0.129	**0.002**
ClassMammalia			0.584	0.147	0.298	0.877	**<0.001**
Log(Female age at first birth)			0.023	0.015	-0.006	0.053	0.121
Log(Annual productivity)			0.007	0.011	-0.015	0.029	0.546
Female parental care			0.057	0.016	0.025	0.09	**0.001**
ClassAves:Log(Female body mass)			0.004	0.011	-0.018	0.025	0.731
ClassMammalia:Log(Female body mass)			-0.001	0.010	-0.019	0.018	0.944
Residual variance σ^2			0.033	0.006	0.022	0.047	-
Pagel's λ			0.839	0.042	0.743	0.907	-
Intercept	-1357.9	1.8	-0.317	0.116	-0.542	-0.092	**0.006**
ClassMammalia			0.516	0.154	0.214	0.816	**0.001**
Log(Annual productivity)			0.004	0.011	-0.018	0.026	0.708
Female parental care			0.056	0.016	0.024	0.089	**0.001**
ClassAves:Log(Female body mass)			0.001	0.011	-0.021	0.023	0.941
ClassMammalia:Log(Female body mass)			0.005	0.011	-0.015	0.026	0.607
ClassAves:Log(Female age at first birth)			0.041	0.020	0.002	0.080	**0.04**
ClassMammalia:Log(Female age at first birth)			0.002	0.022	-0.041	0.044	0.936
Residual variance σ^2			0.033	0.006	0.022	0.047	-
Pagel's λ			0.836	0.044	0.736	0.907	-
Intercept	-1355.8	3.9	-0.318	0.120	-0.551	-0.082	**0.008**
ClassMammalia			0.517	0.159	0.206	0.831	**0.001**
Female parental care			0.056	0.017	0.024	0.089	**0.001**
ClassAves:Log(Female body mass)			0.001	0.014	-0.027	0.030	0.936
ClassMammalia:Log(Female body mass)			0.005	0.012	-0.018	0.028	0.668
ClassAves:Log(Female age at first birth)			0.040	0.020	0.001	0.079	**0.043**
ClassMammalia:Log(Female age at first birth)			0.002	0.023	-0.043	0.046	0.94
ClassAves:Log(Annual productivity)			0.004	0.020	-0.035	0.043	0.852
ClassMammalia:Log(Annual productivity)			0.005	0.014	-0.022	0.032	0.734
Residual variance σ^2			0.033	0.006	0.022	0.047	-
Pagel's λ			0.837	0.044	0.736	0.907	-
Intercept	-1354	5.7	-0.308	0.122	-0.547	-0.071	**0.011**
ClassMammalia			0.502	0.162	0.187	0.824	**0.002**
ClassAves:Log(Female body mass)			0.001	0.014	-0.027	0.029	0.933
ClassMammalia:Log(Female body mass)			0.004	0.012	-0.020	0.028	0.742
ClassAves:Log(Female age at first birth)			0.041	0.020	0.002	0.079	**0.041**
ClassMammalia:Log(Female age at first birth)			0.002	0.023	-0.043	0.046	0.925
ClassAves:Log(Annual productivity)			0.003	0.020	-0.037	0.043	0.882
ClassMammalia:Log(Annual productivity)			0.005	0.014	-0.021	0.032	0.696
ClassAves:Female parental care			0.052	0.019	0.014	0.089	**0.007**
ClassMammalia:Female parental care			0.070	0.032	0.007	0.133	**0.03**
Residual variance σ^2			0.033	0.006	0.022	0.047	-
Pagel's λ			0.838	0.044	0.739	0.909	-
Intercept	-1339.4	20.3	-0.068	0.078	-0.218	0.087	0.381
Log(Female body mass)			0.003	0.009	-0.015	0.021	0.755
Log(Female age at first birth)			0.023	0.015	-0.007	0.054	0.127
Log(Annual productivity)			0.007	0.011	-0.015	0.029	0.528
Female parental care			0.056	0.017	0.024	0.089	**0.001**
Residual variance σ^2			0.032	0.007	0.021	0.047	-
Pagel's λ			0.820	0.051	0.702	0.901	-

Table S3. Parameter estimates of the models fitted using Bayesian PGLS to test the sexual selection hypotheses on Adult Life Expectancy (ALE) differences for the wild dataset. Columns show the deviance information criterion (DIC), the *Δ*DIC, calculated as the differences in DIC with respect to the lowest DIC model, sample sizes (N), posterior means and standard deviations (SD), and lower and upper 95% credible intervals from the posterior densities of the regression parameters. Zero overlap provides a two-sided test that indicates the area under the posterior density below or above 0.

Variable	DIC	ΔDIC	Mean	SD	Lower CI	Upper CI	Zero overlap
**Pre-copulatory sexual selection (n = 64 mammals, 41 birds)**							
Intercept	-193.2	0.0	-0.292	0.227	-0.750	0.140	0.198
ClassMammalia			0.493	0.260	0.008	1.033	0.058
Log(Male body mass)			0.101	0.107	-0.104	0.311	0.343
Log(Female body mass)			-0.084	0.112	-0.302	0.132	0.450
Monogamy			-0.129	0.100	-0.324	0.072	0.199
Residual variance σ^2			0.047	0.015	0.027	0.083	-
Pagel's λ			0.542	0.160	0.187	0.800	-
Intercept	-193.1	0.2	-0.297	0.161	-0.637	0.003	0.065
ClassMammalia			0.677	0.235	0.270	1.181	**0.004**
Residual variance σ^2			0.044	0.014	0.026	0.078	-
Pagel's λ			0.485	0.167	0.128	0.772	-
Intercept	-191.2	2.0	-0.294	0.258	-0.827	0.200	0.255
ClassMammalia			0.496	0.285	-0.051	1.079	0.082
Log(Male body mass)			0.102	0.108	-0.111	0.312	0.346
Log(Female body mass)			-0.084	0.113	-0.304	0.139	0.454
ClassAves:Monogamy			-0.127	0.165	-0.456	0.193	0.440
ClassMammalia:Monogamy			-0.131	0.125	-0.373	0.113	0.295
Residual variance σ^2			0.048	0.016	0.027	0.087	-
Pagel's λ			0.546	0.160	0.199	0.813	-
Intercept	-191	2.2	-0.017	0.168	-0.350	0.307	0.918
Log(Male body mass)			0.084	0.110	-0.129	0.302	0.442
Log(Female body mass)			-0.063	0.114	-0.290	0.160	0.583
Monogamy			-0.212	0.092	-0.390	-0.032	**0.021**
Residual variance σ^2			0.046	0.015	0.026	0.082	-
Pagel's λ			0.507	0.170	0.145	0.788	-
Intercept	-189.3	3.9	-0.313	0.295	-0.908	0.246	0.288
ClassMammalia			0.516	0.369	-0.188	1.268	0.161
Monogamy			-0.120	0.105	-0.324	0.086	0.252
ClassAves:Log(Male body mass)			0.202	0.281	-0.341	0.757	0.471
ClassMammalia:Log(Male body mass)			0.084	0.119	-0.15	0.319	0.480
ClassAves:Log(Female body mass)			-0.188	0.293	-0.767	0.375	0.520
ClassMammalia:Log(Female body mass)			-0.066	0.125	-0.313	0.181	0.598
Residual variance σ^2			0.050	0.017	0.027	0.092	-
Pagel's λ			0.565	0.162	0.201	0.826	-
Intercept	-187.3	5.9	-0.339	0.340	-1.022	0.303	0.319
ClassMammalia			0.547	0.410	-0.231	1.376	0.182
ClassAves:Log(Male body mass)			0.218	0.303	-0.382	0.810	0.472
ClassMammalia:Log(Male body mass)			0.081	0.120	-0.156	0.317	0.501
ClassAves:Log(Female body mass)			-0.204	0.315	-0.817	0.414	0.517
ClassMammalia:Log(Female body mass)			-0.063	0.126	-0.310	0.185	0.618
ClassAves:Monogamy			-0.097	0.183	-0.452	0.265	0.595
ClassMammalia:Monogamy			-0.133	0.128	-0.383	0.115	0.298
Residual variance σ^2			0.051	0.017	0.028	0.092	-
Pagel's λ			0.569	0.159	0.209	0.824	-
**Pre-copulatory sexual selection including plumage dichromatism (n = 40 birds)**							
Intercept	-82.5	0.0	0.219	0.189	-0.141	0.602	0.248
Monogamy			-0.317	0.175	-0.665	0.023	0.070
Plumage dichromatism			-0.187	0.063	-0.310	-0.062	**0.003**
Residual variance σ^2			0.037	0.013	0.020	0.068	-
Pagel's λ			0.612	0.190	0.166	0.897	-

Intercept	-79.7	2.8	0.220	0.321	-0.398	0.872	0.492
Log(Male body mass)			0.306	0.274	-0.238	0.846	0.264
Log(Female body mass)			-0.321	0.289	-0.889	0.251	0.267
Monogamy			-0.255	0.191	-0.640	0.119	0.184
Plumage dichromatism			-0.195	0.066	-0.326	-0.064	0.003
Residual variance σ^2			0.040	0.014	0.021	0.074	-
Pagel's λ			0.656	0.182	0.214	0.914	-
Intercept	-79.6	2.9	-0.038	0.255	-0.537	0.460	0.880
Log(Male body mass)			0.420	0.263	-0.095	0.940	0.110
Log(Female body mass)			-0.432	0.279	-0.986	0.120	0.122
Plumage dichromatism			-0.182	0.067	-0.314	-0.050	**0.007**
Residual variance σ^2			0.039	0.014	0.021	0.073	-
Pagel's λ			0.614	0.195	0.160	0.907	-
**Post-copulatory sexual selection (n = 52 mammals, 29 birds)**							
Intercept	-149.8	0.0	-0.266	0.242	-0.746	0.203	0.272
ClassMammalia			0.406	0.271	-0.099	0.969	0.135
Log(Male body mass)			0.032	0.021	-0.01	0.073	0.134
Log(Testes mass)			-0.005	0.023	-0.049	0.039	0.825
Monogamy			-0.137	0.100	-0.335	0.061	0.171
Residual variance σ^2			0.048	0.017	0.026	0.090	-
Pagel's λ			0.568	0.164	0.199	0.834	-
Intercept	-149.2	0.6	-0.067	0.200	-0.453	0.335	0.739
Log(Male body mass)			0.037	0.021	-0.004	0.078	0.076
Log(Testes mass)			-0.007	0.023	-0.052	0.038	0.766
Monogamy			-0.193	0.095	-0.378	-0.005	**0.041**
Residual variance σ^2			0.047	0.017	0.026	0.089	-
Pagel's λ			0.541	0.175	0.153	0.826	-
Intercept	-148.0	1.8	-0.229	0.326	-0.888	0.400	0.483
ClassMammalia			0.240	0.439	-0.594	1.131	0.585
Monogamy			-0.142	0.101	-0.339	0.053	0.158
ClassAves:Log(Male body mass)			0.022	0.040	-0.055	0.102	0.573
ClassMammalia:Log(Male body mass)			0.059	0.030	-0.001	0.119	0.053
ClassAves:Log(Testes mass)			0.016	0.027	-0.038	0.068	0.560
ClassMammalia:Log(Testes mass)			-0.051	0.041	-0.131	0.031	0.218
Residual variance σ^2			0.049	0.017	0.026	0.091	-
Pagel's λ			0.573	0.165	0.195	0.829	-
Intercept	-147.7	2.1	-0.262	0.277	-0.806	0.291	0.345
ClassMammalia			0.407	0.300	-0.173	1.013	0.175
Log(Male body mass)			0.031	0.021	-0.009	0.072	0.138
Log(Testes mass)			-0.004	0.023	-0.049	0.040	0.844
ClassAves:Monogamy			-0.140	0.165	-0.466	0.186	0.397
ClassMammalia:Monogamy			-0.136	0.127	-0.386	0.111	0.284
Residual variance σ^2			0.050	0.017	0.026	0.093	-
Pagel's λ			0.577	0.164	0.206	0.840	-
Intercept	-145.9	3.9	-0.258	0.370	-0.982	0.461	0.486
ClassMammalia			0.271	0.477	-0.637	1.237	0.570
ClassAves:Log(Male body mass)			0.023	0.040	-0.055	0.103	0.563
ClassMammalia:log(Male body mass)			0.059	0.031	-0.001	0.120	0.053
ClassAves:Log(Testes mass)			0.016	0.028	-0.038	0.071	0.551
ClassMammalia:Log(Testes mass)			-0.051	0.041	-0.132	0.030	0.216
ClassAves:Monogamy			-0.119	0.171	-0.458	0.218	0.486
ClassMammalia:Monogamy			-0.155	0.128	-0.403	0.100	0.228
Residual variance σ^2			0.050	0.018	0.026	0.095	-
Pagel's λ			0.581	0.166	0.197	0.840	-
**Cost of reproduction (n = 48 mammals, 38 birds)**							
Intercept	-155.9	0.0	-0.428	0.227	-0.888	0.005	0.059
ClassMammalia			0.583	0.250	0.118	1.096	**0.020**
Log(Female body mass)			0.021	0.040	-0.056	0.100	0.589
Log(Female age at first birth)			0.027	0.068	-0.107	0.162	0.690
Log(Annual productivity)			-0.018	0.044	-0.104	0.068	0.678
Female parental care			0.139	0.091	-0.043	0.315	0.128
Residual variance σ^2			0.048	0.016	0.027	0.089	-

Pagel's λ			0.529	0.160	0.190	0.806	-
Intercept	-153.8	2.1	-0.425	0.295	-1.020	0.147	0.150
ClassMammalia			0.583	0.373	-0.142	1.336	0.119
Log(Female age at first birth)			0.029	0.069	-0.105	0.164	0.678
Log(Annual productivity)			-0.016	0.045	-0.103	0.072	0.714
Female parental care			0.137	0.091	-0.043	0.319	0.134
ClassAves:Log(Female body mass)			0.020	0.046	-0.070	0.112	0.672
ClassMammalia:Log(Female body mass)			0.021	0.043	-0.062	0.105	0.627
Residual variance σ^2			0.049	0.017	0.027	0.092	-
Pagel's λ			0.538	0.164	0.181	0.815	-
Intercept	-153.1	2.8	-0.297	0.320	-0.940	0.324	0.353
ClassMammalia			0.316	0.457	-0.563	1.243	0.489
Log(Annual productivity)			-0.035	0.048	-0.128	0.063	0.473
Female parental care			0.136	0.093	-0.045	0.316	0.141
ClassAves:Log(Female body mass)			0.014	0.046	-0.078	0.104	0.767
ClassMammalia:Log(Female body mass)			0.050	0.052	-0.053	0.151	0.332
ClassAves:Log(Female age at first birth)			0.077	0.086	-0.090	0.246	0.367
ClassMammalia:Log(Female age at first birth)			-0.048	0.104	-0.253	0.155	0.642
Residual variance σ^2			0.048	0.017	0.026	0.091	-
Pagel's λ			0.518	0.171	0.147	0.809	-
Intercept	-152.3	3.6	-0.197	0.375	-0.984	0.490	0.600
ClassMammalia			0.120	0.504	-0.829	1.173	0.811
ClassAves:Log(Female body mass)			-0.005	0.065	-0.134	0.121	0.935
ClassMammalia:Log(Female body mass)			0.045	0.058	-0.071	0.157	0.443
ClassAves:Log(Female age at first birth)			0.104	0.089	-0.071	0.279	0.245
ClassMammalia:Log(Female age at first birth)			-0.051	0.110	-0.267	0.165	0.642
ClassAves:Log(Annual productivity)			-0.009	0.097	-0.191	0.189	0.923
ClassMammalia:Log(Annual productivity)			-0.030	0.055	-0.137	0.080	0.588
ClassAves:Female parental care			-0.026	0.140	-0.298	0.247	0.854
ClassMammalia:Female parental care			0.265	0.126	0.017	0.511	**0.035**
Residual variance σ^2			0.048	0.018	0.025	0.094	-
Pagel's λ			0.516	0.185	0.106	0.816	-
Intercept	-151.7	4.2	-0.303	0.366	-1.055	0.383	0.408
ClassMammalia			0.332	0.488	-0.573	1.332	0.496
Female parental care			0.136	0.093	-0.048	0.317	0.144
ClassAves:Log(Female body mass)			0.017	0.066	-0.115	0.144	0.800
ClassMammalia:Log(Female body mass)			0.047	0.058	-0.068	0.162	0.423
ClassAves:Log(Female age at first birth)			0.075	0.088	-0.095	0.250	0.392
ClassMammalia:Log(Female age at first birth)			-0.042	0.111	-0.265	0.176	0.701
ClassAves:Log(Annual productivity)			-0.036	0.099	-0.221	0.165	0.716
ClassMammalia:Log(Annual productivity)			-0.031	0.055	-0.140	0.076	0.567
Residual variance σ^2			0.050	0.018	0.026	0.096	-
Pagel's λ			0.521	0.185	0.113	0.822	-
Intercept	-151.1	4.8	-0.256	0.221	-0.686	0.185	0.248
Log(Female body mass)			0.049	0.040	-0.030	0.129	0.225
Log(Female age at first birth)			-0.013	0.069	-0.149	0.124	0.856
Log(Annual productivity)			-0.031	0.046	-0.122	0.060	0.501
Female parental care			0.156	0.093	-0.026	0.340	0.095
Residual variance σ^2			0.053	0.018	0.029	0.099	-
Pagel's λ			0.557	0.160	0.214	0.829	-

Table S4. Parameter estimates of the models fitted using Bayesian PGLS to test the sexual selection hypotheses on Adult Life Expectancy (ALE) differences for the reduced zoo dataset. Columns show the deviance information criterion (DIC), the *Δ*DIC, calculated as the differences in DIC with respect to the lowest DIC model, sample sizes (N), posterior means and standard deviations (SD), and lower and upper 95% credible intervals from the posterior densities of the regression parameters. Zero overlap provides a two-sided test that indicates the area under the posterior density below or above 0.

Variable	DIC	ΔDIC	Mean	SD	Lower CI	Upper CI	Zero overlap
**Pre-copulatory sexual selection (n = 64 mammals, 41 birds)**							
Intercept	-347.2	0.0	-0.355	0.176	-0.708	-0.018	**0.044**
ClassMammalia			0.845	0.233	0.405	1.310	**<0.001**
Residual variance σ^2			0.050	0.015	0.026	0.085	-
Pagel's λ			0.909	0.050	0.784	0.973	-
Intercept	-346.7	0.4	-0.436	0.187	-0.813	-0.079	**0.019**
ClassMammalia			0.789	0.240	0.324	1.262	**0.001**
Log(Male body mass)			0.095	0.056	-0.013	0.206	0.088
Log(Female body mass)			-0.089	0.058	-0.203	0.023	0.126
Monogamy			-0.004	0.050	-0.101	0.094	0.929
Residual variance σ^2			0.050	0.017	0.025	0.088	-
Pagel's λ			0.908	0.056	0.766	0.978	-
Intercept	-346.4	0.7	-0.452	0.186	-0.835	-0.103	**0.015**
ClassMammalia			0.808	0.239	0.351	1.282	**0.001**
Log(Male body mass)			0.096	0.056	-0.014	0.206	0.088
Log(Female body mass)			-0.090	0.058	-0.203	0.024	0.125
ClassAves:Monogamy			0.076	0.081	-0.083	0.237	0.348
ClassMammalia:Monogamy			-0.049	0.061	-0.168	0.074	0.429
Residual variance σ^2			0.047	0.016	0.023	0.085	-
Pagel's λ			0.896	0.061	0.744	0.975	-
Intercept	-343.3	3.9	-0.506	0.239	-0.992	-0.048	**0.035**
ClassMammalia			0.892	0.320	0.279	1.520	**0.005**
Monogamy			-0.005	0.050	-0.101	0.095	0.921
ClassAves:Log(Male body mass)			0.075	0.130	-0.179	0.333	0.563
ClassMammalia:Log(Male body mass)			0.099	0.061	-0.019	0.221	0.107
ClassAves:Log(Female body mass)			-0.060	0.136	-0.328	0.208	0.661
ClassMammalia:Log(Female body mass)			-0.095	0.064	-0.221	0.028	0.137
Residual variance σ^2			0.052	0.018	0.025	0.095	-
Pagel's λ			0.912	0.056	0.767	0.981	-
Intercept	-343	4.2	-0.532	0.239	-1.015	-0.077	**0.026**
ClassMammalia			0.925	0.319	0.319	1.559	**0.004**
ClassAves:Log(Male body mass)			0.118	0.132	-0.145	0.377	0.374
ClassMammalia:Log(Male body mass)			0.089	0.062	-0.033	0.210	0.148
ClassAves:Log(Female body mass)			-0.103	0.139	-0.375	0.172	0.457
ClassMammalia:Log(Female body mass)			-0.085	0.064	-0.211	0.041	0.183
ClassAves:Monogamy			0.083	0.086	-0.085	0.252	0.332
ClassMammalia:Monogamy			-0.050	0.062	-0.170	0.073	0.414
Residual variance σ^2			0.050	0.017	0.024	0.090	-
Pagel's λ			0.905	0.058	0.757	0.978	-
Intercept	-336.7	10.5	-0.012	0.123	-0.249	0.234	0.923
Log(Male body mass)			0.086	0.058	-0.030	0.200	0.139
Log(Female body mass)			-0.073	0.060	-0.192	0.046	0.225
Monogamy			-0.022	0.049	-0.117	0.076	0.648
Residual variance σ^2			0.035	0.013	0.017	0.066	-
Pagel's λ			0.790	0.105	0.539	0.938	-
**Post-copulatory sexual selection (n = 52 mammals, 29 birds)**							
Intercept	-258.3	0.0	-0.392	0.192	-0.780	-0.022	0.041
ClassMammalia			0.628	0.249	0.151	1.124	0.012
Log(Male body mass)			0.016	0.011	-0.006	0.038	0.141

Log(Testes mass)			-0.006	0.012	-0.030	0.018	0.626
Monogamy			-0.020	0.052	-0.119	0.082	0.697
Residual variance σ^2			0.046	0.017	0.022	0.086	-
Pagel's λ			0.870	0.078	0.673	0.969	-
Intercept	-258.3	0.0	-0.408	0.188	-0.786	-0.048	0.03
ClassMammalia			0.641	0.241	0.183	1.123	0.008
Log(Male body mass)			0.016	0.011	-0.005	0.038	0.147
Log(Testes mass)			-0.006	0.012	-0.029	0.018	0.644
ClassAves:Monogamy			0.072	0.086	-0.100	0.240	0.405
ClassMammalia:Monogamy			-0.069	0.063	-0.192	0.057	0.277
Residual variance σ^2			0.043	0.016	0.021	0.081	-
Pagel's λ			0.856	0.085	0.643	0.964	-
Intercept	-254.8	3.5	-0.481	0.247	-0.982	-0.010	0.051
ClassMammalia			0.741	0.335	0.099	1.416	0.027
ClassAves:Log(Male body mass)			0.025	0.023	-0.020	0.071	0.278
ClassMammalia:log(Male body mass)			0.015	0.015	-0.015	0.044	0.321
ClassAves:Log(Testes mass)			-0.004	0.015	-0.034	0.027	0.797
ClassMammalia:Log(Testes mass)			-0.008	0.020	-0.046	0.032	0.699
ClassAves:Monogamy			0.078	0.088	-0.095	0.251	0.376
ClassMammalia:Monogamy			-0.071	0.064	-0.194	0.054	0.27
Residual variance σ^2			0.046	0.017	0.021	0.089	-
Pagel's λ			0.861	0.086	0.641	0.969	-
Intercept	-254.6	3.7	-0.432	0.251	-0.936	0.054	0.085
ClassMammalia			0.679	0.338	0.026	1.362	0.045
Monogamy			-0.019	0.052	-0.120	0.085	0.71
classAves:Log(Male body mass)			0.022	0.023	-0.023	0.067	0.347
ClassMammalia:Log(Male body mass)			0.016	0.015	-0.014	0.045	0.303
ClassAves:Log(Testes mass)			-0.006	0.016	-0.036	0.025	0.724
ClassMammalia:Log(Testes mass)			-0.006	0.020	-0.045	0.033	0.751
Residual variance σ^2			0.048	0.018	0.022	0.090	-
Pagel's λ			0.869	0.083	0.657	0.971	-
Intercept	-253.2	5.1	-0.074	0.137	-0.339	0.203	0.589
Log(Male body mass)			0.022	0.012	-0.001	0.045	0.058
Log(Testes mass)			-0.005	0.013	-0.030	0.021	0.726
Monogamy			-0.028	0.052	-0.129	0.075	0.592
Residual variance σ^2			0.036	0.014	0.017	0.073	-
Pagel's λ			0.763	0.129	0.454	0.942	-
**Cost of reproduction (n = 48 mammals, 38 birds)**							
Intercept	-298.8	0.0	-0.386	0.186	-0.765	-0.033	0.038
ClassMammalia			0.523	0.233	0.092	0.999	0.025
Log(Female body mass)			0.000	0.019	-0.036	0.037	0.992
Log(Female age at first birth)			0.013	0.033	-0.053	0.078	0.686
Log(Annual productivity)			0.006	0.021	-0.035	0.047	0.759
Female parental care			0.116	0.045	0.028	0.204	0.010
Residual variance σ^2			0.038	0.015	0.018	0.075	-
Pagel's λ			0.880	0.075	0.688	0.975	-
Intercept	-296.8	2.0	-0.405	0.230	-0.881	0.036	0.079
ClassMammalia			0.549	0.309	-0.033	1.174	0.075
Log(Female age at first birth)			0.013	0.034	-0.054	0.079	0.692
Log(Annual productivity)			0.007	0.021	-0.036	0.049	0.748
Female parental care			0.116	0.045	0.026	0.204	0.011
ClassAves:Log(Female body mass)			0.002	0.023	-0.044	0.047	0.935
ClassMammalia:Log(Female body mass)			-0.001	0.020	-0.041	0.038	0.971
Residual variance σ^2			0.040	0.015	0.019	0.076	-
Pagel's λ			0.883	0.072	0.702	0.975	-
Intercept	-295.1	3.7	-0.159	0.146	-0.438	0.140	0.278
Log(Female body mass)			0.007	0.019	-0.030	0.043	0.716
Log(Female age at first birth)			0.006	0.034	-0.059	0.072	0.856
Log(Annual productivity)			0.006	0.021	-0.035	0.047	0.772
Female parental care			0.120	0.044	0.033	0.206	0.006
Residual variance σ^2			0.030	0.011	0.015	0.057	-
Pagel's λ			0.800	0.100	0.563	0.945	-

Intercept	-294.8	4.0	-0.400	0.249	-0.903	0.068	0.109
ClassMammalia			0.545	0.352	-0.109	1.264	0.122
Log(Annual productivity)			0.006	0.022	-0.038	0.051	0.787
Female parental care			0.115	0.046	0.024	0.206	0.012
ClassAves:Log(Female body mass)			0.002	0.023	-0.044	0.048	0.930
ClassMammalia:Log(Female body mass)			0.000	0.024	-0.047	0.047	0.995
ClassAves:Log(Female age at first birth)			0.014	0.045	-0.072	0.102	0.747
ClassMammalia:Log(Female age at first birth)			0.011	0.050	-0.085	0.111	0.820
Residual variance σ^2			0.040	0.015	0.019	0.077	-
Pagel's λ			0.884	0.074	0.697	0.976	-
Intercept	-293.6	5.2	-0.280	0.287	-0.866	0.270	0.328
ClassMammalia			0.424	0.374	-0.273	1.183	0.257
Female parental care			0.118	0.045	0.027	0.208	0.009
ClassAves:Log(Female body mass)			0.018	0.031	-0.043	0.081	0.561
ClassMammalia:Log(Female body mass)			-0.008	0.026	-0.058	0.042	0.763
ClassAves:Log(Female age at first birth)			0.010	0.046	-0.081	0.098	0.826
ClassMammalia:Log(Female age at first birth)			0.022	0.051	-0.079	0.122	0.671
ClassAves:Log(Annual productivity)			-0.030	0.051	-0.132	0.069	0.557
ClassMammalia:Log(Annual productivity)			0.015	0.025	-0.033	0.064	0.536
Residual variance σ^2			0.040	0.015	0.018	0.077	-
Pagel's λ			0.880	0.076	0.688	0.974	-
Intercept	-292.4	6.4	-0.310	0.289	-0.895	0.237	0.283
ClassMammalia			0.483	0.378	-0.228	1.265	0.201
ClassAves:Log(Female body mass)			0.018	0.031	-0.043	0.078	0.572
ClassMammalia:Log(Female body mass)			-0.007	0.026	-0.059	0.045	0.802
ClassAves:Log(Female age at first birth)			0.006	0.045	-0.083	0.094	0.902
ClassMammalia:Log(Female age at first birth)			0.022	0.052	-0.080	0.123	0.667
ClassAves:Log(Annual productivity)			-0.031	0.051	-0.130	0.070	0.545
ClassMammalia:Log(Annual productivity)			0.014	0.025	-0.036	0.065	0.576
ClassAves:Female parental care			0.164	0.072	0.022	0.304	0.023
ClassMammalia:Female parental care			0.087	0.060	-0.033	0.207	0.152
Residual variance σ^2			0.040	0.016	0.018	0.078	-
Pagel's λ			0.881	0.075	0.691	0.975	-

Table S5. Parameter estimates of the models fitted using Bayesian PGLS to test the effect of hunting and sexual selection on Adult Life Expectancy (ALE) differences for the wild dataset. Columns show the deviance information criterion (DIC), the *Δ*DIC, calculated as the differences in DIC with respect to the lowest DIC model, sample sizes (N), posterior means and standard deviations (SD), and lower and upper 95% credible intervals from the posterior densities of the regression parameters. Zero overlap provides a two-sided test that indicates the area under the posterior density below or above 0.

Variable	DIC	ΔDIC	Mean	SD	Lower CI	Upper CI	Zero overlap
**Hunting on pre-copulatory sexual selection (n = 62 mammals, 39 birds)**							
Intercept	-182.8	0	-0.166	0.248	-0.689	0.296	0.504
ClassMammalia			0.407	0.280	-0.086	1.002	0.146
Hunting			-0.187	0.304	-0.782	0.404	0.537
NoHunting:Log(Male body mass)			0.101	0.127	-0.148	0.346	0.427
Hunting:Log(Male body mass)			0.229	0.267	-0.291	0.752	0.390
NoHunting:Log(Female body mass)			-0.089	0.132	-0.347	0.172	0.503
Hunting:Log(Female body mass)			-0.199	0.276	-0.738	0.343	0.470
NoHunting:Monogamy			-0.077	0.124	-0.317	0.162	0.532
Hunting:Monogamy			-0.190	0.132	-0.450	0.073	0.151
Residual variance σ^2			0.044	0.015	0.024	0.082	-
Pagel's λ			0.472	0.189	0.075	0.788	-
Intercept	-184.8	2	-0.275	0.237	-0.766	0.165	0.246
ClassMammalia			0.475	0.279	-0.024	1.055	0.088
Hunting			-0.032	0.062	-0.152	0.091	0.599
Log(Male body mass)			0.088	0.115	-0.137	0.314	0.444
Log(Female body mass)			-0.071	0.121	-0.308	0.166	0.559
Monogamy			-0.129	0.101	-0.332	0.071	0.203
Residual variance σ^2			0.046	0.016	0.025	0.085	-
Pagel's λ			0.506	0.179	0.116	0.801	-

Table S6. Order level Bayesian PGLS results for the three tested hypotheses relating to pre- and post-copulatory sexual selection and the cost of reproduction. Columns show the deviance information criterion (DIC), the *Δ*DIC, calculated as the differences in DIC with respect to the lowest DIC model, sample sizes (N), posterior means and standard deviations (SD), and lower and upper 95% credible intervals from the posterior densities of the regression parameters. Positive coefficients denote an increase in the female adult life expectancy (ALE) advantage, while negative values denote an increase in the male ALE advantage. Zero overlap provides a two-sided test that indicates the area under the posterior density below or above 0. Marsupialia includes the orders Dasyuromorphia, Peramelemorphia, Diprotodontia, and Didelphimorphia. AFB = Age at first birth. Models that are not shown did not have sufficient sample sizes to run the models (we only included analyses with a minimum sample size of 15 species per model and a minimum of 4 species per category (e.g., monogamous vs non-monogamous) for the categorical variables mating system and parental care). Pagel's λ is only shown where it could be estimated.

**Mammalia**							
**Variable**	**DIC**	**ΔDIC**	**Mean**	**SD**	**Lower CI**	**Upper CI**	**Zero overlap**
**Artiodactyla**							
**Precopulatory sexual selection (n = 123)**							
Intercept	-398.3	0	0.217	0.187	-0.149	0.589	0.247
Log(Male body mass)			0.138	0.062	0.018	0.260	**0.027**
Log(Female body mass)			-0.118	0.064	-0.243	0.005	**0.064**
Residual variance σ^2			0.040	0.010	0.024	0.063	
Pagel's λ			0.868	0.059	0.725	0.954	
Intercept	-397.5	0.8	0.248	0.188	-0.114	0.624	0.188
Log(Male body mass)			0.126	0.064	0.001	0.252	**0.049**
Log(Female body mass)			-0.109	0.065	-0.238	0.018	0.094
Monogamy			-0.098	0.089	-0.276	0.077	0.27
Residual variance σ^2			0.040	0.010	0.024	0.063	
Pagel's λ			0.869	0.058	0.728	0.954	
Intercept	-395.3	3	0.309	0.191	-0.065	0.689	0.106
Log(Female body mass)			0.017	0.013	-0.009	0.043	0.211
Monogamy			-0.132	0.091	-0.312	0.045	0.146
Residual variance σ^2			0.045	0.011	0.027	0.070	
Pagel's λ			0.895	0.051	0.770	0.966	
**Post-copulatory sexual selection (n = 74)**							
Intercept	-241.2	0	0.227	0.228	-0.214	0.683	0.319
Log(Male body mass)			-0.006	0.019	-0.044	0.030	0.748
Log(Testes mass)			0.041	0.017	0.008	0.073	**0.013**
Residual variance σ^2			0.040	0.013	0.021	0.069	
Pagel's λ			0.870	0.083	0.655	0.971	
Intercept	-240	1.2	0.284	0.235	-0.172	0.753	0.227
Log(Male body mass)			-0.011	0.019	-0.049	0.028	0.586
Log(Testes mass)			0.04	0.016	0.008	0.072	**0.014**
Monogamy			-0.099	0.129	-0.35	0.155	0.441
Residual variance σ2			0.040	0.013	0.020	0.071	
Pagel's λ			0.864	0.089	0.634	0.972	
**Cost of reproduction (n=99)**							
Intercept	-315.8	0	0.107	0.235	-0.352	0.578	0.651
Log(Female body mass)			0.008	0.027	-0.045	0.063	0.769
Log(Female AFB)			-0.097	0.054	-0.205	0.006	0.072
Log(Ann. productivity)			0.030	0.031	-0.031	0.091	0.331
Residual variance σ^2			0.037	0.011	0.020	0.064	
Pagel's λ			0.828	0.098	0.578	0.956	
Intercept	-313.9	1.9	0.26	0.214	-0.152	0.689	0.223

Log(Female body mass)			-0.015	0.024	-0.062	0.033	0.536
Log(Ann. productivity)			0.038	0.031	-0.021	0.099	0.215
Residual variance σ^2			0.040	0.012	0.021	0.067	
Pagel's λ			0.846	0.086	0.626	0.960	
**Marsupialia**							
**Pre-copulatory sexual selection (n = 44)**							
Intercept	-131.2	0.0	-0.064	0.117	-0.293	0.168	0.583
Log(Male body mass)			0.235	0.098	0.043	0.428	**0.017**
Log(Female body mass)			-0.221	0.106	-0.428	-0.012	**0.037**
Residual variance σ^2			0.023	0.008	0.012	0.044	
Pagel's λ			0.556	0.210	0.090	0.881	
**Post-copulatory sexual selection (n = 24)**							
Intercept	-77.5	0.0	-0.246	0.225	-0.690	0.195	0.274
Log(Male body mass)			0.046	0.033	-0.020	0.110	0.165
Log(Testes mass)			-0.016	0.042	-0.099	0.066	0.693
Residual variance σ^2			0.024	0.010	0.011	0.050	
Pagel's λ			0.686	0.212	0.171	0.969	
**Carnivora**							
**Pre-copulatory sexual selection (n = 94)**							
Intercept	-360.6	0.0	-0.112	0.107	-0.332	0.090	0.295
Log(Female body mass)			0.021	0.010	0.003	0.041	**0.027**
Monogamy			-0.031	0.033	-0.095	0.033	0.348
Residual variance σ^2			0.013	0.004	0.008	0.021	
Pagel's λ			0.736	0.116	0.456	0.905	
Intercept	-359.2	1.4	-0.128	0.107	-0.344	0.079	0.232
Log(Male body mass)			0.000	0.055	-0.106	0.109	0.998
Log(Female body mass)			0.022	0.056	-0.090	0.129	0.698
Residual variance σ^2			0.014	0.004	0.008	0.022	
Pagel's λ			0.749	0.108	0.490	0.906	
Intercept	-358.4	2.2	-0.112	0.106	-0.326	0.093	0.292
Log(Male body mass)			-0.007	0.056	-0.117	0.104	0.905
Log(Female body mass)			0.028	0.057	-0.084	0.141	0.626
Monogamy			-0.032	0.034	-0.099	0.035	0.35
Residual variance σ^2			0.013	0.003	0.008	0.022	
Pagel's λ			0.733	0.116	0.448	0.902	
**Post-copulatory sexual selection (n = 68)**							
Intercept	-267.4	0	-0.202	0.138	-0.480	0.061	0.144
Log(Male body mass)			0.035	0.015	0.005	0.066	**0.023**
Log(Testes mass)			-0.024	0.019	-0.060	0.013	0.191
Residual variance σ^2			0.015	0.005	0.009	0.027	
Pagel's λ			0.837	0.1	0.586	0.973	
Intercept	-265.5	1.9	-0.201	0.141	-0.48	0.072	0.155
Log(Male body mass)			0.035	0.016	0.004	0.066	**0.025**
Log(Testes mass)			-0.024	0.019	-0.06	0.012	0.191
Monogamy			0.001	0.046	-0.088	0.091	0.979
Residual variance σ^2			0.016	0.005	0.009	0.028	
Pagel's λ			0.835	0.103	0.576	0.973	
**Cost of reproduction (n = 50)**							
Intercept	-194.4	0	-0.076	0.127	-0.322	0.176	0.548
Log(Female body mass)			0.017	0.017	-0.016	0.050	0.322
Log(Ann. productivity)			-0.004	0.024	-0.051	0.043	0.875
Residual variance σ^2			0.014	0.004	0.008	0.024	
Pagel's λ			0.778	0.128	0.461	0.955	
Intercept	-193	1.4	0.017	0.077	-0.130	0.172	0.822
Log(Female AFB)			0.013	0.032	-0.050	0.076	0.676
Parental care			0.057	0.075	-0.090	0.210	0.448
Residual variance σ^2			0.014	0.004	0.008	0.024	
Pagel's λ			0.781	0.129	0.463	0.955	
Intercept	-192.4	2	-0.100	0.142	-0.381	0.176	0.478
Log(Female body mass)			0.021	0.021	-0.019	0.061	0.297

Log(Female AFB)			-0.017	0.042	-0.100	0.068	0.696
Log(Ann. productivity)			-0.005	0.024	-0.051	0.042	0.833
Residual variance σ^2			0.014	0.004	0.008	0.024	
Pagel's λ			0.775	0.133	0.438	0.954	
Intercept	-190	4.4	-0.112	0.147	-0.400	0.178	0.444
Log(Female body mass)			0.020	0.021	-0.022	0.062	0.343
Log(Female AFB)			-0.018	0.043	-0.102	0.069	0.674
Log(Ann. productivity)			-0.005	0.025	-0.055	0.044	0.829
Parental care			0.041	0.078	-0.113	0.197	0.599
Residual variance σ^2			0.014	0.004	0.008	0.025	
Pagel's λ			0.788	0.129	0.468	0.960	
**Primates**							
**Pre-copulatory sexual selection (n = 96)**							
Intercept	-337.7	0	0.179	0.140	-0.100	0.456	0.201
Log(Male body mass)			0.090	0.061	-0.029	0.210	0.138
Log(Female body mass)			-0.092	0.068	-0.226	0.040	0.174
Monogamy			-0.136	0.045	-0.224	-0.047	**0.003**
Residual variance σ^2			0.022	0.007	0.012	0.039	
Pagel's λ			0.775	0.115	0.488	0.932	
Intercept	-337.2	0.5	0.140	0.139	-0.129	0.414	0.311
Log(Female body mass)			0.006	0.015	-0.024	0.036	0.689
Monogamy			-0.148	0.044	-0.234	-0.062	**0.001**
Residual variance σ^2			0.023	0.007	0.012	0.040	
Pagel's λ			0.784	0.112	0.507	0.933	
Intercept	-329.4	8.3	0.082	0.147	-0.207	0.373	0.574
Log(Male body mass)			0.120	0.063	-0.006	0.243	0.057
Log(Female body mass)			-0.112	0.071	-0.251	0.032	0.117
Residual variance σ^2			0.025	0.008	0.013	0.045	
Pagel's λ			0.795	0.112	0.514	0.944	
**Post-copulatory sexual selection (n = 61)**							
Intercept	-212.3	0	0.144	0.155	-0.162	0.446	0.353
Log(Male body mass)			0.006	0.018	-0.030	0.042	0.753
Log(Testes mass)			0.001	0.018	-0.035	0.037	0.976
Monogamy			-0.161	0.052	-0.264	-0.059	**0.002**
Residual variance σ^2			0.029	0.010	0.014	0.053	
Pagel's λ			0.877	0.095	0.620	0.983	
Intercept	-204.2	8.1	0.031	0.165	-0.289	0.357	0.852
Log(Male body mass)			0.015	0.020	-0.023	0.053	0.441
Log(Testes mass)			0.010	0.019	-0.029	0.048	0.623
Residual variance σ^2			0.034	0.012	0.016	0.061	
Pagel's λ			0.872	0.109	0.578	0.984	
**Cost of reproduction (n = 45)**							
Intercept	-157.5	0	0.013	0.111	-0.203	0.230	0.905
Log(Female AFB)			-0.065	0.063	-0.190	0.056	0.302
Parental care			0.193	0.076	0.045	0.345	**0.011**
Residual variance σ2			0.029	0.009	0.015	0.051	
Pagel's λ			0.90	0.078	0.690	0.984	
Intercept	-154.9	2.6	-0.139	0.178	-0.497	0.212	0.435
Log(Female body mass)			0.023	0.030	-0.036	0.081	0.444
Log(Female AFB)			-0.109	0.076	-0.259	0.043	0.153
Log(Ann. productivity)			0.005	0.040	-0.073	0.084	0.899
Parental care			0.178	0.079	0.023	0.338	**0.025**
Residual variance σ^2			0.030	0.009	0.016	0.052	
Pagel's λ			0.901	0.078	0.693	0.985	
Intercept	-151.8	5.7	-0.046	0.177	-0.400	0.303	0.795
Log(Female body mass)			0.020	0.026	-0.031	0.070	0.437
Log(Ann. productivity)			0.003	0.042	-0.081	0.086	0.946
Residual variance σ^2			0.031	0.010	0.016	0.056	
Pagel's λ			0.882	0.099	0.610	0.983	
Intercept	-151	6.5	-0.075	0.185	-0.433	0.294	0.686

Log(Female body mass)			0.038	0.031	-0.021	0.099	0.21
Log(Female AFB)			-0.087	0.079	-0.242	0.072	0.271
Log(Ann. productivity)			-0.005	0.042	-0.089	0.079	0.907
Residual variance σ^2			0.032	0.01	0.016	0.056	
Pagel's λ			0.889	0.093	0.636	0.984	
**Rodentia**							
**Pre-copulatory sexual selection (n = 57)**							
Intercept	-181.6	0	-0.018	0.070	-0.159	0.119	0.8
Log(Male body mass)			0.202	0.114	-0.025	0.424	0.077
Log(Female body mass)			-0.194	0.114	-0.418	0.032	0.089
Residual variance σ^2			0.016	0.004	0.010	0.026	
Pagel's λ			0.530	0.198	0.097	0.852	
Intercept	-180.6	1	-0.013	0.070	-0.151	0.123	0.849
Log(Male body mass)			0.173	0.121	-0.065	0.413	0.152
Log(Female body mass)			-0.164	0.121	-0.405	0.075	0.175
Monogamy			-0.037	0.043	-0.121	0.049	0.391
Residual variance σ^2			0.016	0.004	0.010	0.025	
Pagel's λ			0.510	0.201	0.084	0.839	
Intercept	-180.2	1.4	0.002	0.069	-0.138	0.137	0.975
Log(Female body mass)			0.009	0.010	-0.011	0.028	0.378
Monogamy			-0.056	0.042	-0.137	0.026	0.183
Residual variance σ^2			0.016	0.004	0.010	0.026	
Pagel's λ			0.503	0.200	0.074	0.828	
**Post-copulatory sexual selection (n = 30)**							
Intercept	-85.8	0	0.020	0.146	-0.272	0.309	0.892
Log(Male body mass)			0.004	0.025	-0.046	0.055	0.874
Log(Testes mass)			0.007	0.032	-0.056	0.071	0.836
Residual variance σ^2			0.021	0.007	0.012	0.038	
Pagel's λ			0.449	0.214	0.049	0.829	
Intercept	-83.7	2.1	-0.001	0.158	-0.308	0.309	0.993
Log(Male body mass)			0.009	0.029	-0.048	0.066	0.753
Log(Testes mass)			0.001	0.035	-0.069	0.070	0.968
Monogamy			-0.039	0.084	-0.205	0.125	0.646
Residual variance σ^2			0.022	0.007	0.012	0.039	
Pagel's λ			0.436	0.215	0.042	0.820	
**Cost of reproduction (n = 25)**							
Intercept	-85.9	0	-0.112	0.094	-0.299	0.078	0.233
Log(Female body mass)			0.009	0.030	-0.051	0.069	0.774
Log(Ann. productivity)			0.018	0.039	-0.059	0.096	0.647
Residual variance σ^2			0.017	0.005	0.009	0.030	
Pagel's λ			0.724	0.236	0.139	0.992	
Intercept	-83.6	2.3	-0.144	0.121	-0.384	0.097	0.236
Log(Female body mass)			0.019	0.038	-0.057	0.096	0.627
Log(Female AFB)			-0.024	0.053	-0.129	0.081	0.651
Log(Ann. productivity)			0.010	0.044	-0.078	0.096	0.821
Residual variance σ^2			0.017	0.006	0.009	0.032	
Pagel's λ			0.702	0.242	0.127	0.991	
Intercept	-81	4.9	0.033	0.078	-0.124	0.186	0.676
Log(Female AFB)			0.019	0.043	-0.064	0.103	0.662
Parental care			0.009	0.071	-0.132	0.148	0.901
Residual variance σ^2			0.019	0.006	0.010	0.033	
Pagel's λ			0.710	0.239	0.135	0.992	
Intercept	-80.9	5	-0.135	0.128	-0.383	0.117	0.289
Log(Female body mass)			0.022	0.041	-0.061	0.104	0.587
Log(Female AFB)			-0.028	0.057	-0.140	0.083	0.627
Log(Ann. productivity)			0.007	0.046	-0.084	0.099	0.881
Parental care			-0.023	0.073	-0.169	0.119	0.752
Residual variance σ^2			0.018	0.006	0.010	0.033	
Pagel's λ			0.687	0.247	0.109	0.990	
**Aves**							

Variable	N		Mean	SD	Lower CI	Upper CI	Zero overlap
**Anseriformes**							
**Pre-copulatory sexual selection (n = 106)**							
Intercept	-419	0	-0.350	0.142	-0.633	-0.075	**0.014**
Log(Male body mass)			0.106	0.081	-0.052	0.265	0.189
Log(Female body mass)			-0.081	0.089	-0.257	0.096	0.364
Residual variance σ^2			0.021	0.006	0.012	0.035	
Pagel's λ			0.838	0.070	0.668	0.938	
Intercept	-418	0.7	-0.369	0.144	-0.653	-0.087	**0.010**
Log(Male body mass)			0.114	0.081	-0.045	0.271	0.158
Log(Female body mass)			-0.088	0.090	-0.265	0.089	0.326
Plumage dichromatism			0.017	0.019	-0.020	0.054	0.374
Residual variance σ^2			0.020	0.006	0.011	0.035	
Pagel's λ			0.828	0.075	0.646	0.935	
**Post-copulatory sexual selection (n = 32)**							
Intercept	-109.3	0.0	-0.201	0.254	-0.709	0.309	0.428
Log(Male body mass)			0.014	0.034	-0.054	0.082	0.679
Log(Testes mass)			0.007	0.019	-0.030	0.044	0.700
Residual variance σ^2			0.022	0.008	0.011	0.042	
Pagel's λ			0.763	0.166	0.330	0.968	
**Cost of reproduction (n = 76)**							
Intercept	-293.9	0	-0.428	0.226	-0.87	0.015	0.059
Log(Female body mass)			0.012	0.028	-0.044	0.066	0.666
Log(Ann. productivity)			0.025	0.044	-0.062	0.114	0.573
Residual variance σ^2			0.027	0.009	0.014	0.048	
Pagel's λ			0.884	0.063	0.727	0.965	
Intercept	-293.5	0.4	-0.179	0.092	-0.363	-0.003	0.053
Log(Female AFB)			0.029	0.031	-0.03	0.09	0.335
Parental care			0.004	0.028	-0.051	0.059	0.873
Residual variance σ^2			0.026	0.008	0.013	0.045	
Pagel's λ			0.867	0.072	0.685	0.961	
Intercept	-292.6	1.3	-0.399	0.227	-0.855	0.038	0.079
Log(Female body mass)			0.011	0.028	-0.044	0.068	0.697
Log(Female AFB)			0.022	0.031	-0.039	0.082	0.483
Log(Ann. productivity)			0.022	0.044	-0.064	0.109	0.620
Residual variance σ^2			0.027	0.009	0.014	0.048	
Pagel's λ			0.881	0.064	0.715	0.966	
Intercept	-290.7	3.2	-0.414	0.239	-0.888	0.058	0.084
Log(Female body mass)			0.01	0.028	-0.046	0.065	0.727
Log(Female AFB)			0.023	0.031	-0.038	0.084	0.468
Log(Ann. productivity)			0.025	0.046	-0.064	0.114	0.589
Parental care			0.01	0.029	-0.046	0.066	0.740
Residual variance σ^2			0.027	0.009	0.014	0.047	
Pagel's λ			0.879	0.065	0.715	0.964	
**Columbiformes**							
**Pre-copulatory sexual selection (n = 47)**							
Intercept	-160.4	0.0	-0.124	0.116	-0.353	0.102	0.284
Log(Male body mass)			-0.264	0.287	-0.833	0.302	0.357
Log(Female body mass)			0.274	0.287	-0.293	0.845	0.340
Residual variance σ^2			0.012	0.003	0.008	0.018	
Intercept	-158.6	1.8	-0.157	0.127	-0.404	0.092	
Log(Male body mass)			-0.270	0.288	-0.837	0.284	0.347
Log(Female body mass)			0.285	0.289	-0.270	0.854	0.324
Plumage dichromatism			0.037	0.055	-0.069	0.144	0.494
Residual variance σ^2			0.012	0.003	0.008	0.018	
**Galliformes**							
**Pre-copulatory sexual selection (n = 46)**							
Intercept	-140.3	0.0	-0.451	0.159	-0.763	-0.134	**0.004**

Log(Male body mass)			0.054	0.021	0.011	0.096	**0.011**
Monogamy			-0.041	0.043	-0.126	0.043	0.341
Residual variance σ^2			0.014	0.003	0.009	0.021	
Intercept	-139.8	0.5	-0.450	0.165	-0.774	-0.124	**0.006**
Log(Male body mass)			0.164	0.132	-0.097	0.424	0.214
Log(Female body mass)			-0.116	0.145	-0.407	0.167	0.424
Residual variance σ^2			0.014	0.003	0.009	0.021	
Intercept	-138.3	2.0	-0.417	0.172	-0.756	-0.078	**0.015**
Log(Male body mass)			0.130	0.136	-0.140	0.398	0.339
Log(Female body mass)			-0.084	0.149	-0.376	0.212	0.573
Monogamy			-0.035	0.046	-0.124	0.054	0.442
Residual variance σ^2			0.014	0.003	0.009	0.022	
Intercept	-137.7	2.6	-0.451	0.167	-0.783	-0.123	**0.007**
Log(Male body mass)			0.157	0.132	-0.108	0.411	0.235
Log(Female body mass)			-0.110	0.145	-0.392	0.179	0.449
Plumage dichromatism			0.010	0.033	-0.055	0.076	0.765
Residual variance σ^2			0.014	0.003	0.009	0.022	
Intercept	-136	4.3	-0.413	0.173	-0.755	-0.074	**0.017**
Log(Male body mass)			0.131	0.139	-0.145	0.401	0.347
Log(Female body mass)			-0.084	0.151	-0.380	0.215	0.579
Monogamy			-0.005	0.040	-0.083	0.072	0.906
Plumage dichromatism			-0.039	0.053	-0.143	0.066	0.457
Residual variance σ^2			0.014	0.003	0.009	0.022	
**Post-copulatory sexual selection (n = 19)**							
Intercept	-54.7	0.0	-0.728	0.278	-1.284	-0.179	**0.009**
Log(Male body mass)			0.102	0.048	0.007	0.198	**0.035**
Log(Testes mass)			-0.057	0.058	-0.171	0.057	0.320
Residual variance σ^2			0.022	0.008	0.011	0.041	
Intercept	-52.2	2.5	-0.730	0.297	-1.326	-0.143	**0.014**
Log(Male body mass)			0.102	0.050	0.007	0.201	**0.039**
Log(Testes mass)			-0.058	0.061	-0.182	0.060	0.344
Monogamy			0.000	0.094	-0.186	0.188	1.000
Residual variance σ^2			0.023	0.009	0.011	0.045	
**Cost of reproduction (n = 25)**							
Intercept	-75.1	0.0	-0.215	0.097	-0.406	-0.022	**0.026**
Log(Female AFB)			0.172	0.082	0.012	0.331	**0.035**
Parental care			0.13	0.103	-0.072	0.332	0.207
Residual variance σ^2			0.019	0.006	0.01	0.033	
Intercept	-74.6	0.5	-0.187	0.367	-0.917	0.535	0.612
Log(Female body mass)			0.073	0.045	-0.018	0.163	0.110
Log(Female AFB)			0.156	0.102	-0.046	0.357	0.125
Log(Ann. productivity)			-0.069	0.086	-0.242	0.101	0.421
Residual variance σ^2			0.018	0.006	0.01	0.033	
Intercept	-73.1	2	-0.537	0.3	-1.13	0.07	0.073
Log(Female body mass)			0.064	0.046	-0.026	0.156	0.162
Log(Ann. productivity)			0.001	0.076	-0.149	0.148	0.993
Residual variance σ^2			0.02	0.006	0.011	0.035	
Intercept	-72.6	2.5	-0.321	0.441	-1.2	0.545	0.467
Log(Female body mass)			0.055	0.055	-0.056	0.162	0.317
Log(Female AFB)			0.149	0.104	-0.058	0.354	0.154
Log(Ann. productivity)			-0.037	0.104	-0.241	0.17	0.723
Parental care			0.075	0.127	-0.176	0.329	0.557
Residual variance σ^2			0.019	0.006	0.01	0.035	
**Passeriformes**							
**Pre-copulatory sexual selection (n = 77)**							
Intercept	-197.6	0.0	-0.084	0.093	-0.268	0.099	0.369
Log(Male body mass)			0.034	0.019	-0.003	0.071	0.074
Monogamy			-0.082	0.057	-0.194	0.031	0.150
Residual variance σ^2			0.019	0.003	0.014	0.026	
Intercept	-196.2	1.4	-0.143	0.078	-0.299	0.009	0.066

Log(Male body mass)			0.248	0.225	-0.202	0.686	0.269
Log(Female body mass)			-0.223	0.238	-0.686	0.253	0.350
Residual variance σ^2			0.019	0.003	0.014	0.026	
Intercept	-195.7	1.9	-0.075	0.095	-0.263	0.113	0.429
Log(Male body mass)			0.151	0.235	-0.308	0.616	0.521
Log(Female body mass)			-0.125	0.248	-0.613	0.360	0.615
Monogamy			-0.072	0.060	-0.193	0.045	0.232
Residual variance σ^2			0.019	0.003	0.014	0.026	
Intercept	-194.2	3.4	-0.157	0.085	-0.323	0.011	0.066
Log(Male body mass)			0.237	0.224	-0.202	0.680	0.290
Log(Female body mass)			-0.209	0.238	-0.679	0.260	0.379
Plumage dichromatism			0.014	0.034	-0.053	0.083	0.674
Residual variance σ^2			0.019	0.003	0.014	0.027	
Intercept	-193.7	3.9	-0.048	0.127	-0.300	0.199	0.707
Log(Male body mass)			0.135	0.242	-0.342	0.617	0.578
Log(Female body mass)			-0.110	0.253	-0.612	0.387	0.664
Monogamy			-0.015	0.042	-0.098	0.068	0.726
Plumage dichromatism			-0.087	0.077	-0.238	0.063	0.256
Residual variance σ^2			0.019	0.003	0.014	0.027	
**Post-copulatory sexual selection (n = 37)**							
Intercept	-86.2	0.0	-0.074	0.206	-0.478	0.332	0.720
Log(Male body mass)			0.026	0.042	-0.057	0.109	0.531
Log(Testes mass)			0.041	0.046	-0.049	0.132	0.374
Residual variance σ^2			0.024	0.006	0.015	0.038	
Intercept	-83.9	2.3	-0.080	0.229	-0.532	0.376	0.726
Log(Male body mass)			0.027	0.043	-0.058	0.112	0.527
Log(Testes mass)			0.041	0.047	-0.050	0.133	0.374
Monogamy			0.004	0.088	-0.169	0.174	0.963
Residual variance σ^2			0.024	0.006	0.015	0.039	
**Cost of reproduction (n = 22)**							
Intercept	-50.9	0	-0.164	0.172	-0.505	0.178	0.339
Log(Female body mass)			0.1	0.07	-0.038	0.238	0.157
Log(Ann. productivity)			-0.072	0.095	-0.265	0.111	0.447
Residual variance σ^2			0.027	0.01	0.015	0.051	
Intercept	-48.8	2.1	-0.093	0.249	-0.579	0.4	0.707
Log(Female body mass)			0.068	0.103	-0.136	0.273	0.507
Log(Female AFB)			0.046	0.109	-0.17	0.26	0.675
Log(Ann. productivity)			-0.058	0.102	-0.258	0.149	0.571
Residual variance σ^2			0.029	0.01	0.015	0.054	
**Psittaciformes**							
**Pre-copulatory sexual selection (n = 72)**							
Intercept	-218.3	0.0	-0.114	0.086	-0.282	0.055	0.182
Log(Male body mass)			0.218	0.220	-0.221	0.651	0.323
Log(Female body mass)			-0.206	0.224	-0.650	0.240	0.358
Residual variance σ^2			0.013	0.002	0.009	0.018	
Intercept	-217.9	0.4	-0.056	0.098	-0.250	0.137	0.570
Log(Male body mass)			0.199	0.221	-0.238	0.631	0.367
Log(Female body mass)			-0.198	0.225	-0.633	0.246	0.379
Plumage dichromatism			-0.048	0.039	-0.125	0.030	0.226
Residual variance σ^2			0.013	0.002	0.009	0.018	

Table S7. Parameter estimates of the models fitted using Bayesian PGLS to test the effect of plumage dichromatism on Adult Life Expectancy (ALE) differences for the wild dataset. Results are shown for all birds, and separately for the orders Anseriformes and the remaining orders. Plumage dichromatism was measured in the interval [-2, 2], where negative scores denote a female-bias in mean relative brightness and patterning, 0 denotes no difference, and positive values denote a male bias, following Székely et al. (2022). Columns show sample sizes (N), posterior means and standard deviations (SD), and lower and upper 95% credible intervals from the posterior densities of the regression parameters. Zero overlap provides a two-sided test that indicates the area under the posterior density below or above 0.

Variable	Mean	SD	Lower CI	Upper CI	Zero overlap
**All birds (n = 41)**					
Intercept	-0.090	0.097	-0.283	0.103	0.349
Plumage dichromatism	-0.164	0.065	-0.290	-0.035	**0.011**
Residual variance σ^2	0.036	0.012	0.020	0.067	-
Pagel's λ	0.538	0.207	0.104	0.872	-
**Anseriformes (n = 17)**					
Intercept	-0.118	0.127	-0.371	0.137	0.352
Plumage dichromatism	-0.211	0.094	-0.396	-0.029	**0.024**
Residual variance σ^2	0.033	0.016	0.014	0.075	-
Pagel's λ	0.645	0.221	0.119	0.947	-
**Other orders (n = 24)**					
Intercept	-0.119	0.113	-0.346	0.106	0.292
Plumage dichromatism	0.001	0.132	-0.262	0.262	0.992
Residual variance σ^2	0.040	0.014	0.021	0.075	-
Pagel's λ	0.360	0.217	0.021	0.789	-

Table S8. Parameter estimates of the models fitted using Bayesian PGLS to test the influence of different measures of mating system in birds on Adult Life Expectancy (ALE) differences for the sexual selection hypotheses. Columns show the deviance information criterion (DIC), posterior means and standard deviations (SD), and lower and upper 95% credible intervals from the posterior densities of the regression parameters. Zero overlap provides a two-sided test that indicates the area under the posterior density below or above 0. Model 1 analyzes the mating system scores (0-4) for both sexes in birds, following methods from Székely et al. (2022). Models 2-4 (birds only) and 5-8 (combined classes) group species as monogamous or non-monogamous using thresholds where ≥ 80%, ≥ 95% and ≥ 99% of individuals of both sexes are monogamous within a species.

Analysis	Variable	DIC	ΔDIC	Mean	SD	Lower CI	Upper CI	Zero overlap
**N = 392 birds**								
Model 1: Ungrouped scores (0-4) for males and females	Intercept	-1225.0	5.7	-0.192	0.069	-0.329	-0.057	**0.006**
	Log(Male body mass)			0.062	0.055	-0.046	0.170	0.257
	Log(Female body mass)			-0.046	0.057	-0.157	0.066	0.416
	Monogamy male			-0.013	0.006	-0.024	-0.001	**0.034**
	Monogamy female			0.002	0.009	-0.016	0.020	0.800
	Residual variance σ^2			0.014	0.002	0.012	0.018	-
	Pagel's λ			0.432	0.092	0.244	0.603	-
Model 2: ≥80% threshold for monogamy	Intercept	-1230.7	0.0	-0.17	0.064	-0.299	-0.048	**0.008**
	Log(Male body mass)			0.072	0.05	-0.024	0.172	0.145
	Log(Female body mass)			-0.057	0.051	-0.159	0.044	0.268
	Monogamy			-0.063	0.022	-0.106	-0.02	**0.004**
	Residual variance σ^2			0.014	0.002	0.012	0.018	-
	Pagel's λ			0.434	0.089	0.255	0.603	-
Model 3: ≥95% threshold for monogamy	Intercept	-1228.6	2.1	-0.18	0.064	-0.309	-0.059	**0.005**
	Log(Male body mass)			0.079	0.049	-0.017	0.177	0.108
	Log(Female body mass)			-0.063	0.051	-0.166	0.036	0.212
	Monogamy			-0.051	0.02	-0.092	-0.011	**0.012**
	Residual variance σ^2			0.014	0.002	0.011	0.018	-
	Pagel's λ			0.431	0.09	0.249	0.598	-
Model 4: ≥99% threshold for monogamy	Intercept	-1226.5	4.2	-0.197	0.063	-0.323	-0.078	**0.002**
	Log(Male body mass)			0.084	0.049	-0.012	0.18	0.088
	Log(Female body mass)			-0.068	0.051	-0.168	0.031	0.183
	Monogamy			-0.035	0.017	-0.068	-0.001	**0.040**
	Residual variance σ^2			0.014	0.002	0.011	0.018	-
	Pagel's λ			0.429	0.092	0.242	0.601	-
**N = 392 birds, 456 mammals**								
Model 5: ≥80% threshold for monogamy	Intercept	-2756.4	0.0	-0.329	0.075	-0.476	-0.184	**<0.001**
	ClassMammalia			0.587	0.091	0.411	0.765	**<0.001**
	Log(Male body mass)			0.106	0.024	0.059	0.155	**<0.001**
	Log(Female body mass)			-0.095	0.025	-0.145	-0.046	**<0.001**
	Monogamy			-0.063	0.015	-0.092	-0.034	**<0.001**
	Residual variance σ^2			0.029	0.004	0.022	0.039	-
	Pagel's λ			0.77	0.043	0.675	0.843	-
Model 6: ≥95% threshold for monogamy	Intercept	-2754.3	2.1	-0.336	0.075	-0.484	-0.19	**<0.001**
	ClassMammalia			0.594	0.09	0.416	0.769	**<0.001**
	Log(Male body mass)			0.109	0.024	0.061	0.156	**<0.001**
	Log(Female body mass)			-0.098	0.025	-0.147	-0.048	**<0.001**

	Monogamy			-0.057	0.014	-0.085	-0.029	**<0.001**
	Residual variance σ^2			0.029	0.004	0.022	0.039	-
	Pagel's λ			0.767	0.044	0.672	0.843	-
Model 7: ≥99% threshold for monogamy	Intercept	-2751.1	5.3	-0.35	0.075	-0.497	-0.204	**<0.001**
	ClassMammalia			0.603	0.09	0.427	0.779	**<0.001**
	Log(Male body mass)			0.112	0.024	0.065	0.159	**<0.001**
	Log(Female body mass)			-0.101	0.025	-0.149	-0.052	**<0.001**
	Monogamy			-0.046	0.013	-0.071	-0.021	**0.002**
	Residual variance σ^2			0.029	0.004	0.022	0.038	-
	Pagel's λ			0.766	0.043	0.671	0.839	-

Table S9. Parameter estimates of the models fitted using Bayesian PGLS to test the influence of different measures of parental care in birds on Adult Life Expectancy (ALE) differences for the cost of reproduction hypothesis. Columns show the deviance information criterion (DIC), the *Δ*DIC, calculated as the differences in DIC with respect to the lowest DIC model, posterior means and standard deviations (SD), and lower and upper 95% credible intervals from the posterior densities of the regression parameters. Zero overlap provides a two-sided test that indicates the area under the posterior density below or above 0. Model 1 tests the ungrouped continuous parental care variable ranging from -2 to 2, where -2 refers to male-only care, and 2 refers to female-only care. Models 2 and 3 test a grouped variable with three categories (male care, biparental, and female care) at different cutoff values denoted in the table. Model 4 shows the final model included in this analysis, which only includes two categories (male/biparental and female care). Male care and biparental care were combined because they showed no statistical difference.

Analysis	Variable	DIC	ΔDIC	Mean	SD	Lower CI	Upper CI	Zero overlap
**PGLS on 385 birds**								
Model 1: Continuous variable	Intercept	-1260.5	1.7	-0.130	0.037	-0.205	-0.062	**<0.001**
	Log(Female age at first birth)			0.042	0.013	0.017	0.067	**0.001**
	Parental care			0.016	0.009	-0.002	0.034	0.080
	Residual variance σ^2			0.014	0.002	0.011	0.018	-
	Pagel's λ			0.49	0.099	0.281	0.663	-
Model 2: Grouped into 3 categories at -1 and 1	Intercept	-1257.8	4.4	-0.140	0.039	-0.219	-0.067	**<0.001**
	Log(Female age at first birth)			0.042	0.013	0.017	0.067	**0.001**
	Female care			0.021	0.015	-0.010	0.051	0.180
	Male care			0.047	0.058	-0.066	0.161	0.414
	Residual variance σ^2			0.014	0.002	0.011	0.018	-
	Pagel's λ			0.492	0.098	0.283	0.664	-
Model 3: Grouped into 3 categories at -0.5, and 0.5	Intercept	-1262.2	0.0	-0.143	0.037	-0.219	-0.073	**<0.001**
	Log(Female age at first birth)			0.042	0.013	0.017	0.068	**0.001**
	Female care			0.039	0.016	0.009	0.071	**0.013**
	Male care			0.031	0.039	-0.047	0.107	0.433
	Residual variance σ^2			0.013	0.002	0.010	0.018	-
	Pagel's λ			0.481	0.100	0.274	0.661	-
Model 4: Grouped into 2 categories (male/biparental and female care at <0)	Intercept	-1260.8	1.4	-0.141	0.037	-0.215	-0.070	**<0.001**
	Log(Female age at first birth)			0.041	0.013	0.015	0.066	**0.002**
	Female care			0.029	0.015	-0.001	0.059	0.060
	Residual variance σ^2			0.014	0.002	0.010	0.018	-
	Pagel's λ			0.510	0.097	0.301	0.682	-

Data S1.

R code, life tables, ALE differences, aggregated life history data, and data from populations in the wild can be found at: https://doi.org/10.5281/zenodo.15183248
